# Photocatalytic stannylation of white phosphorus†

**DOI:** 10.1039/d2cc03474c

**Published:** 2022-07-14

**Authors:** Marion Till, Jose Cammarata, Robert Wolf, Daniel J. Scott

**Affiliations:** University of Regensburg, Institute of Inorganic Chemistry 93040 Regensburg Germany robert.wolf@ur.de; University of Oxford, Department of Chemistry OX1 3TA Oxford UK daniel.scott@chem.ox.ac.uk

## Abstract

Organophosphorus compounds (OPCs) are highly important chemicals, finding numerous applications in both academia and industry. Herein we describe a simple photocatalytic method for the stannylation of white phosphorus (P_4_) using a cheap, commercially-available distannane, (Bu_3_Sn)_2_, and anthraquinone as a simple photocatalyst. Subsequent ‘one pot’ transformation of the resulting stannylated monophosphine intermediate (Bu_3_Sn)_3_P provides direct, convenient and versatile access to valuable OPCs such as acylated phosphines and tetraalkylphosphonium salts.

## Abstract 2

A simple, mechanistically unique photochemical procedure is reported for the efficient, direct, catalytic stannylation of P_4_ and ‘one pot’ transformation into valuable monophosphorus compounds.

## Graphical abstract

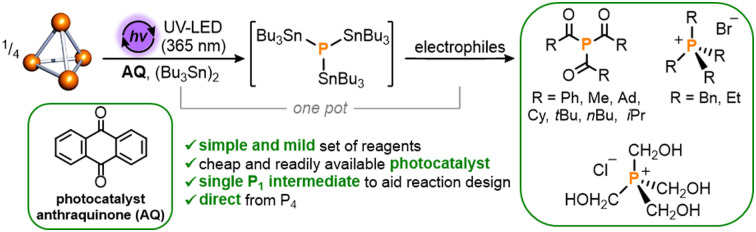

White phosphorus (P_4_) – the most chemically important allotrope of this ubiquitous and abundant element – acts as the common precursor from which all commercially valuable and academically important organophosphorus compounds (OPCs) are prepared. The current methods used for the industrial synthesis of these myriad useful P_1_ products include the oxidation of P_4_ with toxic Cl_2_ gas to generate PCl_3_ which can subsequently be transformed into a variety of OPCs by reaction with nucleophiles (Scheme 1a). As an alternative route, initial acid- or base-mediated disproportionation of P_4_ can be used to generate highly toxic PH_3_ gas which is then employed for the hydrophosphination of unsaturated organic substrates.^1^

**Scheme 1:**
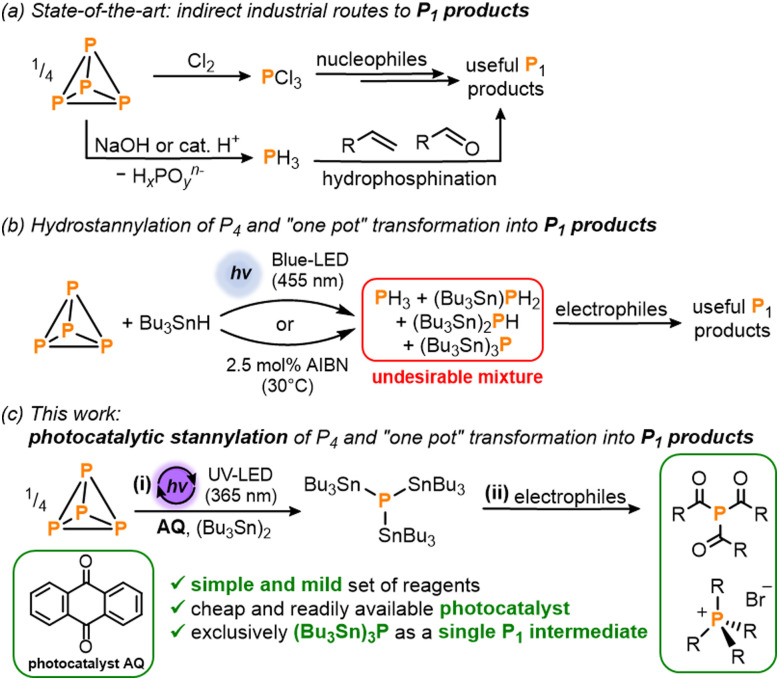
(a) Current state-of-the-art industrial methods for the synthesis of valuable P_1_ products.^1^ (b) Recently reported hydrostannylation of white phosphorus (P_4_) using Bu_3_SnH followed by reaction with electrophiles to generate useful P_1_ products in a ‘one-pot’ fashion.^5^ (c) This work: (i) photocatalytic stannylation of P_4_ using the photocatalyst anthraquinone (AQ) and hexabutyldistannane (Bu_3_Sn)_2_; and (ii) subsequent functionalization of the intermediate (Bu_3_Sn)_3_P with electrophiles into products such as triacylphosphines and tetraalkylphosphonium salts.

Given the drawbacks of these methods, a highly prominent aim has long been to find ways of bypassing these multi-step procedures. In particular, there is a longstanding desire to develop more step-efficient *direct* – and, ideally, *catalytic* – methods to functionalize P_4_ and generate OPCs in a single reaction.

As a result, for several decades comprehensive efforts have been made to better understand the fundamental reactivity of P_4_.^2^ However, it is only very recently that it has finally become possible to successfully transform P_4_ directly into a variety of useful P_1_ products.^3^ Moreover, and despite these extensive investigations, the number of successful examples remains extremely low, and those that do exist still suffer from substantial limitations.^4^ As such, there remains a clear need to expand the range of strategies available for direct, productive P_4_ activation, with new catalytic methods being particularly desirable.^4*a*^

In one of our own contributions to this area, we recently reported a simple ‘one pot’ method in which the classical radical reagent tri-*n*-butyltin hydride (Bu_3_SnH) is used for initial hydrostannylation of P_4_ (Scheme 1b).^5^ This reductive P_4_ activation is mediated either by light or by a chemical radical initiator such as AIBN (azobis(isobutyronitrile)) which can initiate a radical chain reaction that breaks down the P_4_ tetrahedron, yielding a mixture of hydrostannylated phosphines (Bu_3_Sn)_*x*_PH_3−*x*_ (*x* = 0–3). Key to this mechanism is the attack of stannyl radicals (Bu_3_Sn˙) on the P–P bonds of P_4_. The resulting (Bu_3_Sn)_*x*_PH_3−*x*_ mixture can then be converted into a number of important and useful OPCs by reaction with electrophiles.^5^

Unfortunately, one significant disadvantage of this hydrostannylation strategy is the complexity of the (Bu_3_Sn)_*x*_PH_3−*x*_ mixture, which complicates ‘downstream’ reaction development by requiring functionalization of two different types of bond (P–Sn and P–H), both of which are distributed over four distinct molecules. Moreover, the presence of gaseous PH_3_ as a component of this mixture has been suggested to have a limiting effect on overall yields as it can easily be lost during subsequent manipulations,^4*a*^ and it is also problematic from a safety perspective.

These drawbacks would be overcome if the initial P_4_ reduction step could instead furnish a single species with just one functionalizable motif, but with reactivity otherwise similar to (Bu_3_Sn)_*x*_PH_3−*x*_. To achieve this, we describe herein a simple photocatalytic strategy for the atom-precise stannylation of P_4_ using the cheap, commercially-available distannane (Bu_3_Sn)_2_ and simple benzophenone derivatives as photocatalysts (Scheme 1c). This new procedure generates exclusively the stannylated monophosphine (Bu_3_Sn)_3_P and subsequent, simplified ‘one pot’ transformations with electrophiles afford valuable OPCs including acylated phosphines and alkylated phosphonium salts.

Based on the analysis above, we sought to develop a new method by which P_4_ could be selectively transformed into (Bu_3_Sn)_3_P as the sole product.^6^ It is worth noting that the closely related product (Ph_3_Sn)_3_P has previously been prepared from P_4_ using Ph_3_SnCl as the stannylating reagent, but this required use of a relatively elaborate Ti(iii) reagent as a halogen atom abstractor.^3*f*^ Instead, we imagined that an ideal reagent for such a reaction would be the distannane (Bu_3_Sn)_2_, which is cheap to purchase and could in principle provide the target phosphine with perfect atom economy.^7^ Indeed, Sn–Sn homolysis of (Bu_3_Sn)_2_ is known to furnish Bu_3_Sn˙ radicals, which previous work has shown are capable of adding to P_4_.^3*f*,5^ However, achieving this homolysis directly requires extreme temperatures or very high energy UV light irradiation that is known to lead to unselective reactivity, and is also unlikely to be compatible with P_4_.^8–10^ Fortunately, it has been reported that simple ketones can be used as photocatalysts to access Bu_3_Sn˙ radicals by Sn–Sn bond cleavage under much lower energy irradiation.^11^

The light-driven photocatalytic stannylation of P_4_ was therefore targeted, based on the mechanistic proposal outlined in Scheme 2.^9^ It was anticipated that photoirradiation of the ketone R_2_CO would first provide an excited state, [R_2_CO]*,^12^ capable of reacting with (Bu_3_Sn)_2_ to generate a stannylated ketyl radical and a free Bu_3_Sn˙ radical.^11^ The former could then thermally release a second Bu_3_Sn˙ radical to close the catalytic cycle. Once formed, these Bu_3_Sn˙ radicals would then add to the P–P bonds of P_4_, ultimately breaking it down to generate (Bu_3_Sn)_3_P as the only P_1_ product.^13^

**Scheme 2:**
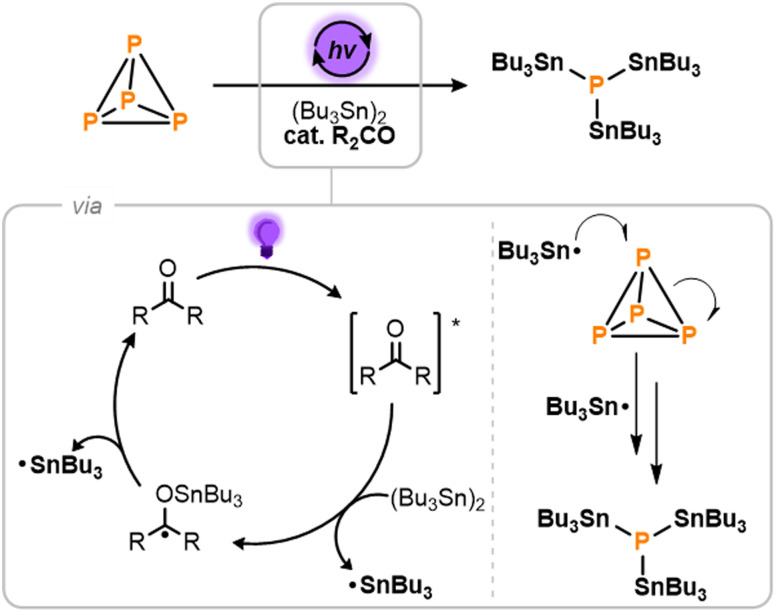
Proposed mechanism for the light-driven, photocatalytic stannylation of P_4_ in the presence of hexabutyldistannane, (Bu_3_Sn)_2_, and a ketone photocatalyst, R_2_CO.

To begin, benzophenone (BP) was chosen as a proof-of-principle photocatalyst due to both its simplicity and the fact that its photoreactivity towards hexaalkyldistannanes has been studied previously.^11*d*^ Gratifyingly, after an initial optimization the photocatalytic stannylation of P_4_ could successfully be achieved, with use of 25 mol% BP (all stoichiometries, in both equiv. and mol%, are defined per P atom) and a 3.3-fold excess (5 equiv.) of (Bu_3_Sn)_2_ providing 50% conversion to the target stannylated phosphine (Bu_3_Sn)_3_P after stirring under near UV LEDs overnight (Scheme 3; see also ESI,† S3). Control experiments confirmed that all reaction components (P_4_, (Bu_3_Sn)_2_, BP, irradiation) were necessary for the reaction to proceed productively (see ESI,† S3, Table S1).

**Scheme 3:**
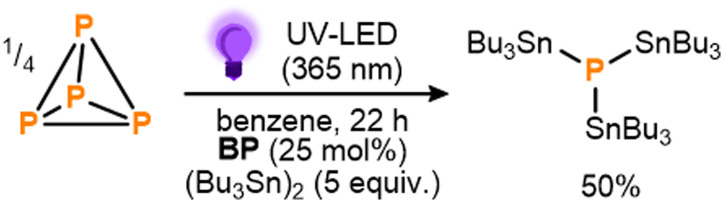
Initial conditions for the direct, photocatalytic stannylation of P_4_ into (Bu_3_Sn)_3_P optimized using benzophenone (BP) as photocatalyst. Stoichiometries in equiv. and mol% are defined per P atom.

These initial results provided a clear proof-of-principle for the proposed mechanistic strategy. Notably, the observed conversion indicates the activation of at least three Sn–Sn bonds per available equivalent of BP,^14^ making this a rare example of a system where P_4_ activation has been achieved catalytically, using an otherwise inert substrate.^5,9*a*,9*b*,9*e*,15^ Nevertheless, in order to improve the reaction outcome further, a broader range of benzophenone derivates was subsequently screened, with several found to provide markedly improved performance (see ESI,† S5). Particularly impressive results were achieved using anthraquinone (AQ) and following brief further optimization (see ESI,† S5 and S7) 79% conversion to (Bu_3_Sn)_3_P could be achieved using significantly reduced loadings of both AQ (12.5 mol%) and (Bu_3_Sn)_2_ (3 equiv.) over the same timeframe (Scheme 4; see also ESI,† S7). Based on the catalytic cycle proposed in Scheme 2, this would correspond to a turnover number (TON) of 10.0 for AQ. Further reductions in catalyst loading to 6.3 mol% or 2.5 mol% were found to lead to even higher TONs (16.8 and 28.2, respectively), albeit at the cost of lower overall conversions (see ESI,† S7, Table S11).

**Scheme 4:**
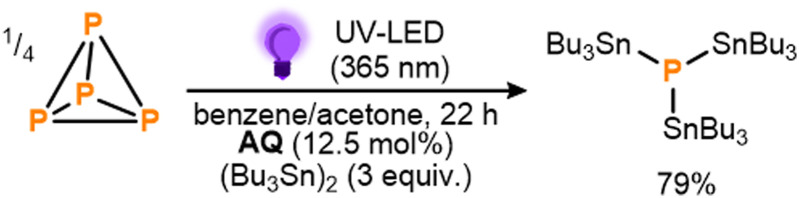
Optimized conditions for the direct, photocatalytic stannylation of P_4_ into (Bu_3_Sn)_3_P using anthraquinone (AQ) as photocatalyst. Stoichiometries in equiv. and mol% are defined per P atom.

With the stannylation of P_4_ optimized, attention was then shifted to its subsequent, ‘one pot’ transformation into other useful P_1_ products. Having previously developed procedures for the analogous transformation of the phosphine mixture (Bu_3_Sn)_*x*_PH_3−*x*_, which includes (Bu_3_Sn)_3_P as a minor component, it was anticipated that addition of electrophiles to photocatalytically-generated (Bu_3_Sn)_3_P should be similarly productive,^4,5^ especially since neither the AQ photocatalyst nor the (Bu_3_Sn)_2_ starting material is expected to show appreciable reactivity towards such substrates. And, indeed, *in situ* addition of a variety of acid chlorides yielded the corresponding triacylphosphines (R(O)C)_3_P (R = Ph, Cy, Ad, *t*Bu, *i*Pr, *n*Bu, Me) with good conversions of up to 75% (Scheme 5a(i)).^5,16^ Notably, and in comparison to our previously-reported hydrostannylation system, no exclusion of light and no additional base were required for this step, highlighting both the robustness and simplicity of (Bu_3_Sn)_3_P as a “P^3−^” synthon, relative to (Bu_3_Sn)_*x*_PH_3−*x*_.

**Scheme 5:**
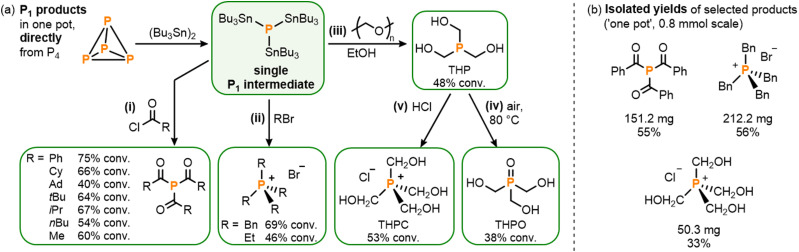
(a) One-pot synthesis directly from P_4_, *via* photocatalytically generated P_1_ intermediate (Bu_3_Sn)_3_P, of (i) triacylphosphines (R(O)C)_3_P (4 equiv. RC(O)Cl, R = *t*Bu, Ph, Me, *n*Bu, Cy, *i*Pr, Ad), (ii) phosphonium salts [R_4_P]Br (5 equiv. RBr, R = Bn, Et, 60–80 °C), (iii) tris(hydroxymethyl)phosphine, THP (EtOH, 3 equiv. paraformaldehyde), (iv) tris(hydroxymethyl)phosphine oxide, THPO (as for (iii) then air, 80 °C), and (v) tetrakis(hydroxymethyl)phosphonium chloride, THPC (as for (iii) using 12.5 equiv. paraformaldehyde, then 10 equiv. HCl); and (b) Isolated yields for reactions on preparative scale (0.8 mmol). Stoichiometries in equiv. are defined per P atom.

Similarly, reaction of (Bu_3_Sn)_3_P with alkyl bromides RBr (R = Bn, Et) under moderate heating successfully provided ‘one pot’ access to the corresponding phosphonium salts, [R_4_P]Br, including tetrabenzylphosphonium bromide, [Bn_4_P]Br, which is a known precursor for useful Wittig chemistry (Scheme 5a(ii)).^17^ Again, no auxiliary base was required for these reactions, in contrast to the analogous procedures *via* (Bu_3_Sn)_*x*_PH_3−*x*_ where the absence of base leads to a 50% reduction in yield.^5^

Finally, another industrially important class of P_1_ products was targeted. Hydroxymethyl-substituted phosphine derivatives are used as flame-retardant materials (among a number of other applications),^18^ and could be accessed by reacting the stannylated monophosphine (Bu_3_Sn)_3_P with paraformaldehyde in EtOH to furnish tris(hydroxymethyl)phosphine, (HOCH_2_)_3_P (THP; Scheme 5a(iii)).^18*a*^ Subsequent exposure to air then yielded the corresponding phosphine oxide, (HOCH_2_)_3_PO (THPO; Scheme 5a(iv)),^18*b*^ while the phosphonium salt tetrakis(hydroxymethyl)phosphonium chloride, [(HOCH_2_)_4_P]Cl (THPC),^18*c*,18*d*^ could be accessed by quenching the *in situ* generated THP with HCl, all in one pot (Scheme 5a(v)).

To demonstrate the viability of these reactions on a preparative scale the triacylphosphine (Ph(O)C)_3_P and the phosphonium salts [Bn_4_P]Br and THPC were selected as representative examples for isolation (Scheme 5b; see ESI† S9). At 0.8 mmol scale (PhC(O))_3_P could be isolated in 55% yield,^19^ which compares well with our previously-reported hydrostannylation method (51%). [Bn_4_P]Br could also be isolated in good 56% yield, and THPC in a more modest yield of 33%.^19^

For this last reaction, efforts were also made to recover the Sn-containing compounds present at the end of the reaction. We have previously shown that for the analogous synthesis of THPC *via* (Bu_3_Sn)_*x*_PH_3−*x*_ recovery of the Bu_3_SnCl byproduct allows for convenient regeneration and recycling of the Bu_3_SnH starting material, thus minimizing the formation of organotin-containing waste. Bu_3_SnCl can also be used to regenerate (Bu_3_Sn)_2_ through a net one-electron reduction,^8^ meaning similar recycling should be feasible for this newer system, provided Bu_3_SnCl can again be cleanly recovered. Satisfyingly, Bu_3_SnCl could indeed be recovered during THPC workup through simple washing with diethyl ether, being isolated as part of an otherwise clean mixture with unreacted (Bu_3_Sn)_2_ in an excellent overall yield of 92% (1.3 : 1 molar ratio, see ESI† S9).

In conclusion, we have developed a simple, new method for the direct transformation of P_4_ into a variety of commercially and academically interesting OPCs. The reaction proceeds through a photocatalytic stannylation of white phosphorus, which generates (Bu_3_Sn)_3_P with perfect atom economy as a single, convenient P_1_ intermediate using an inexpensive, commercially available distannane and a simple photocatalyst. This method can be used to prepare a variety of different products through inclusion of a range of different electrophilic substrates, and we have demonstrated that the Sn-containing byproducts of the reaction can in principle be recovered and recycled. These results expand the currently very limited range of strategies that are available for the direct functionalization of P_4_, and suggest the intriguing possibility that P_4_ activation might also be achievable by reaction with other weak E–E bonds under similar photocatalytic conditions.

The project received funding from the European Research Council (ERC CoG 772299). DJS is also grateful to the Alexander von Humboldt foundation, and to the EPSRC for award of an Early Career Fellowship (EP/V056069/1).

## Conflicts of interest

There are no conflicts to declare.

## Supplementary Material

CC-058-D2CC03474C-s001
