# Supplementary material for: Photocatalytic stannylation of white phosphorus
Source: Chem Commun (Camb). 2022 Jul 14;58(64):8986–9. doi: 10.1039/d2cc03474c (PMC9362875; doi:10.1039/d2cc03474c)
Supplement: CC-058-D2CC03474C-s001 [file CC-058-D2CC03474C-s001.pdf]

Electronic Supplementary Material (ESI) for ChemComm.
This journal is © The Royal Society of Chemistry 2022

Supplementary Information

Photocatalytic Stannylation of White Phosphorus

Marion Till[1], Jose Cammarata[1], Robert Wolf*[1] and Daniel J. Scott*[2]

[1]Institute of Inorganic Chemistry, University of Regensburg, 93040 Regensburg, Germany

E-mail: robert.wolf@ur.de

[2]Department of Chemistry, University of Oxford, OX1 3TA Oxford, UK

E-mail: daniel.scott@chem.ox.ac.uk

Table of contents

S1. General information 2

S2. Supplementary mechanistic discussion 4

S3. General procedure for photocatalytic functionalization of P_4 (0.04 mmol scale) into stannylated phosphine $(Bu_3Sn)_3P$ using benzophenone 7

S4. Optimization of photocatalytic reaction conditions using benzophenone 9

S5. Benzophenone derivatives used as photocatalysts for the photocatalytic functionalization of P_4 to $(Bu_3Sn)_3P$ 12

S6. General procedure for photocatalytic functionalization of P_4 (0.04 mmol scale) into stannylated phosphine $(Bu_3Sn)_3P$ using anthraquinone 19

S7. Optimization of photocatalytic reaction conditions using anthraquinone 21

S8. Characterization of optimized 0.04 mmol scale reactions using anthraquinone 24

S9. Reactions at 0.8 mmol scale using anthraquinone as a photoinitiator 35

S10. References 47

S1. General information

All reactions and manipulations were performed under an N_2 atmosphere (< 0.1 ppm O_2, H_2O) through use of a GS Glovebox (GS117717). All glassware was oven-dried (160 °C) overnight prior to use. Benzene was distilled from Na/benzophenone and stored over molecular sieves (3 Å). Acetonitrile was distilled from CaH_2 and stored over molecular sieves (3 Å). Acetone was stirred over $CaSO_4$ (30 min) and dynamically dried by passing over molecular sieves (3 Å). THF was purified using an MBraun SPS-800 system and stored over molecular sieves (3 Å). C_6D_6 was distilled from K and stored over molecular sieves (3 Å). Hexabutyldistannane $(Bu_3Sn)_2$ was purchased from ABCR or Thermo Scientific (97% purity). The photocatalysts benzophenone, 4-benzoylpyridine, xanthone, thioxanthone, anthraquinone and DDQ (2,3-dichloro-5,6-dicyano-1,4-benzochinone) were ordered from major chemical suppliers (Sigma, ABCR) and used as received. All other chemicals were also purchased from major suppliers (Aldrich, ABCR); liquids were purified by Kugelrohr distillation and freeze-pump-thaw degassed three times prior to use; P_4 and Ph_3PO were purified by sublimation; all others were used as received.

Qualitative NMR spectra were recorded at room temperature on Bruker Avance III HD 400 (400 MHz) spectrometers and were processed using Topspin 3.2. Chemical shifts δ, are reported in parts per million (ppm); ^{1}H and ^{13}C shifts are reported relative to $SiMe_4$ and were calibrated internally to residual solvent peaks, while ^{31}P shifts and ^{119}Sn shifts were referenced externally to 85 % H_3PO_4 (aq.) and Me_4Sn, respectively. NMR samples were prepared in the glovebox using NMR tubes fitted with screw caps. Optimization reactions (see sections S5, S6 and S7) and photocatalytic stannylation of P_4 to $(Bu_3Sn)_3P$ and subsequent functionalization to P_1 products were analyzed by $^{31}P\{^{1}H\}$ spectra using triphenylphosphine, Ph_3PO, as a standard.

For determining the conversion of the P_1 products, the following **quantitative $^{31}P\{^{1}H\}$ NMR pulse programs** were used:

- Triacylphosphines[1] → **$T1_{IS} < T1_{Prod}$ = 10s, D1 = 40 s, zgig30, inverse gated decoupled, LB = 10**
- Phosphonium salts[2] → **single scan, DS = 0, D1 = 2 s, D1 ≥ 5 x T1, zgig90, inverse gated decoupled, LB = 1.**

LEDs used for the optimization reactions of the photocatalytic stannylation of P_4 to $(Bu_3Sn)_3P$ were:

- Green LED: 528 nm, 3.5 V, 700 mA, Osram OSLON SSL 80
- Blue LED: 455 nm (±15 nm), 3.2 V, 700 mA, Osram OSLON SSL 80
- Violet/Blue LED: 415 nm (±5 nm), 3.5 V, 700 mA, Intelligent LED Solutions ILH-XC01-5410-SC211-WIR200
- Violet LED: 405 nm (±10 nm), 4.0 V, 700 mA, Edixeon EDEV-SLC1-03
- UV LED: 385nm (±15 nm), 3,4 V, 350 mA LST1-01G01-UV02-00
- UV LED: 365 nm, 4.3 V, 700 mA, Osram OSLON SSL 80
- UV LED: 365 nm, 14 V, 700 mA, Osram OSLON SSL 80 (scale-up reactions)

S2. Supplementary mechanistic discussion

The proposed mechanism for the photocatalytic stannylation of P_4 by $(Bu_3Sn)_2$ catalyzed by simple ketones is outlined in Scheme 2 of the primary manuscript. However, it should be noted that in the absence of more comprehensive mechanistic studies it is currently not possible to exclude the involvement of several alternative elementary reaction steps. For example, while the formation of an intermediate stannylated ketyl radical $[R_2COSnBu_3]^•$ is supported by the prior literature,[3] an alternative energy transfer from $[R_2CO]^*$ to $(Bu_3Sn)_2$ resulting in cleavage to $Bu_3Sn^•$ by a purely outer sphere mechanism has not yet been definitively excluded.

One key question left unanswered at present relates to the precise mechanism by which radical breakdown of the P_4 tetrahedron is achieved. Based on our previous studies it can be confidently proposed that each P–P bond cleavage step is likely to begin with attack of a photocatalytically-generated $Bu_3Sn^•$ radical (Scheme S1).[1] The simplest subsequent step would be recombination of the resulting P-centered radical with a second $Bu_3Sn^•$ radical, thus completing the stannylation of the original P–P moiety (Scheme S1a). Note, however, that this requires bimolecular recombination of two transient radicals whose concentrations are expected to be low.

Alternatively, stannylation of this P-centered radical could be achieved through S_H2-type attack on the proposed intermediate $[R_2COSnBu_3]^•$ and/or its pinacol-type dimer, examples of which are known from the literature to exist in equilibrium (Scheme S1b).[3] In this scenario, this equilibrium would act as a stabilized and hence more persistent reservoir of chemically-accessible "$Bu_3Sn^•$".

A third scenario is that S_H2-type attack could occur directly on the $(Bu_3Sn)_2$ substrate, which should be present in high concentrations throughout the reaction. This scenario would result in a radical chain mechanism analogous to that proposed previously for P_4 hydrostannylation (Scheme S1c).[1] In this case the R_2CO photocatalyst would formally act as a photosensitizing initiator for the chain reaction, rather than as a strict catalyst *per se*. However, we currently consider this to be a less likely option, due to several qualitative experimental observations. Firstly, attempts to achieve an analogous chain reaction mechanism using "standard" chemical initiators such as AIBN under thermal conditions (instead of R_2CO under photochemical conditions) have thus far been unsuccessful, not leading to any identifiable change by $^{31}P\{^1H\}$ NMR spectroscopy (for example, see Figure S1).

Secondly, attempts to initiate the same radical chain under thermal conditions by using catalytic quantities of both AIBN and Bu_3SnH, which should definitely result in formation of $Bu_3Sn^•$, have also been unsuccessful. These led only to trace <u>hydro</u>stannylation, consistent with the amount of Bu_3SnH

present and similar to the outcome when no distannane is present, which argues against any efficient radical interception by $(Bu_3Sn)_2$ (for example, see Figure S2).

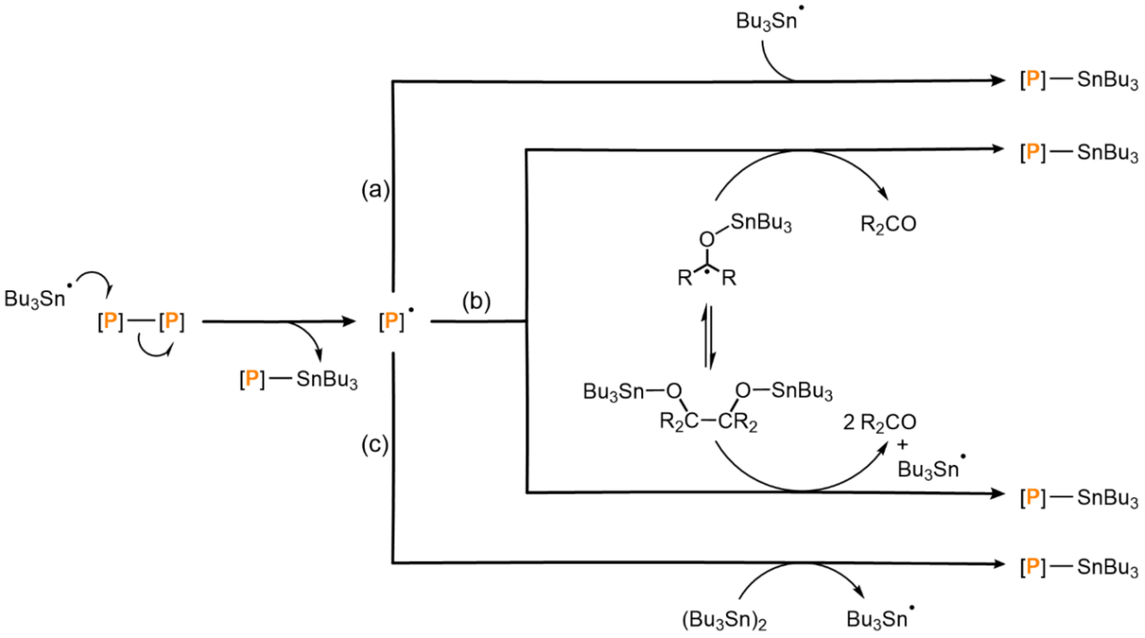

Scheme S1. Illustration of the equipment setup used for photocatalytic reactions at 0.04 mmol scale.

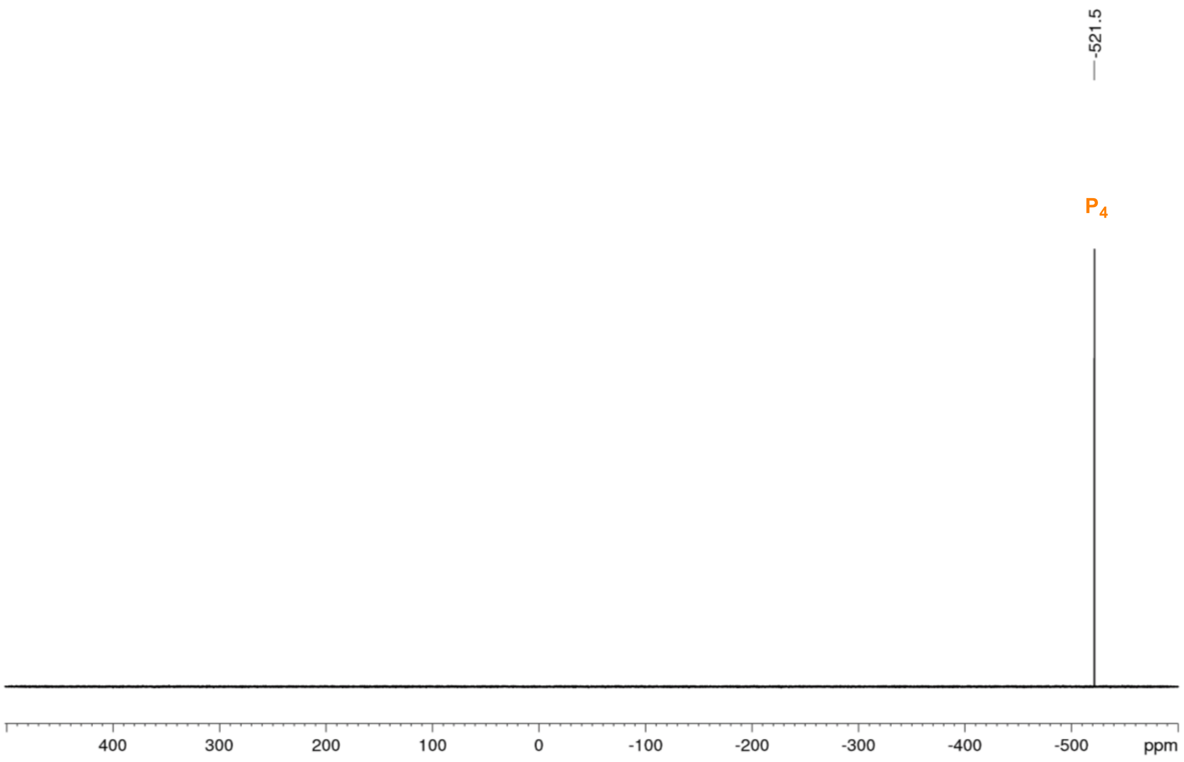

Figure S1. Representative $^{31}P\{^{1}H\}$ NMR spectrum for the attempted stannylation of P_4 using $(Bu_3Sn)_2$ and 12.5 mol% AIBN in toluene, after heating to 80 °C for 16 h.

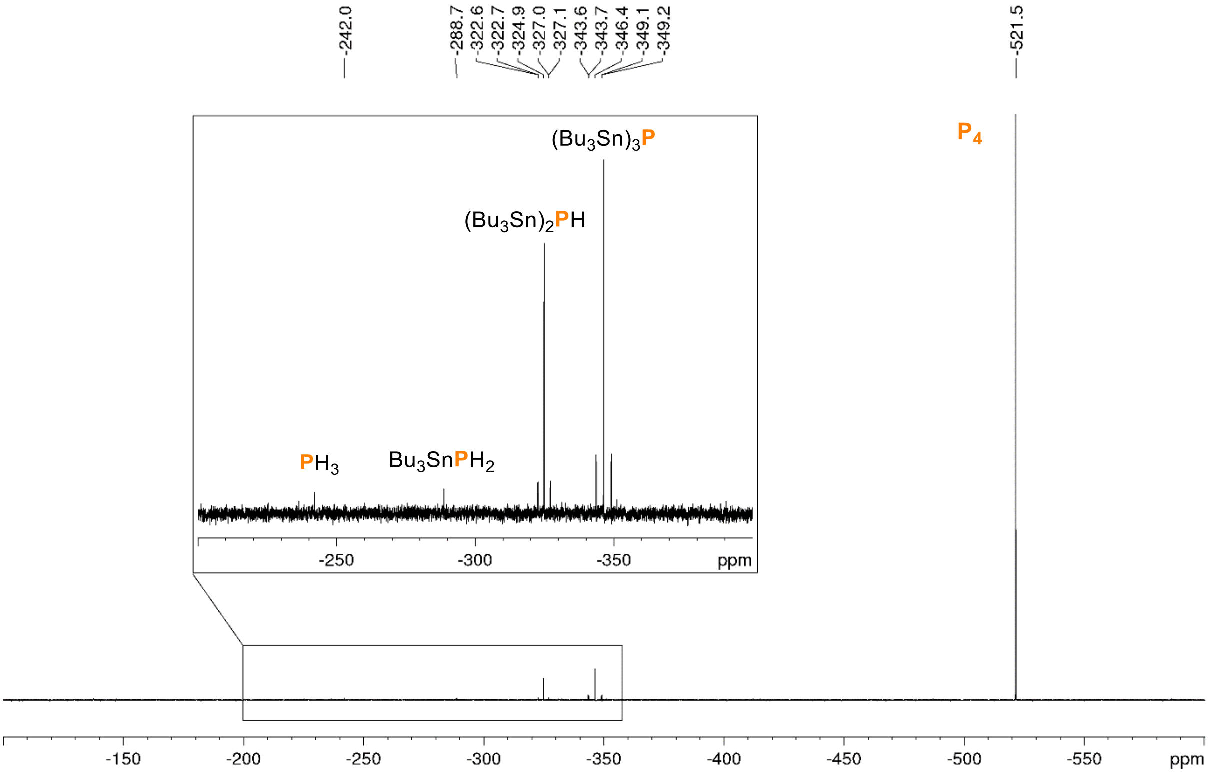

Figure S2. Representative $^{31}P\{^{1}H\}$ NMR spectrum for the attempted stannylation of P_4 using $(Bu_3Sn)_2$ and 12.5 mol% of both AIBN and Bu_3SnH in toluene, after heating to 80 °C for 18 h. The relatively high proportion of $(Bu_3Sn)_2PH$ and $(Bu_3Sn)_3P$ relative to Bu_3SnPH_2 (*c.f.* ref. [1]) can likely be attributed to Bu_3Sn/H ligand scrambling at the elevated reaction temperature and/or the high loading of AIBN relative to Bu_3SnH which reduces the number of available H atoms. This is supported by the observation that when the reaction is repeated in the absence of $(Bu_3Sn)_2$ the resulting $(Bu_3Sn)_xPH_{3-x}$ mixture is also heavily weighted towards $(Bu_3Sn)_2PH$ and $(Bu_3Sn)_3P$.

S3. General procedure for photocatalytic functionalization of P_4 (0.04 mmol scale) into stannylated phosphine $(Bu_3Sn)_3P$ using benzophenone

At the start of this project, benzophenone (**BP**) was chosen as a photocatalyst for the initial reaction optimization due to both its simplicity and the fact that its (photo)reactivity towards hexaalkyldistannanes has been studied previously.[3]

To a 10 mL stoppered tube equipped with a stirring bar were added $(Bu_3Sn)_2$ (101.1 µL, 5 equiv. based on phosphorus atoms, 20 equiv. based on P_4), **BP** (0.01 mmol, as a stock solution in 149.2 µL benzene, 1 equiv. based on P_4) and P_4 (0.01 mmol, as a stock solution in 71.3 µL benzene) in benzene as solvent (in total 0.5 mL). The tube was sealed, placed in a water-cooled block (to ensure a near-ambient temperature was maintained, Figure S3),[2] and irradiated with UV light (365 nm, 4.3 V, 700 mA, Osram OSLON SSL 80) for 22 h (unless stated otherwise). Ph_3PO (0.02 mmol, stock solution in benzene) was subsequently added to act as an internal standard. The resulting mixture was subjected to $^{31}P\{^1H\}$ NMR analysis (Figure S4). Formation of $(Bu_3Sn)_3P$ was indicated by the characteristic $^{117/119}Sn$-satellited resonance at -346.5 ppm.[1]

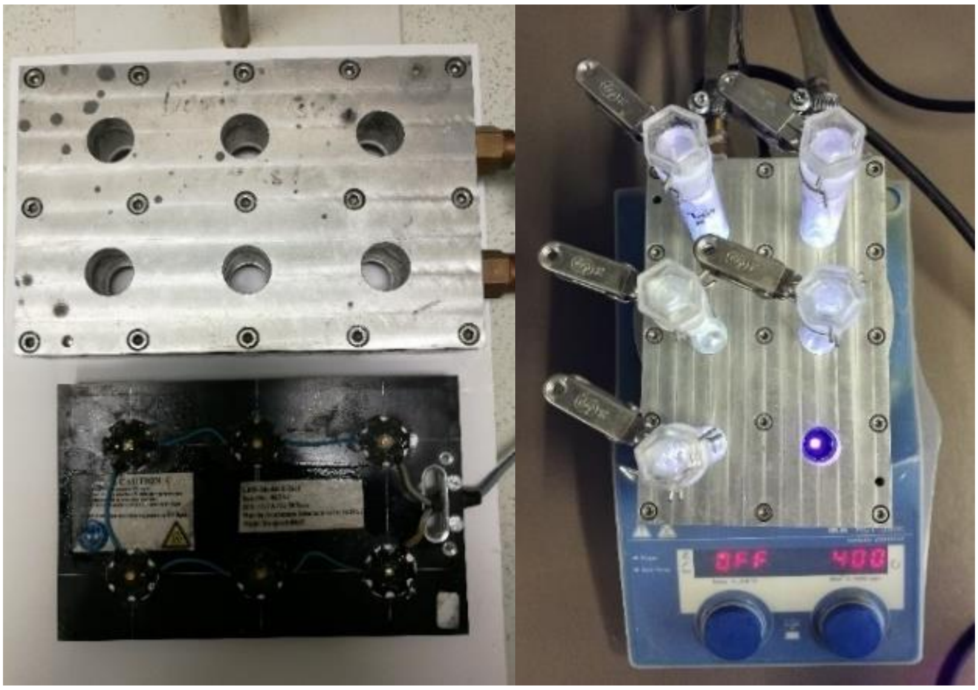

Figure S3. Illustration of the equipment setup used for photocatalytic reactions at 0.04 mmol scale.

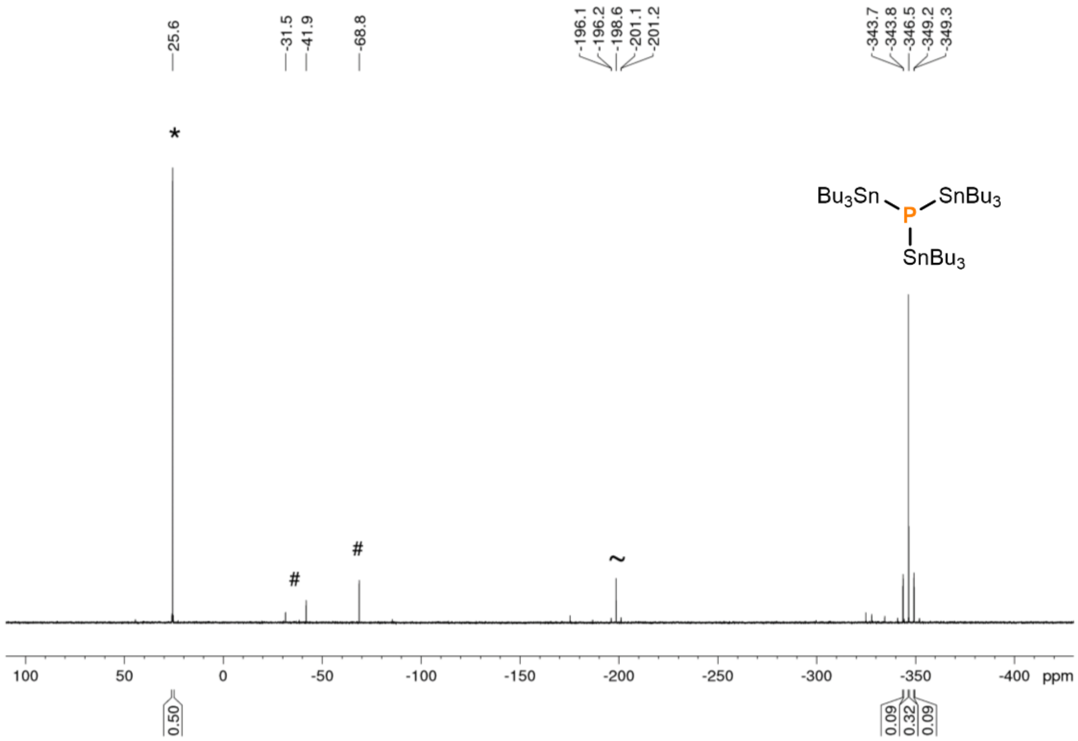

Figure S4. Representative $^{31}P\{^{1}H\}$ NMR spectrum for the photocatalytic functionalization of P_4 using **benzophenone** (**BP**) as a photocatalyst (Table 1, Entry 1). ***** marks the internal standard Ph_3PO (0.02 mmol). **~** marks an unknown Sn-containing side product. **#** marks unknown side products.

S4. Optimization of photocatalytic reaction conditions using benzophenone

Table S1. Photocatalytic functionalization of P_4 to $(Bu_3Sn)_3P$: screening of **control experiments**.[a]

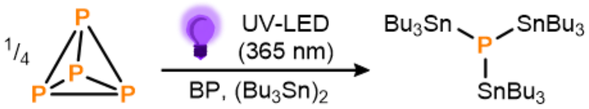

**Entry**	Conditions	Full conv. of $\mathbf{P_4}$?	Conv. to $\mathbf{(Bu_3Sn)_3P}$ / %
1	Standard[a]	✓	50
2	No light	X	0
3	No benzophenone	✓	0
4	No $(Bu_3Sn)_2$	X	0
5	Blue LEDs (455 nm)	X	0
6	Violet LEDs (405nm)	✓	26
7	P_{red} instead of P_4	–	1

[a] For the general procedure, see section S3.

Table S2. Photocatalytic functionalization of P_4 to $(Bu_3Sn)_3P$: screening of different **radiation sources (LEDs)**.[a]

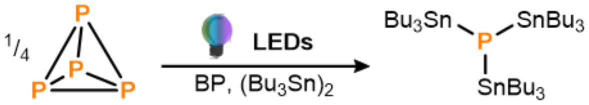

**Entry**	**LEDs**	Full conv. of $\mathbf{P_4}$?	Conv. to $\mathbf{(Bu_3Sn)_3P}$ / %
**1**	**365 nm[a] (UV LED)**	✓	**50**
2	385 nm (UV LED)	✓	40
3	405 nm (violet LED)	✓	26
4	420 nm (violet blue LED)	✓	21
5	455 nm (blue LED)	X	0
6	528 nm (green LED)	X	0

[a] The general procedure (see section S3) was modified to use the LEDs indicated.

Table S3. Photocatalytic functionalization of P_4 to $(Bu_3Sn)_3P$: screening of **irradiation period**.[a]

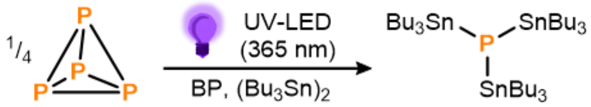

Entry	Irradiation period	Full conv. of P_4?	Conv. to $(Bu_3Sn)_3P$ / %
1	16 h	✓	41
2	18 h	✓	43
3	20 h	✓	45
**4**	**22 h**	✓	**50**

[a] The general procedure (see section S3) was modified in the period of irradiation with UV light (365 nm).

Table S4. Photocatalytic functionalization of P_4 to $(Bu_3Sn)_3P$: screening of **solvents**.[a]

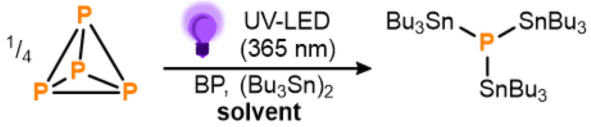

Entry	Solvent	Full conv. of P_4?	Conv. to $(Bu_3Sn)_3P$ / %
**1**	**Benzene**[a]	✓	**50**
2	Benzene[b]	✓	47
**3**	**Acetone**	✓	**50**
4	Acetonitrile	✓	35
5	THF	✓	11
6	Toluene	✓	48

[a] The general procedure (see section S3) was modified to use a solvent mixture (P_4 and **BP** stock solutions still in benzene, total solvent volume of 0.5 mL). [b] 0.22 mL solvent volume.

Table S5. Photocatalytic functionalization of P_4 to $(Bu_3Sn)_3P$: screening of **amounts of benzophenone**.[a][b]

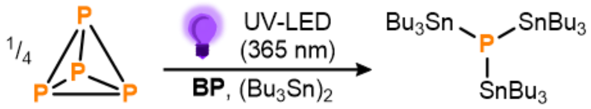

Entry	**BP** / equiv.[b]	Full conv. of P_4?	Conv. to $(Bu_3Sn)_3P$ / %
1	0.5	✓	45
2	1	✓	50
3	4	✓	60
4	10	✓	59

[a] For the general procedure, see section S3. [b] Listed equivalents are defined per P_4 molecule.

Table S6. Photocatalytic functionalization of P_4 to $(Bu_3Sn)_3P$: screening of **amounts of $(Bu_3Sn)_2$**.[a][b]

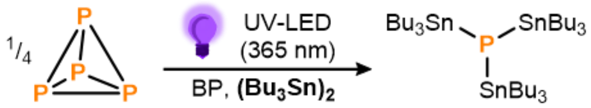

Entry	$(Bu_3Sn)_2$ / equiv.[a]	Full conv. of P_4?	Conv. to $(Bu_3Sn)_3P$ / %
1	30	✓	58
2	20	✓	51
3	15	✓	49
4	12	✓	45
5	6	✓	35

[a] For the general procedure, see section S3. [b] Listed equivalents are defined per P_4 molecule.

S5. Benzophenone derivatives used as photocatalysts for the photocatalytic functionalization of P_4 to $(Bu_3Sn)_3P$

Table S7. Photocatalytic functionalization of P_4 to $(Bu_3Sn)_3P$: screening of **benzophenone derivatives.**[4][a]

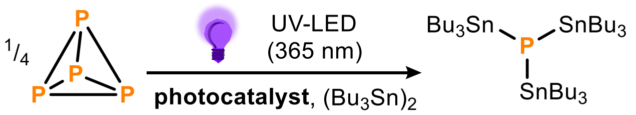

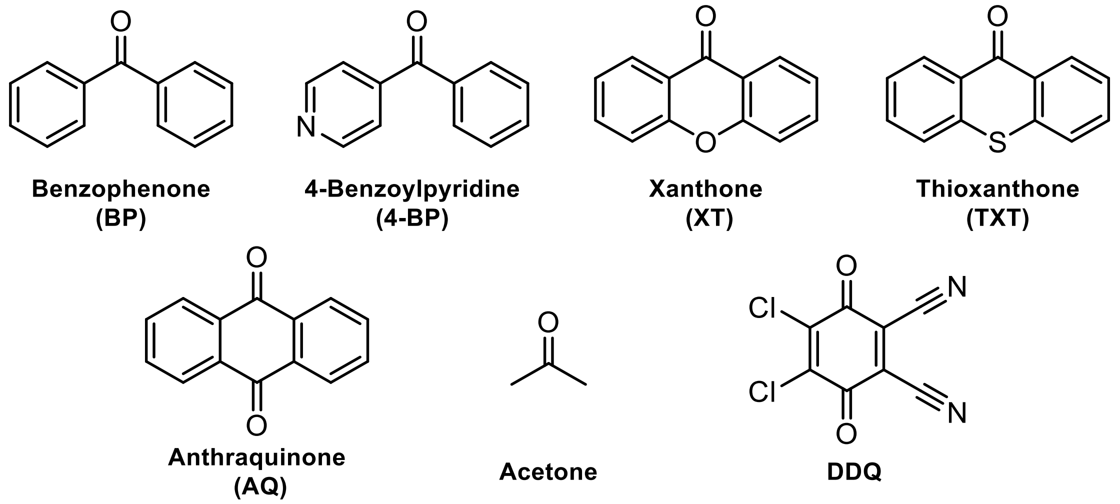

Entry	Photoinitiator (PI)	Full conv. of P_4?	Conv. to $(Bu_3Sn)_3P$ / %
1	Benzophenone[a] (**BP**)	✓	50
2	Benzophenone[b] (**BP**)	✓	50
3	4-Benzoylpyridine (**4-BP**)	✓	73
4	Xanthone (**XT**)	✓	39
5	Thioxanthone (**TXT**)	✓	70
6	Thioxanthone[b] (**TXT**)	✓	66
7	Anthraquinone (**AQ**)	✓	62
8	Anthraquinone[b] (**AQ**)	✓	75
**9**	**Anthraquinone**[b][c] (**AQ**)	✓	**83**
10	Acetone	✓	2
11	**DDQ**	✓	8

[a] For the general procedure, see section S3. [b] A solvent mixture of benzene (71.3 µL of the P_4 stock solution) and acetone (429 µL) was used. [c] Instead of the usual 1 equiv. of the photocatalyst only 0.5 equiv. of the anthraquinone (**AQ**) was used.

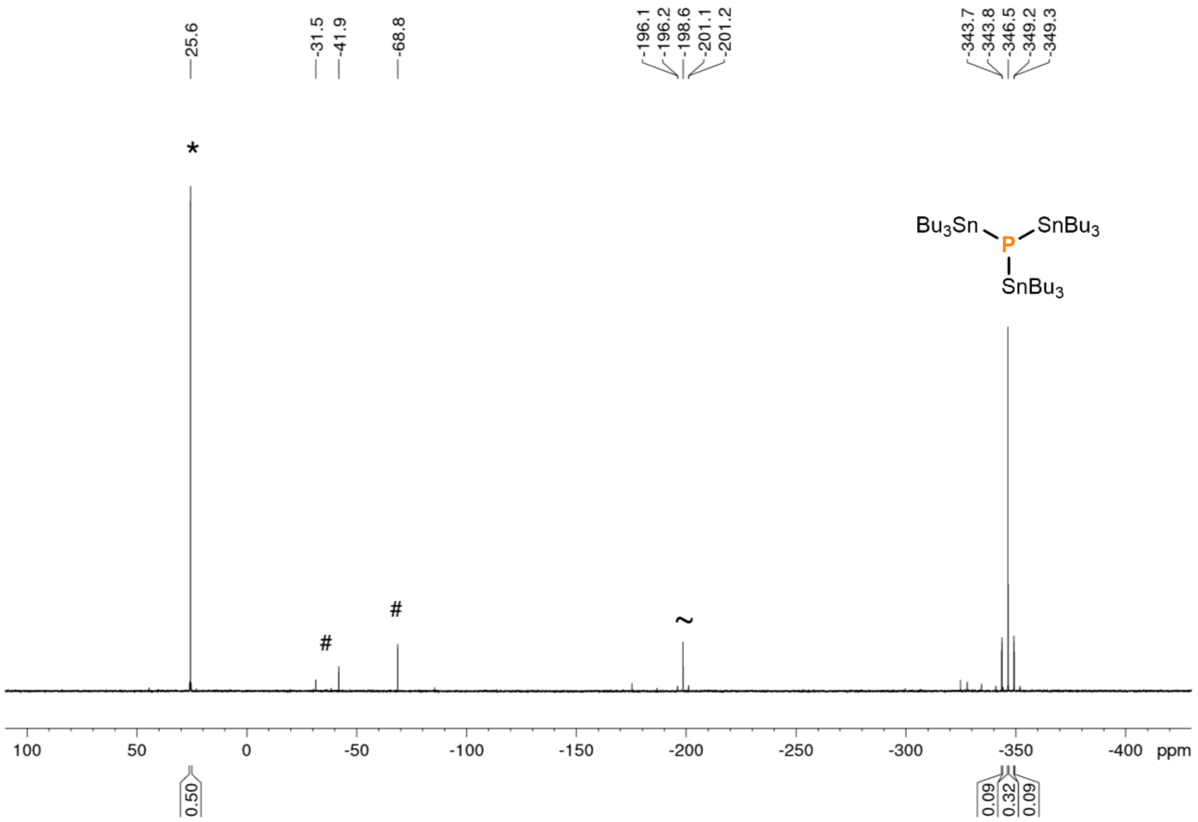

Figure S5. $^{31}P\{^{1}H\}$ NMR spectrum for the photocatalytic functionalization of P_4 using **benzophenone** (**BP**) as a photocatalyst (Table 7, Entry 1). ***** marks the internal standard Ph_3PO (0.02 mmol). **~** marks an unknown Sn-containing side product. **#** marks unknown side products.

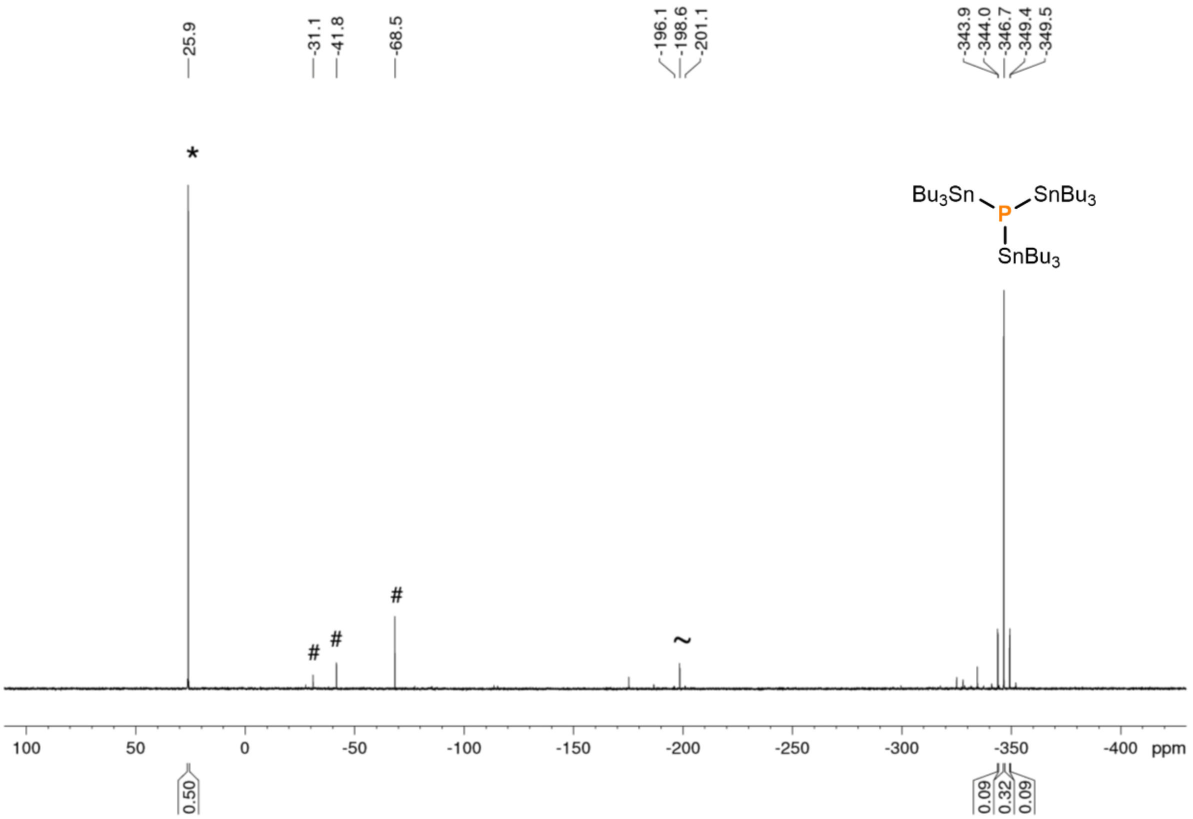

Figure S6. $^{31}P\{^{1}H\}$ NMR spectrum for the photocatalytic functionalization of P_4 using **benzophenone** (**BP**) as a photocatalyst (Table 7, Entry 2, **PhH/acetone**). ***** marks the internal standard Ph_3PO (0.02 mmol). **~** marks an unknown Sn-containing side product. **#** marks unknown side products.

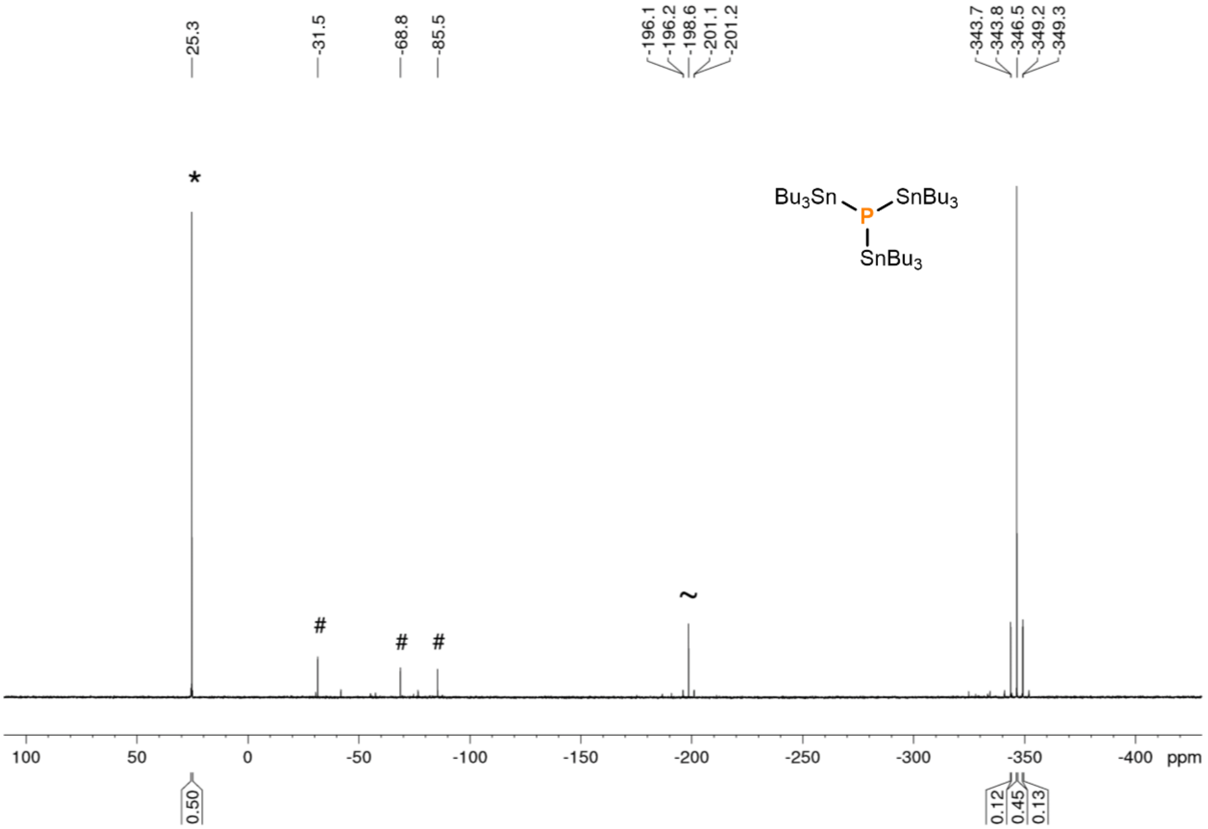

Figure S7. $^{31}P\{^{1}H\}$ NMR spectrum for the photocatalytic functionalization of P_4 using **4-benzoylpyridine** (**4-BP**) as a photocatalyst (Table S7, Entry 3). ***** marks the internal standard Ph_3PO (0.02 mmol). **~** marks an unknown Sn-containing side product. **#** marks unknown side products.

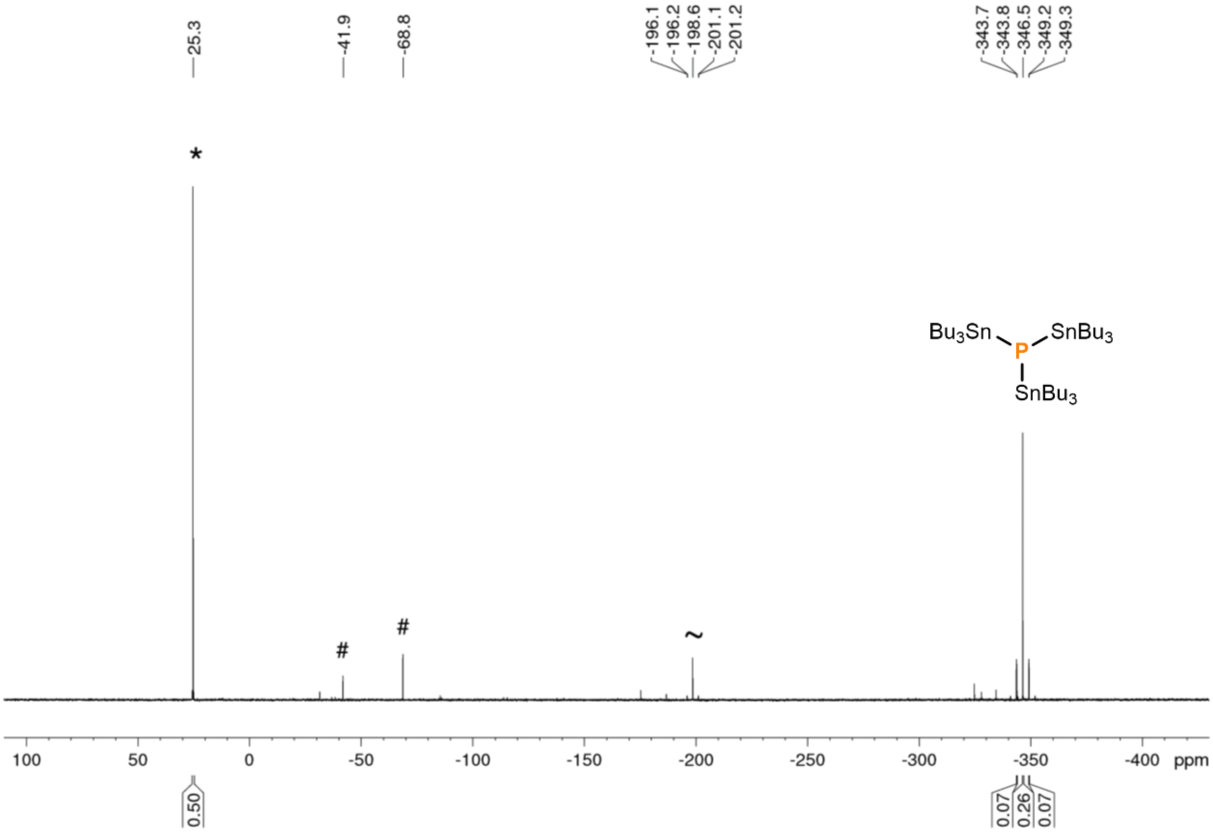

Figure S8. $^{31}P\{^{1}H\}$ NMR spectrum for the photocatalytic functionalization of P_4 using **xanthone** (**XT**) as a photocatalyst (Table S7, Entry 4). ***** marks the internal standard Ph_3PO (0.02 mmol). **~** marks an unknown Sn-containing side product. **#** marks unknown side products.

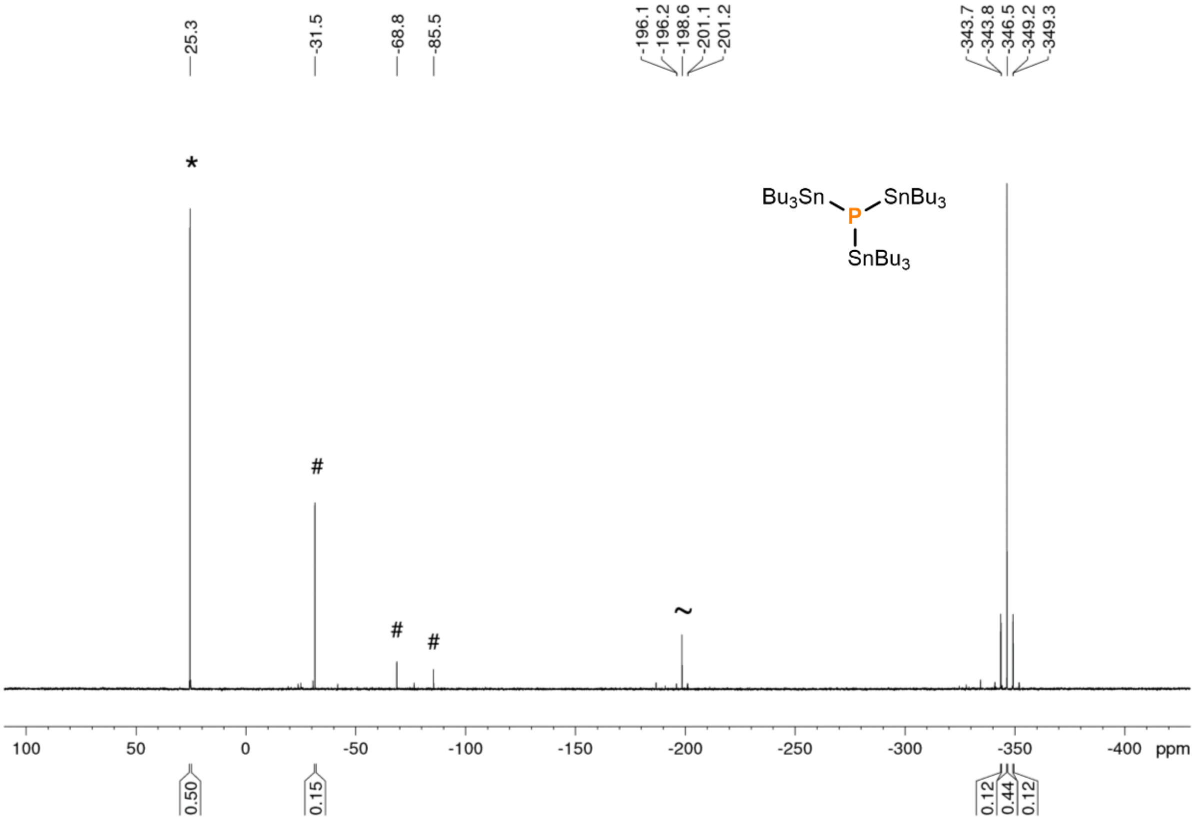

Figure S9. $^{31}P\{^{1}H\}$ NMR spectrum for the photocatalytic functionalization of P_4 using **thioxanthone** (**TXT**) as a photocatalyst (Table S7, Entry 5). * marks the internal standard Ph_3PO (0.02 mmol). ~ marks an unknown Sn-containing side product. # marks unknown side products.

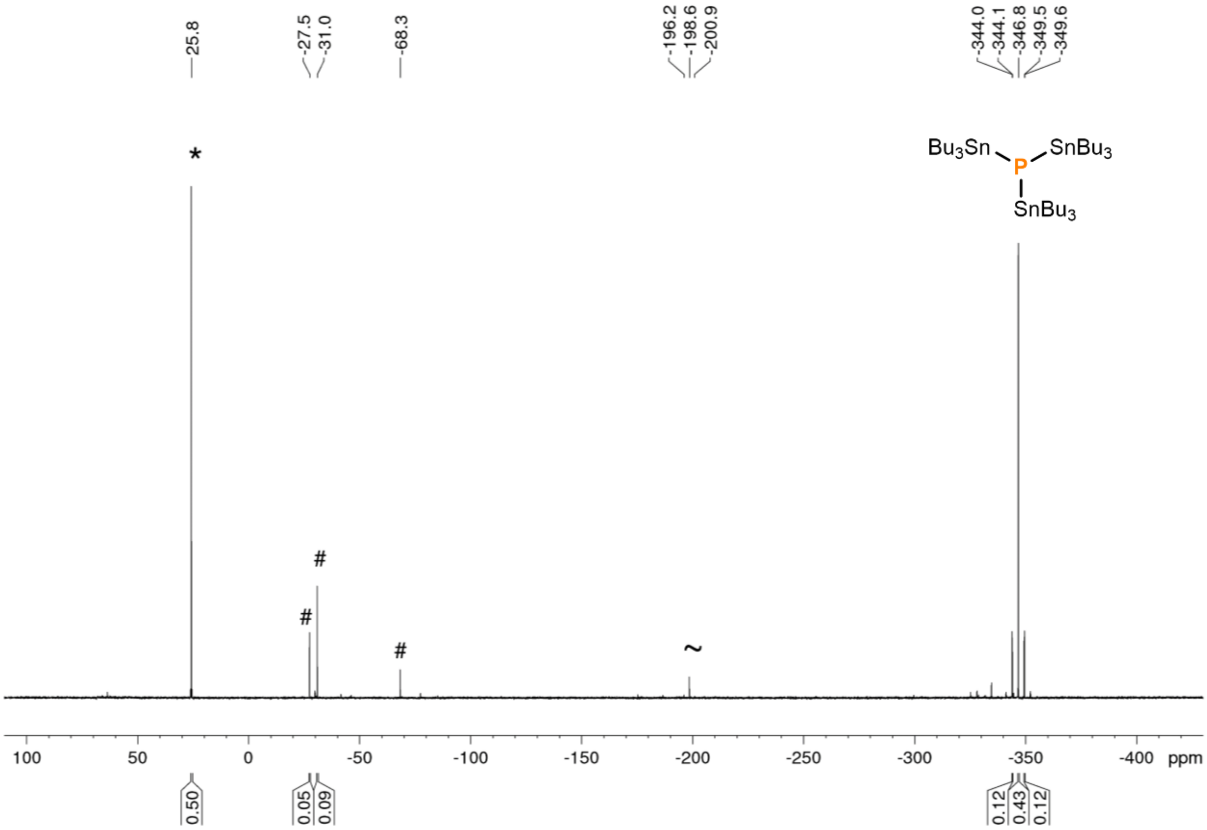

Figure S10. $^{31}P\{^{1}H\}$ NMR spectrum for the photocatalytic functionalization of P_4 using **thioxanthone** (**TXT**) as a photocatalyst (Table S7, Entry 6, **PhH/acetone**). * marks the internal standard Ph_3PO (0.02 mmol). ~ marks an unknown Sn-containing side product. # marks unknown side products.

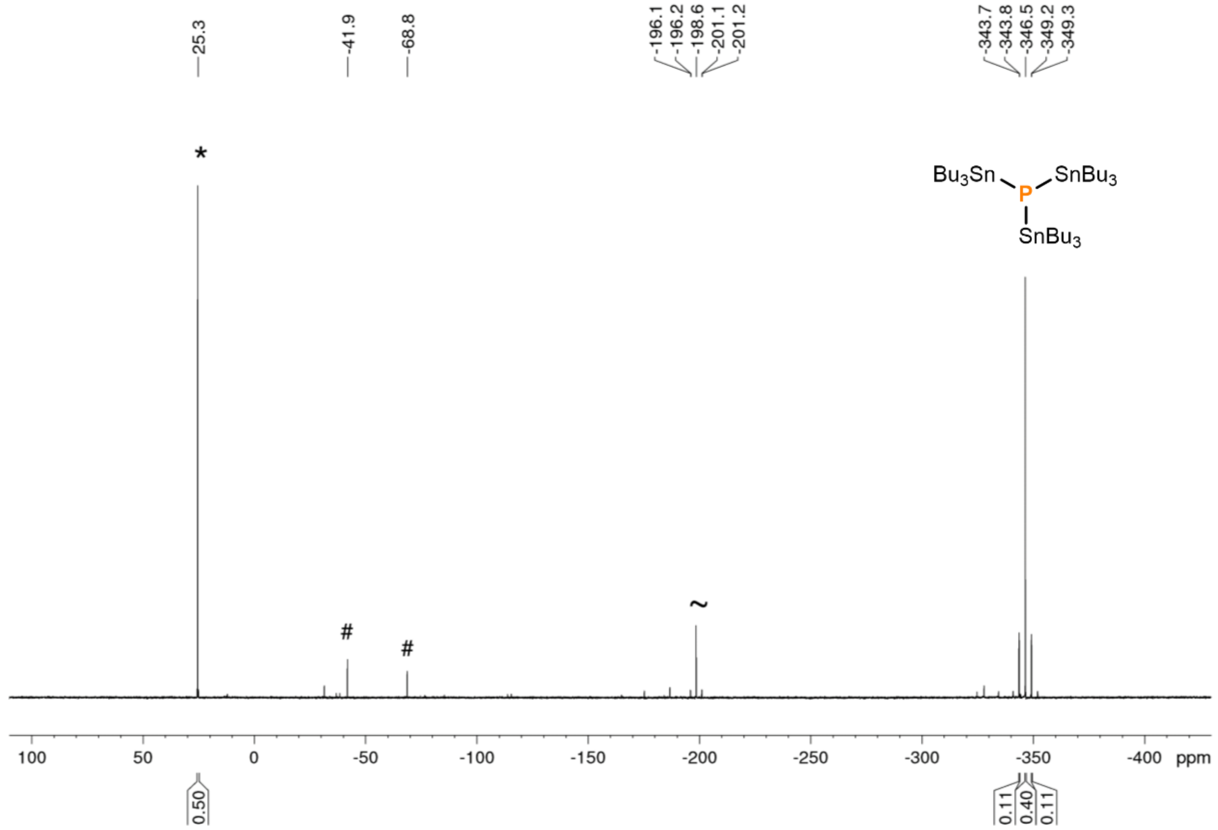

Figure S11. $^{31}P\{^{1}H\}$ NMR spectrum for the photocatalytic functionalization of P_4 using **anthraquinone** (**AQ**) as a photocatalyst (Table S7, Entry 7). ***** marks the internal standard Ph_3PO (0.02 mmol). **~** marks an unknown Sn-containing side product. **#** marks unknown side products.

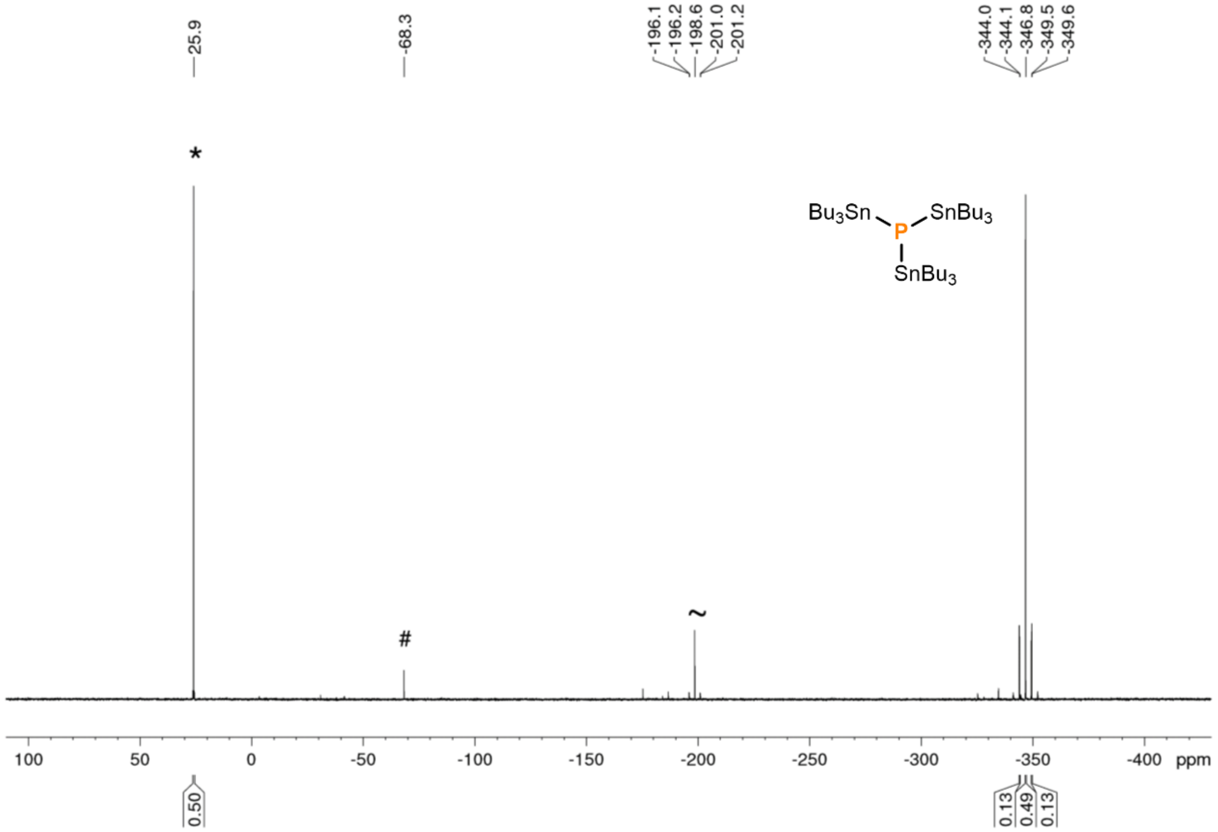

Figure S12. $^{31}P\{^{1}H\}$ NMR spectrum for the photocatalytic functionalization of P_4 using **anthraquinone** (**AQ**) as a photocatalyst (Table S7, Entry 8, **PhH/acetone**). ***** marks the internal standard Ph_3PO (0.02 mmol). **~** marks an unknown Sn-containing side product. **#** marks unknown side products.

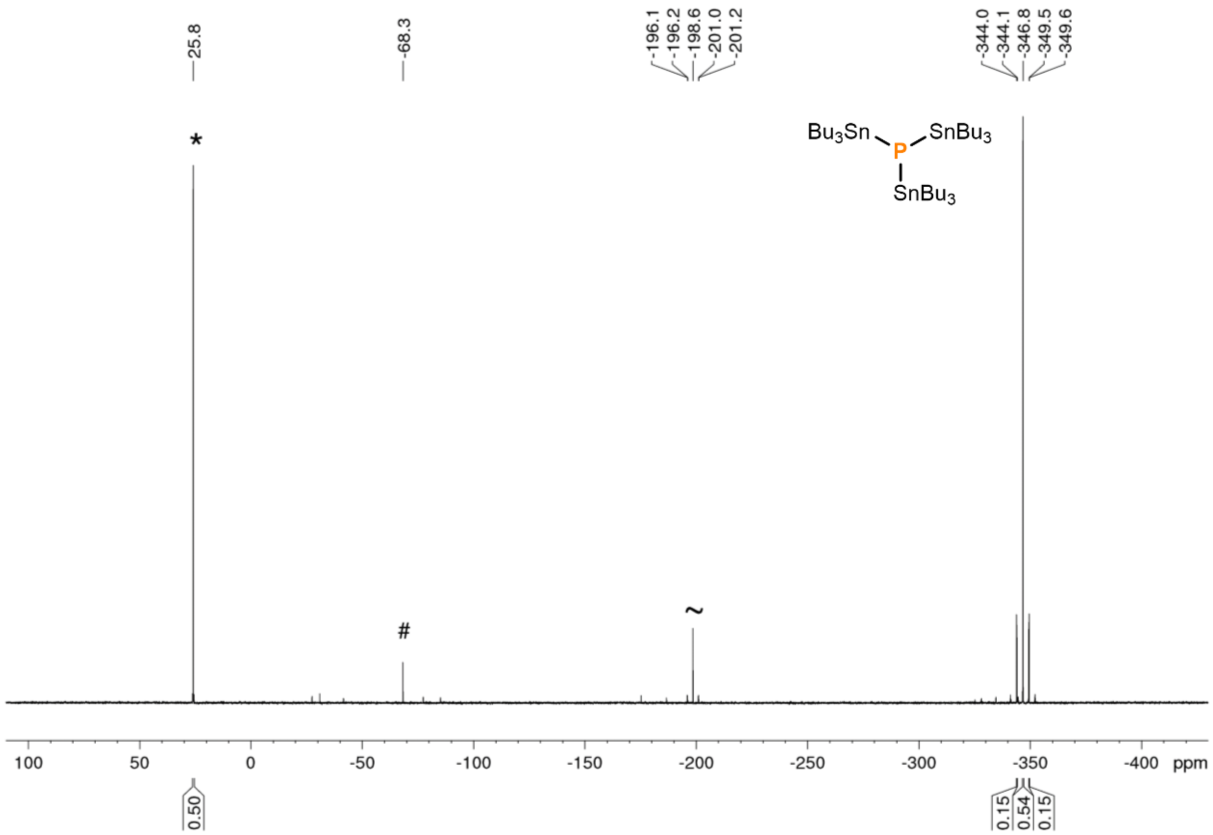

Figure S13. $^{31}P\{^{1}H\}$ NMR spectrum for the photocatalytic functionalization of P_4 using **anthraquinone** (**AQ**) as a photocatalyst (Table S7, Entry 9, **0.5 equiv. anthraquinone** in **PhH/acetone**). ***** marks the internal standard Ph_3PO (0.02 mmol). **~** marks an unknown Sn-containing side product. **#** marks unknown side products.

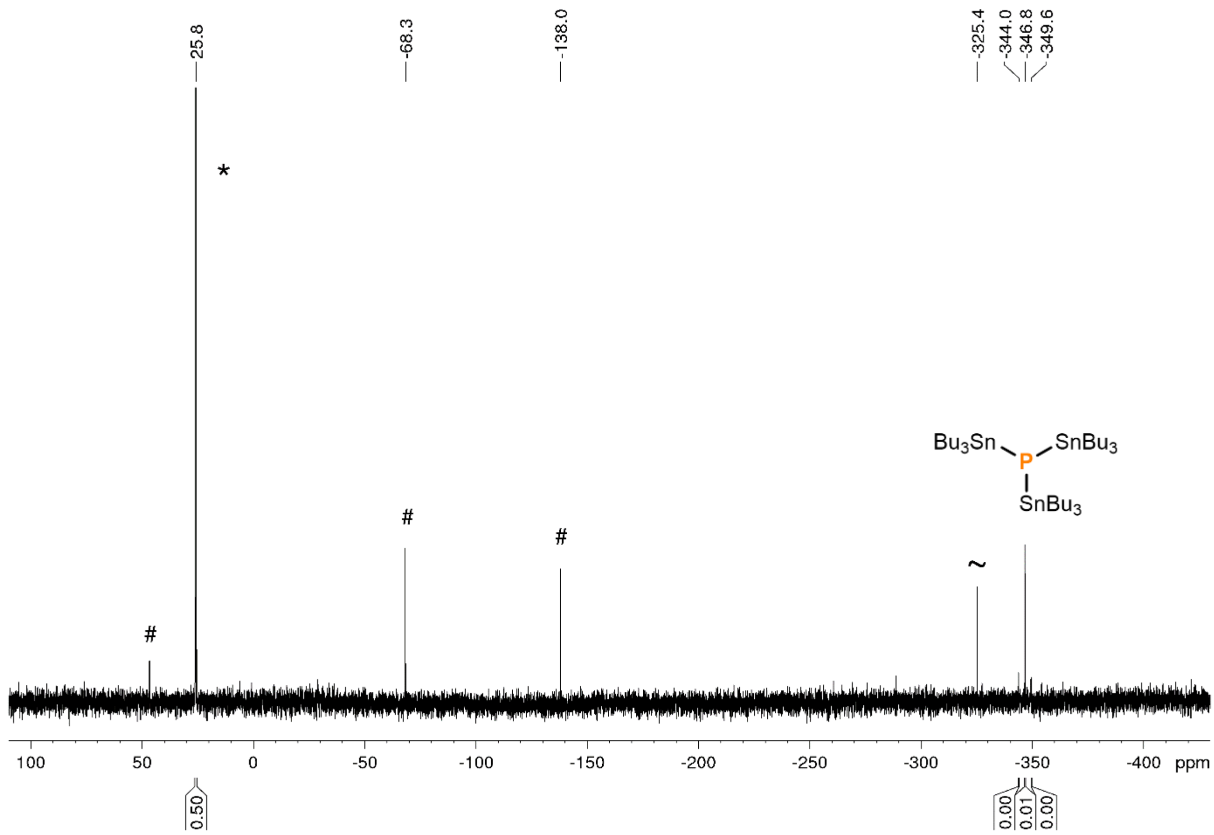

Figure S14. $^{31}P\{^{1}H\}$ NMR spectrum for the photocatalytic functionalization of P_4 using **acetone** as a photocatalyst (Table S7, Entry 10). ***** marks the internal standard Ph_3PO (0.02 mmol). **~** marks an unknown Sn-containing side product. **#** marks unknown side products.

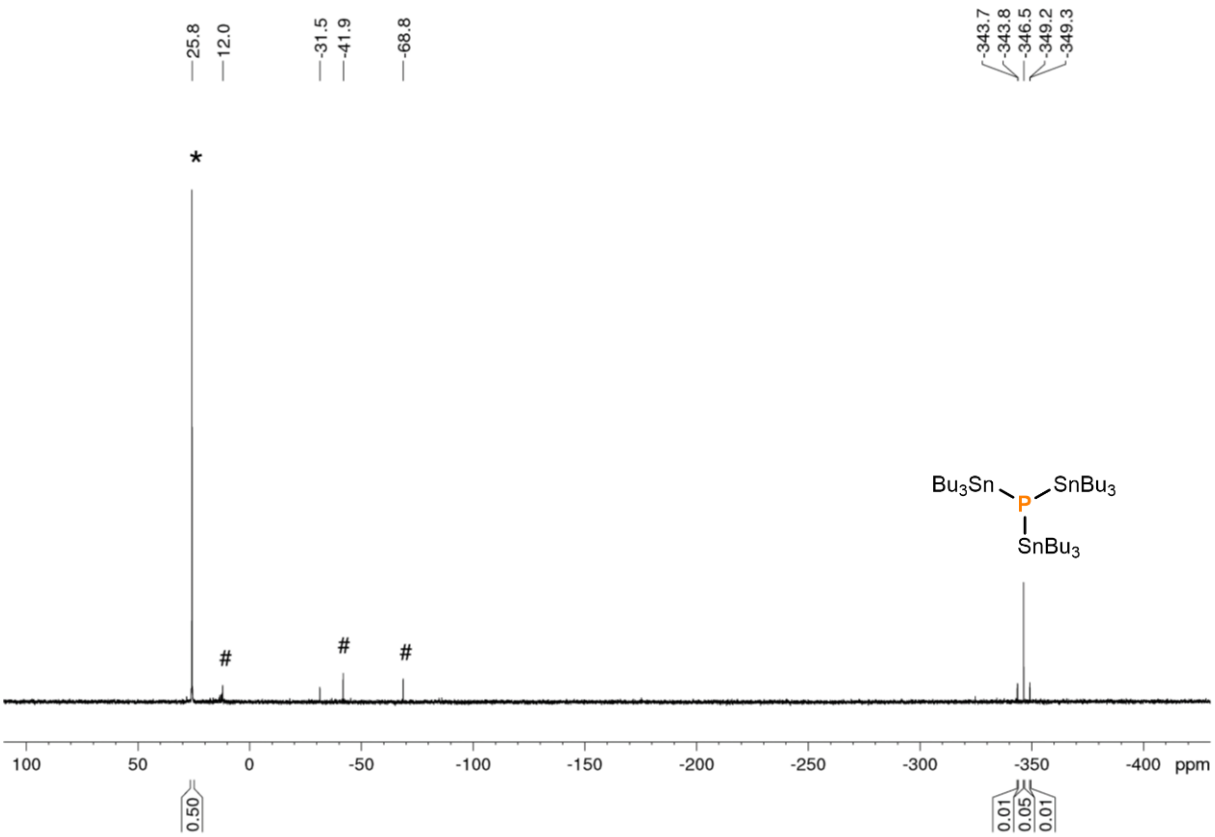

Figure S15. $^{31}P\{^1H\}$ NMR spectrum for the photocatalytic functionalization of P_4 using **DDQ** as a photocatalyst (Table S7, Entry 11). ***** marks the internal standard Ph_3PO (0.02 mmol). **#** marks unknown side products.

S6. General procedure for photocatalytic functionalization of P_4 (0.04 mmol scale) into stannylated phosphine $(Bu_3Sn)_3P$ using anthraquinone

To a 10 mL stoppered tube equipped with a stirring bar were added $(Bu_3Sn)_2$ (101.1 µL, 5 equiv. based on phosphorus atoms, 20 equiv. based on P_4), anthraquinone (**AQ**) (1.0 mg, 0.5 equiv. based on P_4) and P_4 (0.01 mmol, as a stock solution in 71.3 µL benzene) in acetone as solvent (429 µL, in total 0.5 mL PhH/acetone mixture). The tube was sealed, placed in a water-cooled block (to ensure a near-ambient temperature was maintained, Figure S16), and irradiated with UV light (365 nm, 4.3 V, 700 mA, Osram OSLON SSL 80) for 22 h (unless stated otherwise). Ph_3PO (0.02 mmol, stock solution in benzene) was subsequently added to act as an internal standard. The resulting mixture was subjected to $^{31}P\{^{1}H\}$ NMR analysis and showed 83% conversion to the product $(Bu_3Sn)_3P$[1] (Figure S17).

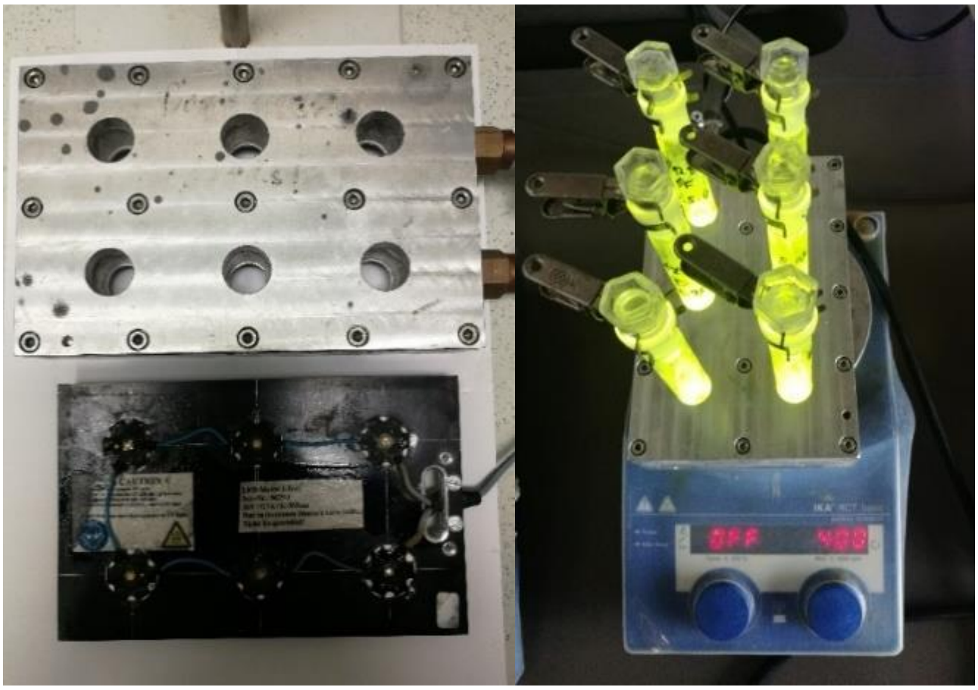

Figure S16. Illustration of the equipment setup used for photocatalytic reactions at 0.04 mmol scale.

Spectroscopic data of $(Bu_3Sn)_3P$:

$^{31}P\{^{1}H\}$ and $^{119}Sn\{^{1}H\}$ NMR data of the photocatalytically generated $(Bu_3Sn)_3P$ were extracted from spectra of the crude reaction mixture (see Figure S17 and S18), and are consistent with previous reports.[0] Note that isolation of $(Bu_3Sn)_3P$ was not pursued due to separation from unreacted $(Bu_3Sn)_2$ being complicated by very similar solubilities as well as the high boiling point of both compounds.

$^{31}P\{^{1}H\}$ NMR (161.98 MHz, 300 K, C_6D_6): $\delta = -346.8$ ppm (s).

$^{119}Sn\{^{1}H\}$ NMR (149.21 MHz, 300 K, C_6D_6): $\delta = 38.0$ ppm (d, $^{1}J(^{31}P\text{-}^{119}Sn) = 912.5$ Hz, $^{2}J(^{119}Sn\text{-}^{117}Sn) = 278.2$ Hz).

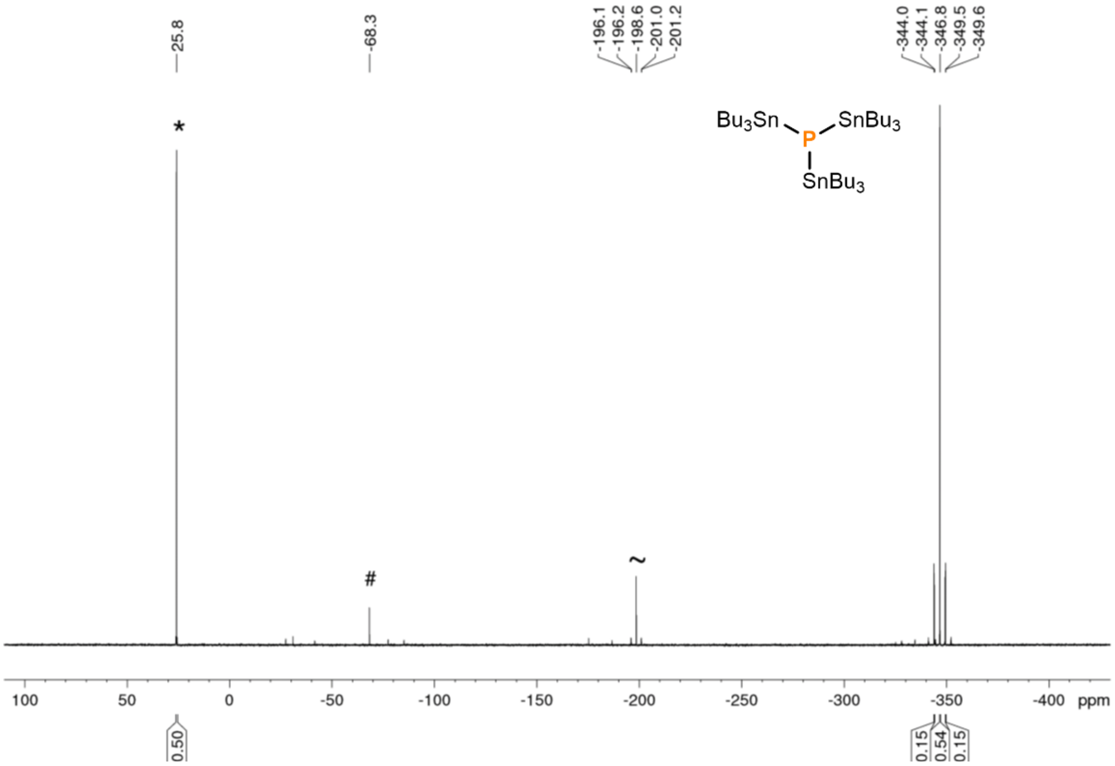

Figure S17. Representative $^{31}P\{^1H\}$ NMR spectrum for the photocatalytic functionalization of P_4 using **anthraquinone** (**AQ**) as a photoinitiator (section S5, Table S8, Entry 1). * marks the internal standard Ph_3PO (0.02 mmol). ~ marks an unknown Sn-containing side product. # marks unknown side products.

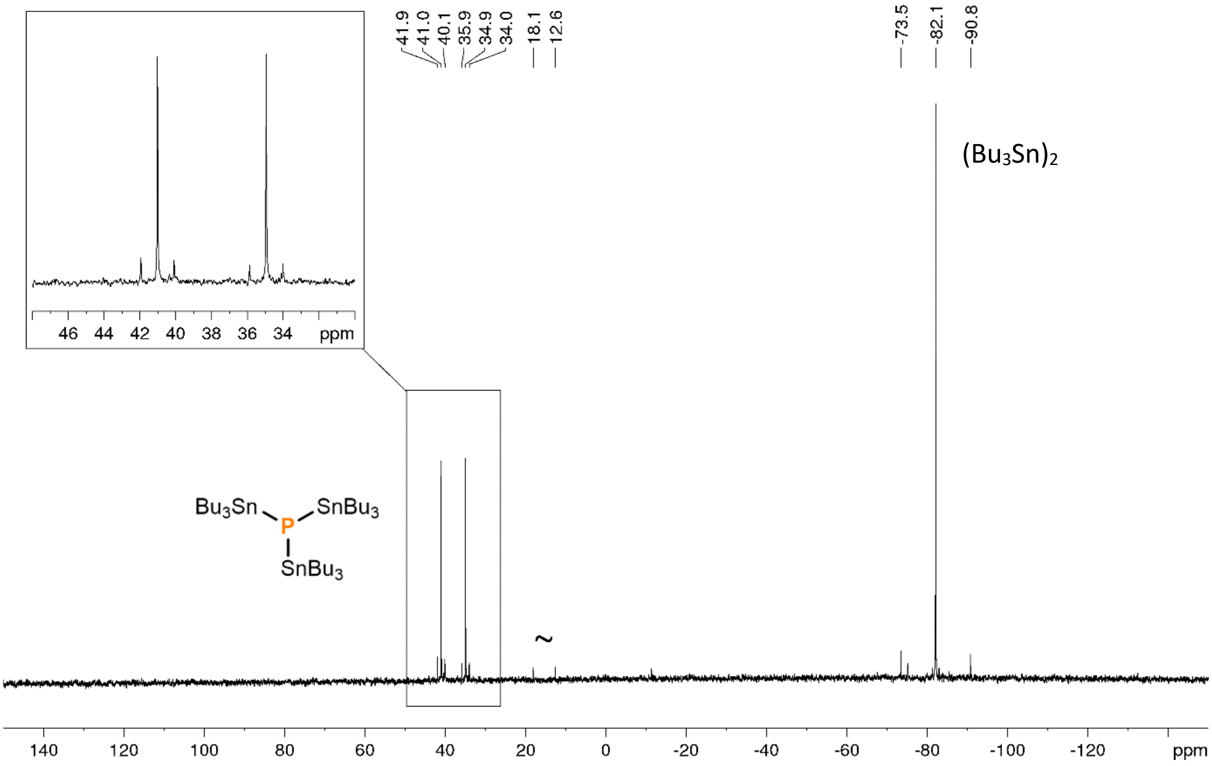

Figure S18. $^{119}Sn\{^1H\}$ NMR spectrum for the photocatalytic functionalization of P_4 using **anthraquinone** (**AQ**) as a photoinitiator (**0.5 equiv. anthraquinone** and **12 equiv.** $(Bu_3Sn)_2$ in **PhH/acetone**). ~ marks an unknown side product (see Figure S15, $^{31}P\{^1H\}$ NMR δ = −198.6 ppm).

S7. Optimization of photocatalytic reaction conditions using anthraquinone

Table S8. Photocatalytic functionalization of P_4 to $(Bu_3Sn)_3P$: screening of **control experiments.**[a]

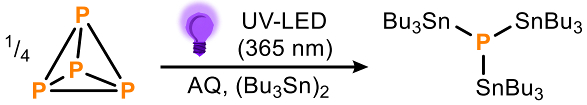

Entry	Conditions	Full conv. of **P_4**?	Conv. to **$(Bu_3Sn)_3P$** / %
1	Standard[a]	✓	83
2	No light	X	0
3	No **AQ**[b]	✓	2
4	No $(Bu_3Sn)_2$	X	0
5	Blue LEDs (455 nm)	X	15
6	Violet/Blue LEDs (420 nm)	✓	76
7	P_{red} instead of P_4	–	0

[a] For the general procedure, see section S6. [b] The reaction without anthraquinone (**AQ**) as a photoinitiator shows small conversion to $(Bu_3Sn)_3P$ presumably because acetone used as a solvent can act as an alternative but less efficient photocatalyst (see section S5, Table S7, Entry 10).

Table S9. Photocatalytic functionalization of P_4 to $(Bu_3Sn)_3P$: screening of different **radiation sources (LEDs).**[a]

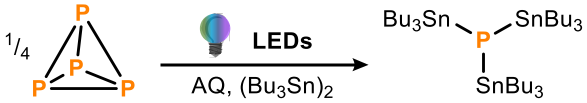

Entry	LEDs	Full conv. of **P_4**?	Conv. to **$(Bu_3Sn)_3P$** / %
**1**	**365 nm**[a] **(UV LED)**	✓	**83**
2	385 nm (UV LED)	✓	66
3	405 nm (violet LED)	✓	21
4	420 nm (violet blue LED)	✓	76
5	455 nm (blue LED)	✓	15
6	528 nm (green LED)	X	0

[a] The general procedure (section S6) was modified to use the LEDs indicated.

Table S10. Photocatalytic functionalization of P_4 to $(Bu_3Sn)_3P$: screening of **solvents**.[a]

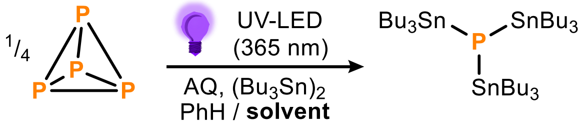

Entry	Solvent	Full conv. of **P_4**?	Conv. to **$(Bu_3Sn)_3P$** / %
1	Benzene[a]	✓	58
**2**	**Acetone**	✓	**83**
3	Acetone[b]	✓	79
4	Acetonitrile	✓	5
5	THF	✓	35
6	Toluene	✓	60
7	Ethanol	✓	3

[a] The general procedure (section S6) was modified to use a solvent mixture (P_4 stock solutions in benzene). [b] 0.22 mL solvent volume.

Table S11. Photocatalytic functionalization of P_4 to $(Bu_3Sn)_3P$: screening of **amounts of anthraquinone.**[a][b]

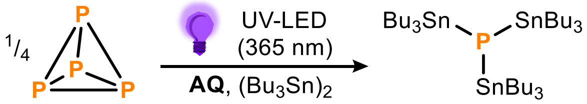

Entry	**AQ** / equiv.[b]	Full conv. of **P_4**?	Conv. to **$(Bu_3Sn)_3P$** / %
1	0.01[c]	✓	4
2	0.1[c]	✓	47[d]
3	0.25	✓	70[e]
**4**	**0.5**	✓	**83**
5	1	✓	73
6	4	✓	45

[a] For the general procedure, see section S6. [b] Listed equivalents are defined per P_4 molecule. [c] For catalytic use of photoinitiator anthraquinone (**AQ**) a stock solution (1 mg **AQ** in 1 mL benzene) was prepared for screening 0.01 equiv. **AQ** (20.8 µL, Entry 1) and 0.1 mmol **AQ** (208 µL, Entry 2). [d] This catalyst loading corresponds to a TON of 28.2. [e] This catalyst loading corresponds to a TON of 16.8.

Table S12. Photocatalytic functionalization of P_4 to $(Bu_3Sn)_3P$: screening of **amounts of $(Bu_3Sn)_2$**.[a][b]

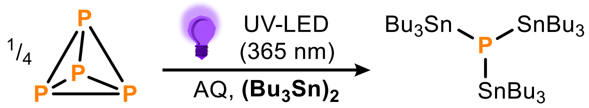

**Entry**	**AQ** / equiv.	**$(Bu_3Sn)_2$** / equiv.[a]	Full conv. of **P_4**?	Conv. to **$(Bu_3Sn)_3P$ / %**
1	1	30	✓	82
2	1	20	✓	75
3	1	12	✓	82
4	1	6	✓	61
5	0.5	30	✓	84
**6**	**0.5**	**20**	✓	**83**
**7**	**0.5**	**12**	✓	**79**
8	0.5	6	✓	64

[a] For the general procedure, see section S6. [b] Listed equivalents are defined per P_4 molecule (0.01 mmol).

Note:

In the final stage of optimization, it was found that reducing the $(Bu_3Sn)_2$ loading from 20 equiv. to 12 equiv. had only a very minor impact on conversion to $(Bu_3Sn)_3P$ (from 83% to 79%). Thus, while the reaction with 20 equiv. formally gave the best conversion, the reaction with 12 equiv. was chosen as being optimal for further elaboration into 'one pot' reactions, as it should reduce the formation of stoichiometric, Sn-containing waste.

S8. Characterization of optimized 0.04 mmol scale reactions using anthraquinone

S8.1 Synthesis of triacylphosphines (R(O)C)$_3$P (R = Ph, Ad, Cy, *t*Bu, *i*Pr, *n*Bu, Me)[1]

To a 10 mL stoppered tube equipped with a stirring bar were added $(Bu_3Sn)_2$ (60.6 µL, 3 equiv. based on phosphorus atoms, 12 equiv. based on P_4), anthraquinone (**AQ**) (1.0 mg, 0.5 equiv. based on P_4) and P_4 (0.01 mmol, as a stock solution in 71.3 µL benzene) in acetone as solvent (429 µL, in total 0.5 mL PhH/acetone mixture). The tube was sealed, placed in a water-cooled block (to ensure a near-ambient temperature was maintained, Figure S14), and irradiated with UV light (365 nm, 4.3 V, 700 mA, Osram OSLON SSL 80) for 22 h. To further functionalize the P_1 intermediate $(Bu_3Sn)_3P$ into triacylphosphines (R(O)C)$_3$P in an 'one-pot' manner, different acyl chlorides RC(O)Cl (0.16 mmol, 16 equiv. based on P_4, 4 equiv. based on phosphorus atoms, with R = Ph, Ad, Cy, *t*Bu, *i*Pr, *n*Bu and Me, see Table S13) were added to the photocatalytic reaction mixture, which each showed a color change from an orange to a yellow solution while stirring overnight.

Table S13. Substrate scope for functionalization of $(Bu_3Sn)_3P$ generated photocatalytically using anthraquinone into **triacyl phosphines**.

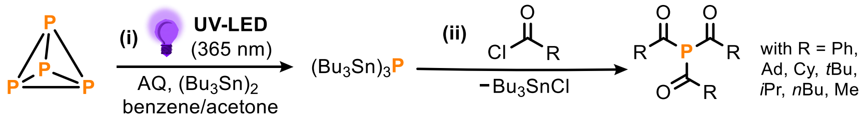

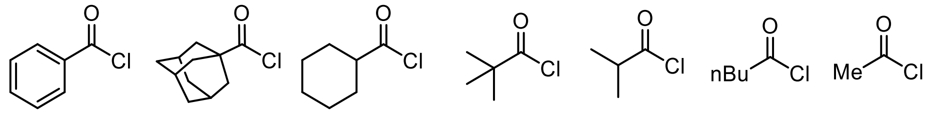

1 2 3 4 5 6 7

Substrate	R	Full conv. of **(Bu$_3$Sn)$_3$P?**	Conv.[a] to **(R(O)C)$_3$P / %**
**1**	R = Phenyl (Ph)	✓	75
**2**	R = Adamantyl (Ad)	✓	40
**3**	R = Cyclohexyl (Cy)	✓	66
**4**	R = *tert*-Butyl (*t*Bu)	✓	64
**5**	R = *iso*-Propyl (*i*Pr)	✓	67
**6**	R = *n*-Butyl (*n*Bu)	✓	54
**7**	R = Methyl (Me)	✓	60

'One-pot' synthesis of triacyl phosphines: (i) For the general procedure of the photocatalytic stannylation of P_4 into $(Bu_3Sn)_3P$ (0.04 mmol scale) see section S6. (ii) Subsequent functionalization of $(Bu_3Sn)_3P$ using acyl chlorides to generate triacyl phosphines (R(O)C)$_3$P. The conversions were determined by $^{31}P\{^1H\}$ NMR experiments (161.98 MHz, 300 K, C_6D_6) (0.02 mmol Ph_3PO as internal standard). [a] The conversions were determined by quantitative $^{31}P\{^1H\}$ NMR ($T1_{IS} < T1_{Prod}$ = 10s, D1 = 40 s, (zgig30, inverse gated decoupled).

S8.1.2 Synthesis and quantification of $(Ph(O)C)_3P$

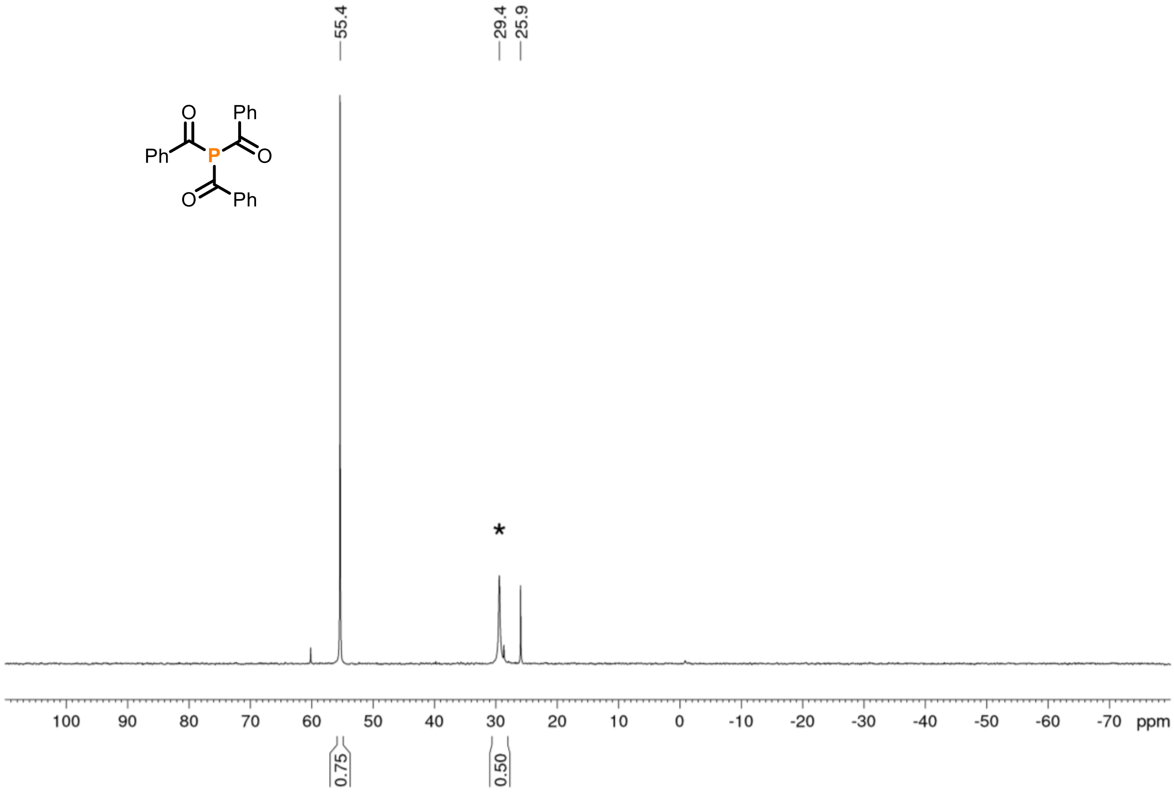

Figure S19. Quantitative $^{31}P\{^1H\}$ NMR spectrum of $(Ph(O)C)_3P$ generated *via* photocatalytic stannylation of P_4 in benzene/acetone followed by acylation with benzoyl chloride PhC(O)Cl (**1**) (Table S13, **12 equiv. $(Bu_3Sn)_2$, $T1_{IS} < T1_{Prod}$ = 10s, D1 = 40 s, zgig30, inverse gated decoupled, LB = 10**). * marks the internal standard Ph_3PO (0.02 mmol).

S8.1.3. Synthesis and quantification of $(Ad(O)C)_3P$

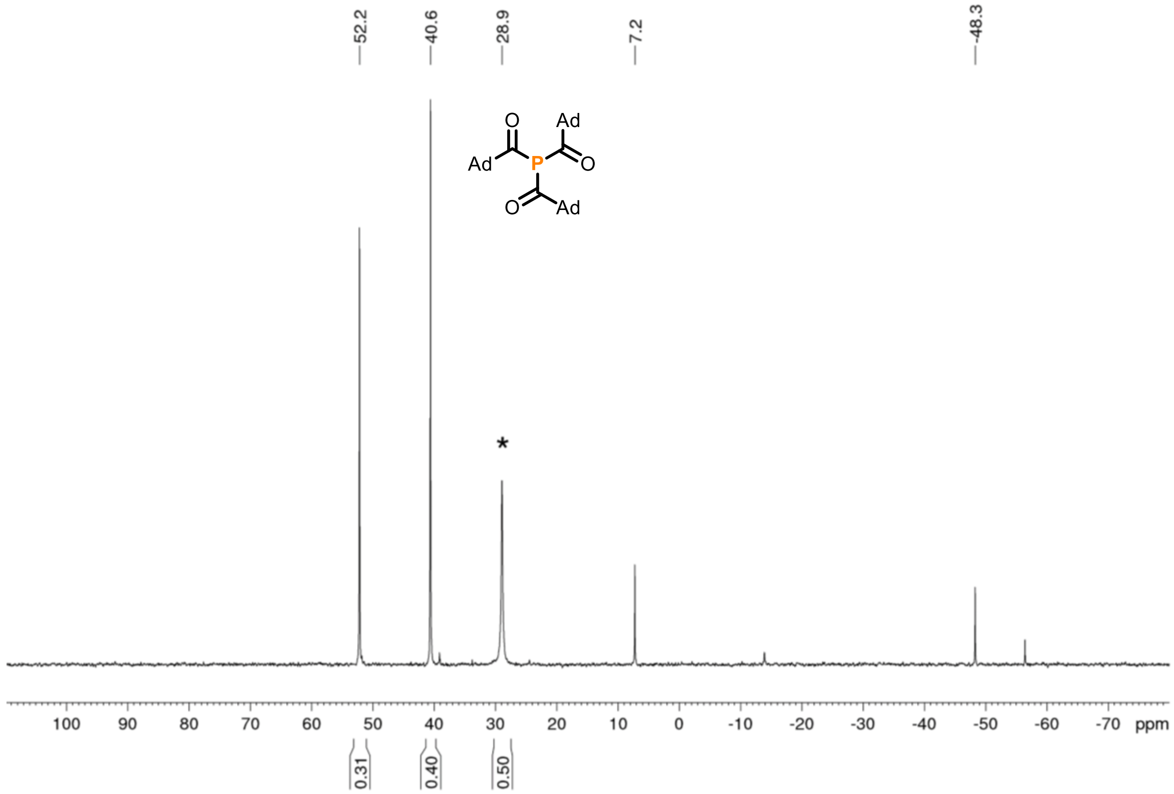

Figure S20. Quantitative $^{31}P\{^1H\}$ NMR spectrum of $(Ad(O)C)_3P$ generated *via* photocatalytic stannylation of P_4 in benzene/acetone followed by acylation with 1-adamantanecarbonyl chloride AdC(O)Cl (**2**) (Table S13, **12 equiv. $(Bu_3Sn)_2$, $T1_{IS} < T1_{Prod}$ = 10s, D1 = 40 s, zgig30, inverse gated decoupled, LB = 10**) * marks the internal standard Ph_3PO (0.02 mmol).

S8.1.4. Synthesis and quantification of (Cy(O)C)$_3$P

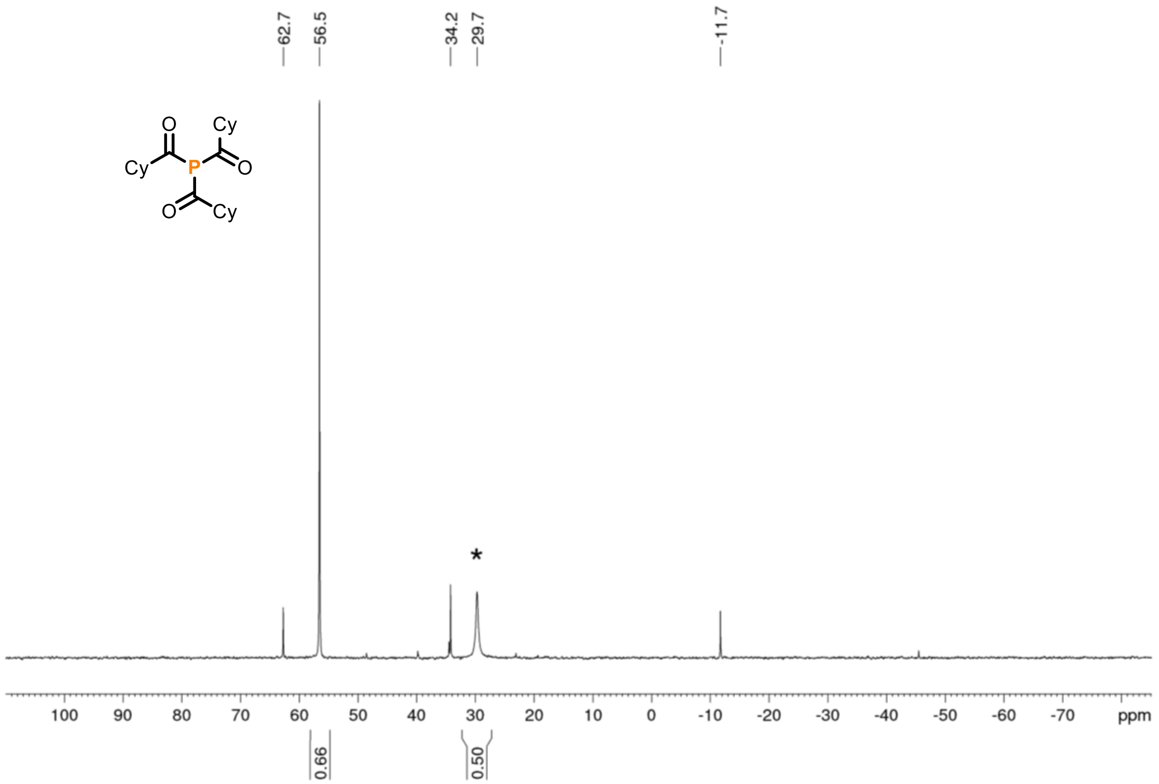

Figure S21. Quantitative $^{31}P\{^1H\}$ NMR spectrum of (Cy(O)C)$_3$P generated *via* photocatalytic stannylation of P_4 in benzene/acetone followed by acylation with cyclohexanecarbonyl chloride CyC(O)Cl (**3**) (Table S13, **12 equiv. (Bu$_3$Sn)$_2$, T1$_{IS}$ < T1$_{Prod}$= 10s, D1 = 40 s, zgig30, inverse gated decoupled, LB = 10**). * marks the internal standard Ph$_3$PO (0.02 mmol).

S8.1.5. Synthesis and quantification of (*t*Bu(O)C)$_3$P

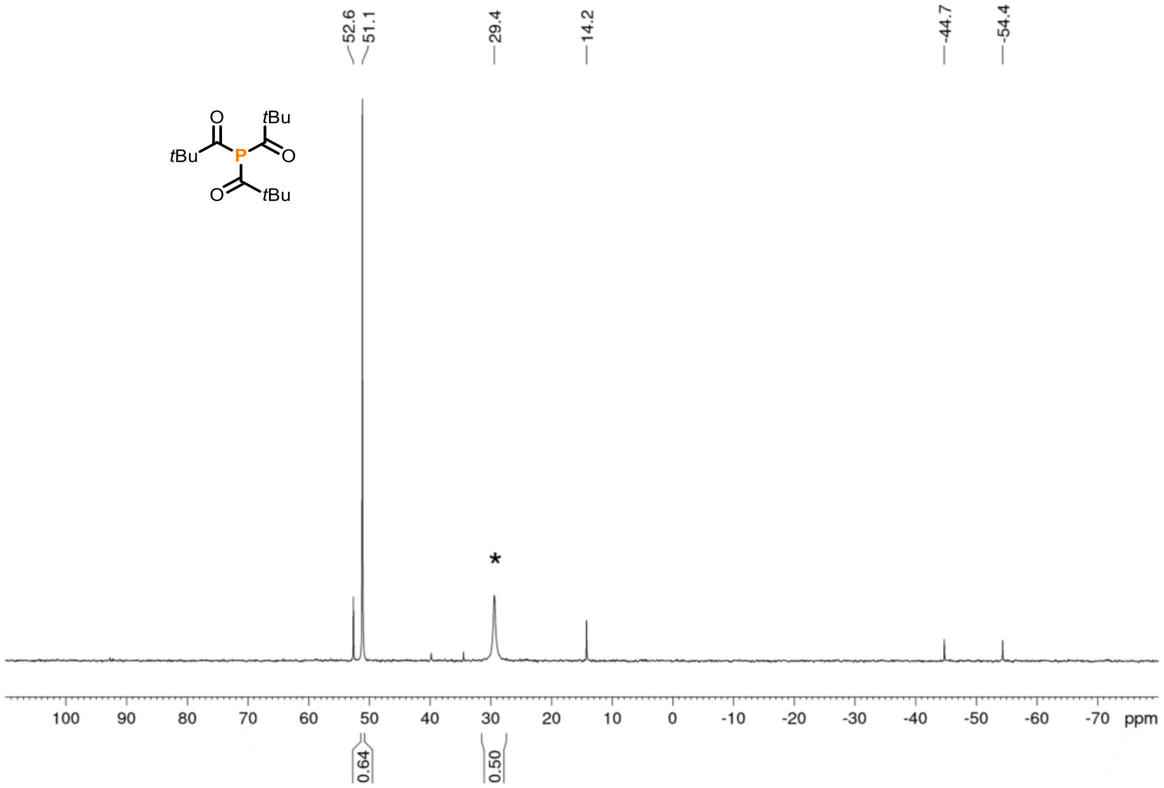

Figure S22. Quantitative $^{31}P\{^1H\}$ NMR spectrum of (*t*Bu(O)C)$_3$P generated *via* photocatalytic stannylation of P_4 in benzene/acetone followed by acylation with pivaloyl chloride *t*BuC(O)Cl (**4**). (Table S13, **12 equiv. (Bu$_3$Sn)$_2$, T1$_{IS}$ < T1$_{Prod}$ = 10s, D1 = 40 s, zgig30, inverse gated decoupled, LB = 10**) * marks the internal standard Ph$_3$PO (0.02 mmol).

S8.1.6. Synthesis and quantification of (*i*Pr(O)C)$_3$P

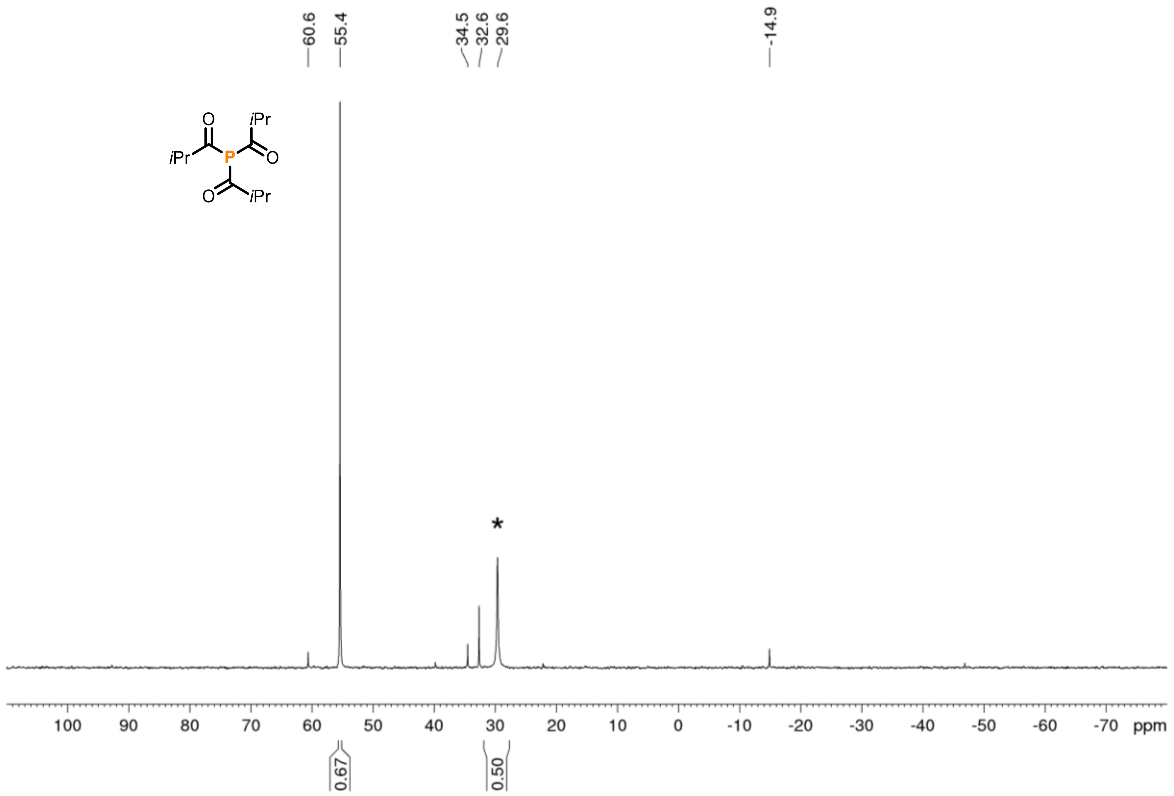

Figure S23. Quantitative $^{31}P\{^{1}H\}$ NMR spectrum of (*i*Pr(O)C)$_3$P generated *via* photocatalytic stannylation of P_4 in benzene/acetone followed by acylation with isobutyryl chloride *i*PrC(O)Cl (**5**). (Table S13, **12 equiv. (Bu$_3$Sn)$_2$, T1$_{IS}$ < T1$_{Prod}$ = 10s, D1 = 40 s, zgig30, inverse gated decoupled, LB = 10**) * marks the internal standard Ph_3PO (0.02 mmol).

S8.1.7. Synthesis and quantification of (*n*Bu(O)C)$_3$P

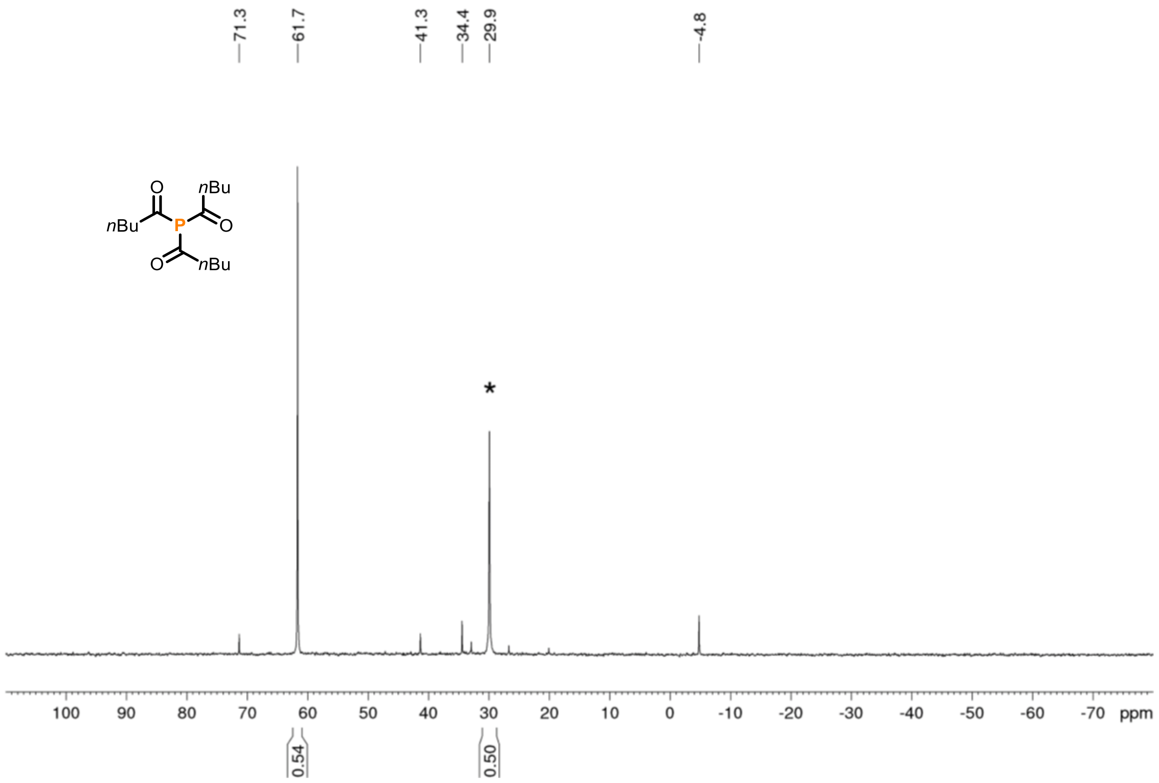

Figure S24. Quantitative $^{31}P\{^{1}H\}$ NMR spectrum of (*n*Bu(O)C)$_3$P generated *via* photocatalytic stannylation of P_4 in benzene/acetone followed by acylation with valeroyl chloride *n*BuC(O)Cl (**6**). (Table S13, **12 equiv. (Bu$_3$Sn)$_2$, T1$_{IS}$ < T1$_{Prod}$ = 10s, D1 = 40 s, zgig30, inverse gated decoupled, LB = 10**). * marks the internal standard Ph_3PO (0.02 mmol).

S8.1.8. Synthesis and quantification of $(Me(O)C)_3P$

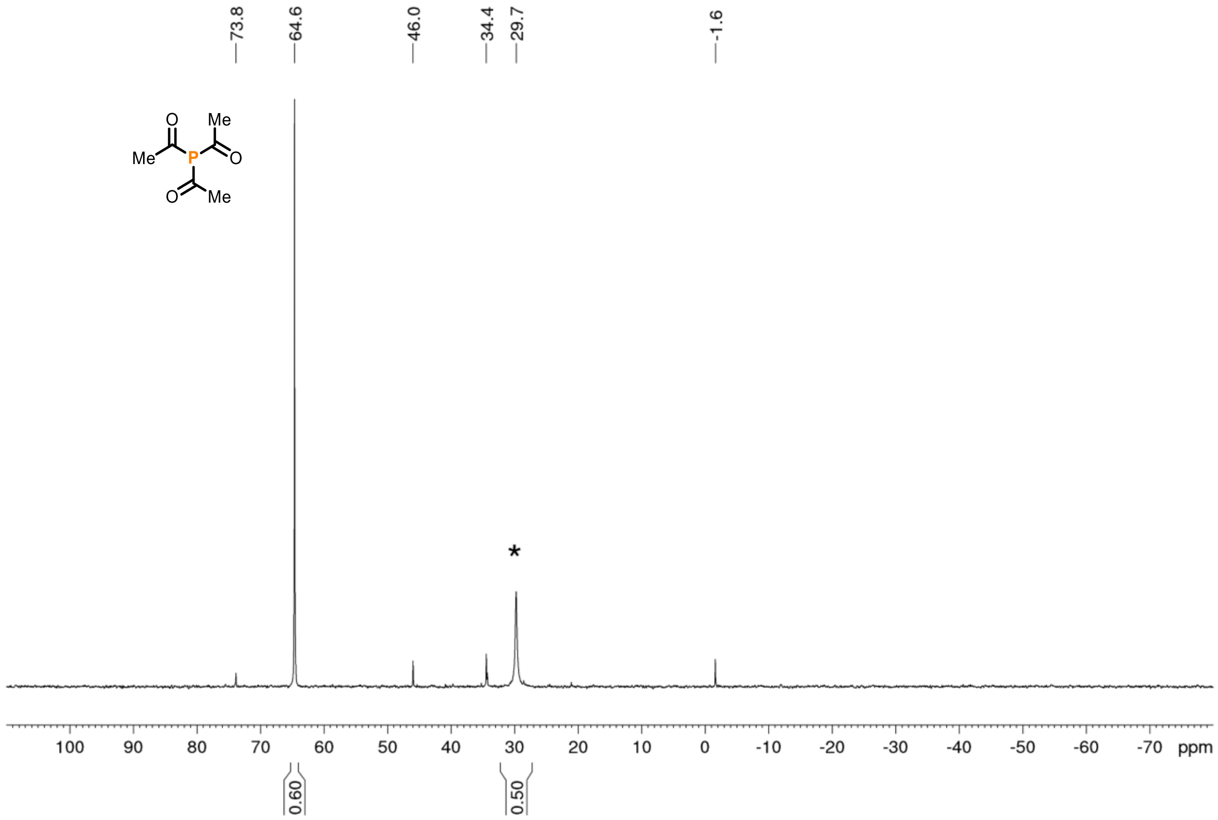

Figure S25. Quantitative $^{31}P\{^{1}H\}$ NMR spectrum of $(Me(O)C)_3P$ generated *via* photocatalytic stannylation of P_4 in benzene/acetone followed by acylation with acetyl chloride MeC(O)Cl (**7**). (Table S13, **12 equiv.** $\mathbf{(Bu_3Sn)_2}$, $\mathbf{T1_{IS} < T1_{Prod}}$ **= 10s, D1 = 40 s, zgig30, inverse gated decoupled, LB = 10**). * marks the internal standard Ph_3PO (0.02 mmol).

S8.2 Synthesis of the phosphonium salts $[R_4P]Br$[0]

To a 10 mL stoppered tube equipped with a stirring bar were added $(Bu_3Sn)_2$ (60.6 µL, 3 equiv. based on phosphorus atoms, 12 equiv. based on P_4), anthraquinone (**AQ**) (1.0 mg, 0.5 equiv. based on P_4) and P_4 (0.01 mmol, as a stock solution in 71.3 µL benzene) in acetone as solvent (429 µL, in total 0.5 mL PhH/acetone mixture). The tube was sealed, placed in a water-cooled block (to ensure a near-ambient temperature was maintained, Figure S14), and irradiated with UV light (365 nm, 4.3 V, 700 mA, Osram OSLON SSL 80) for 22 h. After photocatalytic generation of the stannylated phosphine $(Bu_3Sn)_3P$ directly from P_4 this P_1 intermediate was further converted into phosphonium salts in an 'one-pot' manner. Alkyl bromides such as benzyl bromide or ethyl bromide (0.2 mmol, 20 equiv. based on P_4, 5 equiv. based on phosphorus atoms) were added to the photocatalytic reaction mixture. While heating the reaction mixture (for $[Bn_4P]Br$: overnight at 60 °C, for $[Et_4P]Br$: 2d at 80 °C) a color change from an orange to a yellow solution was obtained. Ph_3PO (0.02 mmol, stock solution in benzene) was subsequently added to act as an internal standard. The resulting mixture was subjected to $^{31}P\{^1H\}$ NMR analysis (Figure S26 and S27).

Table S14. Substrates for functionalization of photocatalytic generated $(Bu_3Sn)_3P$ into **phosphonium salts.**

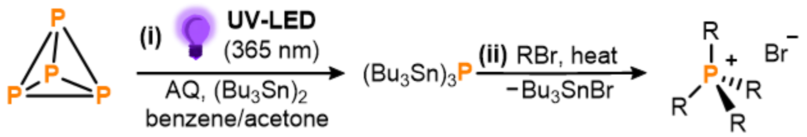

Entry	Substrate	Full conv. of $(Bu_3Sn)_3P$?	Conv. to $[R_4P]^+$ / %
**1**	Benzyl bromide (BnBr)	✓	69%
**2**	Ethyl bromide (EtBr)	✓	46%

One-pot' synthesis of phosphonium salts: (i) For the general procedure of the photocatalytic stannylation of P_4 into $(Bu_3Sn)_3P$ (0.04 mmol scale) see section S6. (ii) Subsequent functionalization of $(Bu_3Sn)_3P$ using alkyl bromides (RBr) to generate phosphonium salts $[R_4P]Br$.

S8.2.1 Synthesis and quantification of [Bn_4P]Br

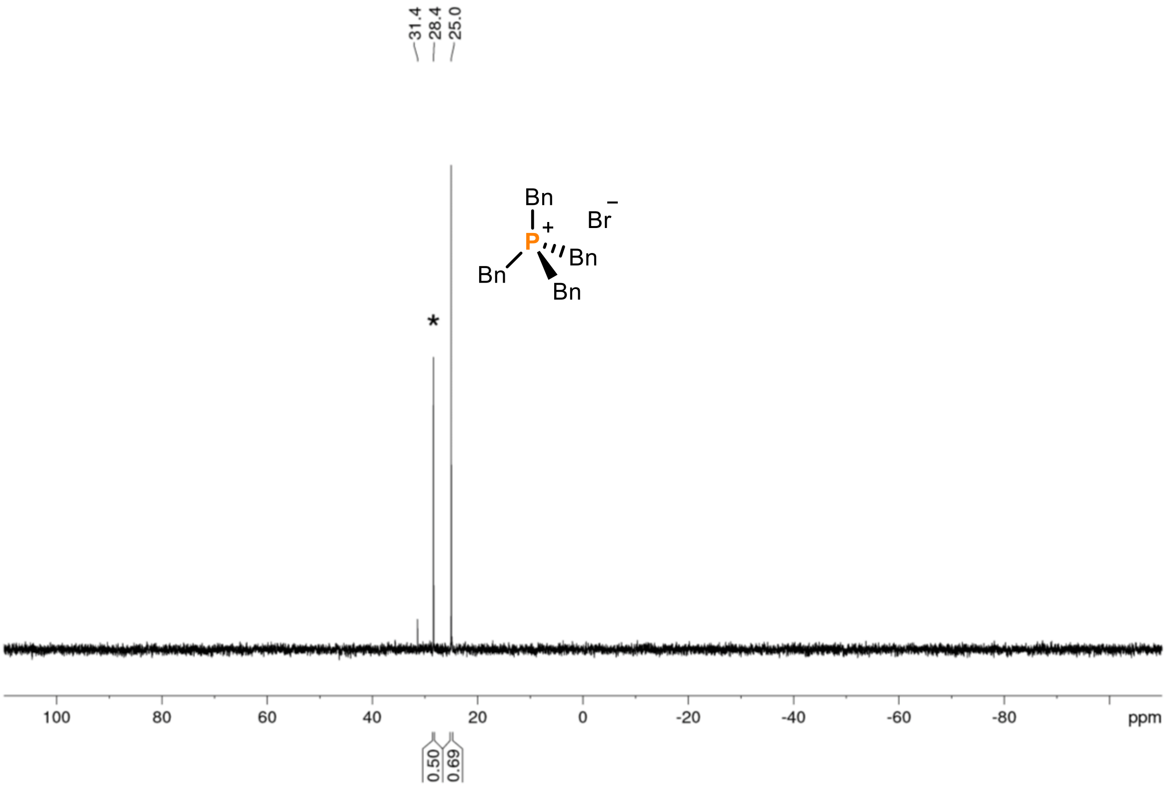

Figure S26. Quantitative $^{31}P\{^1H\}$ NMR (zgig) spectrum of [Bn_4P]Br generated *via* photocatalytic stannylation of P_4 in benzene/acetone followed by alkylation with benzyl bromide (BnBr, Table S14). * marks the internal standard Ph_3PO (0.02 mmol).

S8.2.2 Synthesis and quantification of [Et_4P]Br

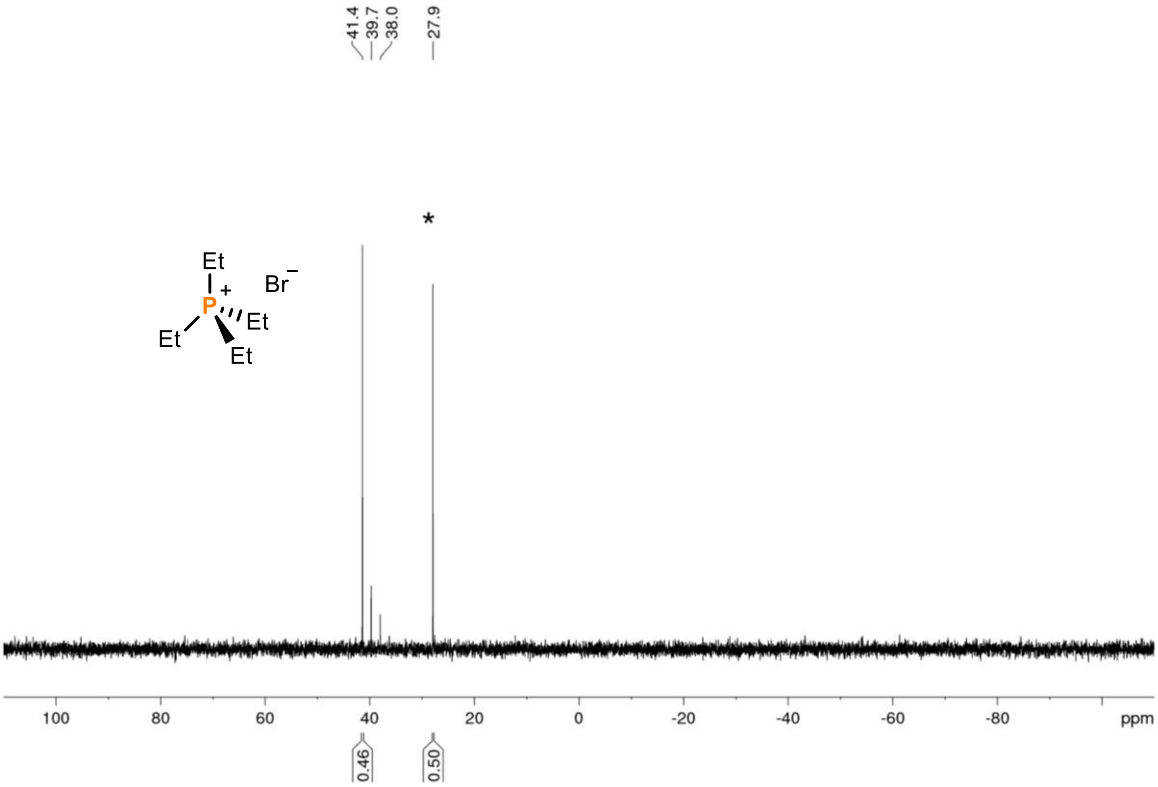

Figure S27. Quantitative $^{31}P\{^1H\}$ NMR (zgig) spectrum of [Et_4P]Br generated *via* photocatalytic stannylation of P_4 in benzene/acetone followed by alkylation with benzyl bromide (EtBr, Table S14). * marks the internal standard Ph_3PO (0.02 mmol).

S8.3 Synthesis of THP, THPO and THPC

S8.3.1 Synthesis of THP and oxidation to THPO

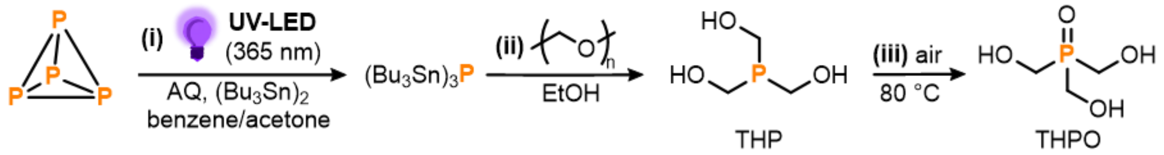

To a 10 mL stoppered tube equipped with a stirring bar were added $(Bu_3Sn)_2$ (60.6 µL, 3 equiv. based on phosphorus atoms, 12 equiv. based on P_4), anthraquinone (**AQ**) (1.0 mg, 0.5 equiv. based on P_4) and P_4 (0.01 mmol, as a stock solution in 71.3 µL benzene) in acetone as solvent (429 µL, in total 0.5 mL PhH/acetone mixture). The tube was sealed, placed in a water-cooled block (to ensure a near-ambient temperature was maintained, Figure S16), and irradiated with UV light (365 nm, 4.3 V, 700 mA, Osram OSLON SSL 80) for 22 h. The photocatalytically generated P_1 intermediate $(Bu_3Sn)_3P$ was further functionalized by removal of volatiles under vacuum and addition of EtOH (1 mL) and paraformaldehyde (3.6 mg, 0.12 mmol, 12 equiv. based on P_4, 3 equiv. based on phosphorus atoms). The resulting suspension was stirred at room temperature for 16 h. Ph_3PO (0.02 mmol, stock solution in benzene) was subsequently added to act as an internal standard. The resulting mixture was subjected to $^{31}P\{^1H\}$ NMR analysis and showed 48% conversion of THP (Figure S28).

Afterwards the 'one-pot' reaction mixture was stirred at 80 °C under air for 16 h to convert the initially formed product THP into its oxidized form THPO ($^{31}P\{^1H\}$ NMR analysis: 38% conversion to THPO, see Figure S29).

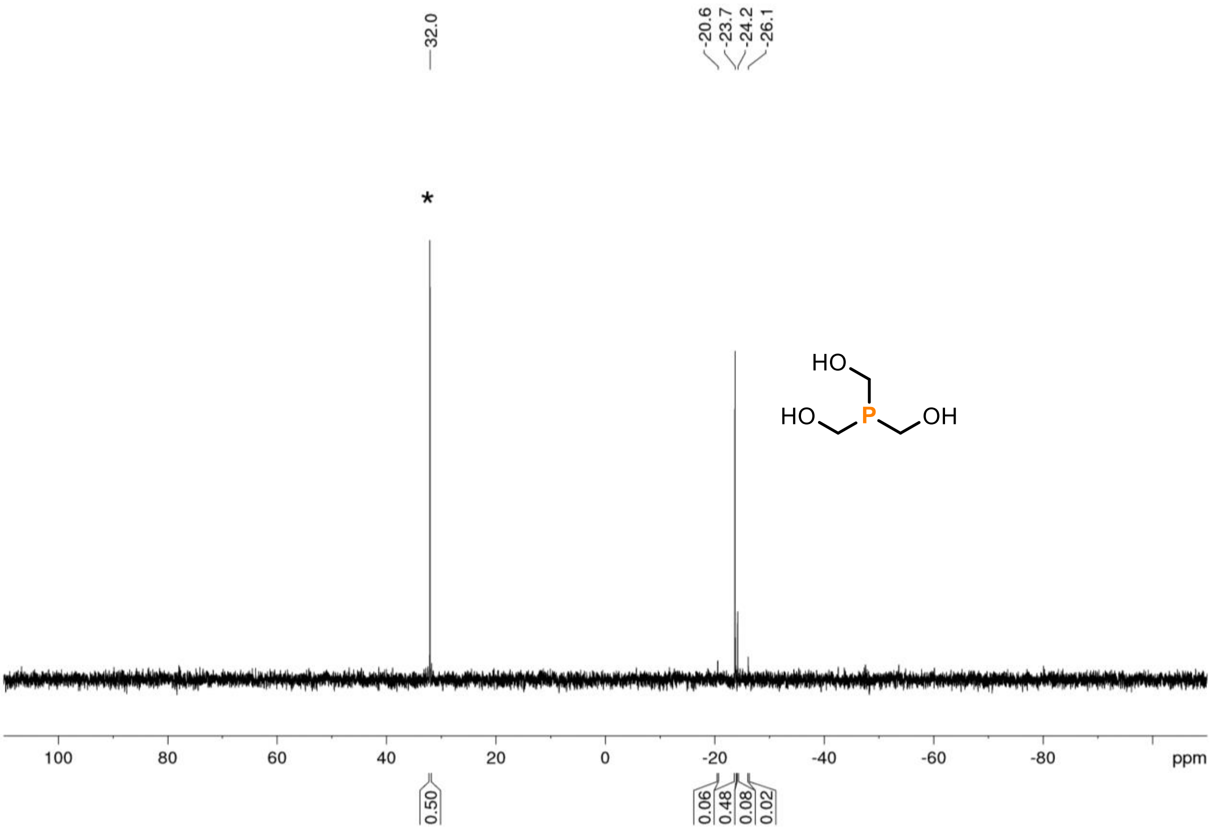

Figure S28. Quantitative $^{31}P\{^1H\}$ NMR (zgig) spectrum of THP generated *via* photocatalytic stannylation of P_4 in benzene/acetone followed by addition of paraformaldehyde in EtOH. * marks the internal standard Ph_3PO (0.02 mmol).

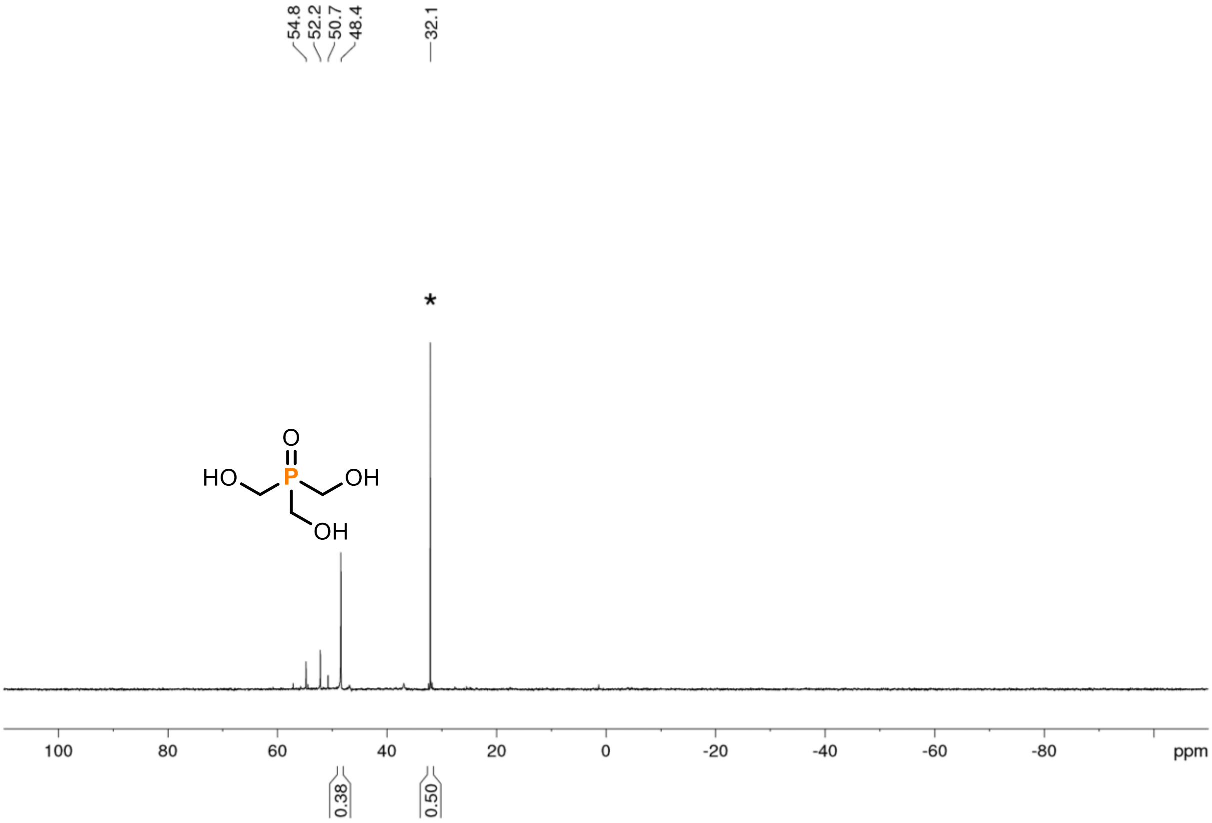

Figure S29. Quantitative $^{31}P\{^1H\}$ NMR spectrum of THPO generated *via* photocatalytic stannylation of P_4 in benzene/acetone followed by addition of paraformaldehyde in EtOH and subsequent oxidation in air. * marks the internal standard Ph_3PO (0.02 mmol).

S8.3.2 Synthesis of THPC

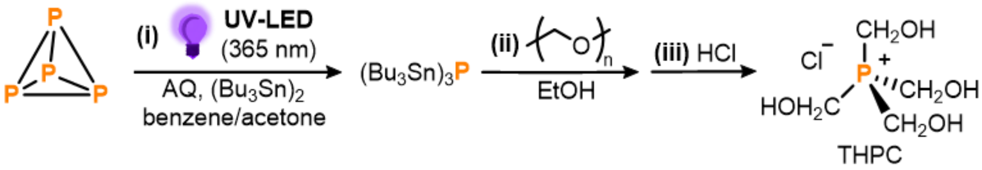

To a 10 mL stoppered tube equipped with a stirring bar were added $(Bu_3Sn)_2$ (60.6 µL, 3 equiv. based on phosphorus atoms, 12 equiv. based on P_4), anthraquinone (**AQ**) (1.0 mg, 0.5 equiv. based on P_4) and P_4 (0.01 mmol, as a stock solution in 71.3 µL benzene) in acetone as solvent (429 µL, in total 0.5 mL PhH/acetone mixture). The tube was sealed, placed in a water-cooled block (to ensure a near-ambient temperature was maintained, Figure S16), and irradiated with UV light (365 nm, 4.3 V, 700 mA, Osram OSLON SSL 80) for 22 h. The photocatalytically generated P_1 intermediate $(Bu_3Sn)_3P$ was further functionalized by removal of volatiles under vacuum and addition of EtOH (1 mL) and paraformaldehyde (15mg, 0.5 mmol, 20 equiv. based on P_4, 5 equiv. based on phosphorus atoms). The resulting suspension was stirred at room temperature for 16 h. The mixture was frozen in a liquid-nitrogen bath, and HCl (0.4 M in 1,4-dioxane, 1 ml, 0.4 mmol, 40 equiv. based on P_4, 10 equiv. based on phosphorus atoms) was added. After thawing, the yellowish reaction mixture was stirred at room temperature for 2 h. Ph_3PO (0.02 mmol, stock solution in benzene) was subsequently added to act as an internal standard. The resulting mixture was subjected to $^{31}P\{^1H\}$ NMR analysis and showed 53% conversion of THPC (Figure S30).

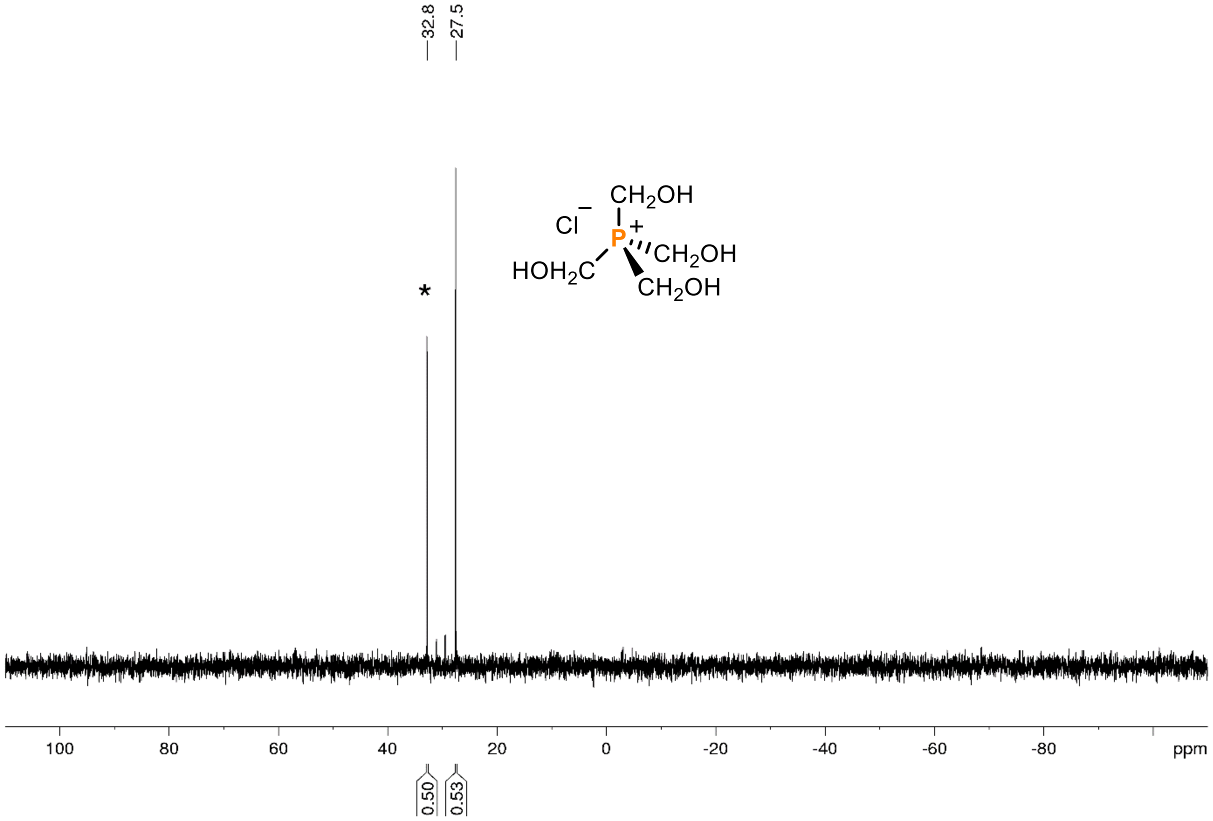

Figure S30. Quantitative $^{31}P\{^{1}H\}$ NMR (zgig) spectrum of THPC generated *via* photocatalytic stannylation of P_4 in benzene/acetone followed by reaction with paraformaldehyde in EtOH and subsequent HCl work-up. * marks the internal standard Ph_3PO (0.02 mmol).

S9. Reactions at 0.8 mmol scale using anthraquinone as a photoinitiator

S9.1 Photocatalytic synthesis of stannylated phosphine $(Bu_3Sn)_3P$[0]

To a 100 mL stoppered tube equipped with a stirring bar were added $(Bu_3Sn)_2$ (1.212 mL, 2.4 mmol, 3 equiv. based on phosphorus atoms, 12 equiv. based on P_4), anthraquinone (**AQ**) (20.8 mg, 0.1 mmol, 0.5 equiv. based on P_4) and P_4 (0.2 mmol, as a stock solution in 1.426 mL benzene) in acetone as solvent (2.574 mL, in total 4 mL PhH/acetone mixture). The tube was sealed, placed in a water-cooled block (to ensure a near-ambient temperature was maintained, Figure S31), and irradiated with UV light (365 nm, 14 V, 700 mA, Osram OSLON SSL 80) for 48 h. $^{31}P\{^1H\}$ NMR spectroscopy (Ph_3PO as internal standard) showed 64% conversion to the stannylated phosphine $(Bu_3Sn)_3P$ (see Figure S32).

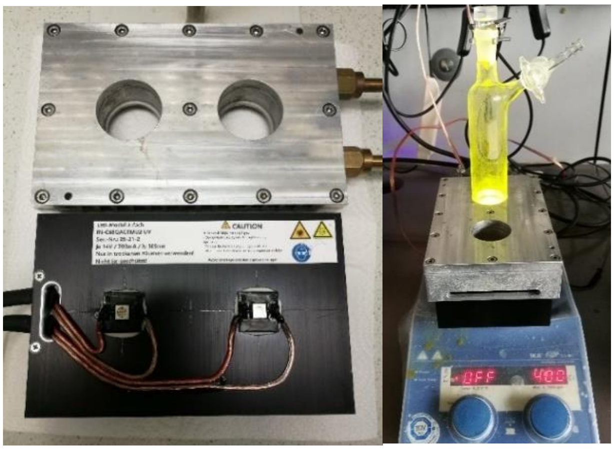

Figure 31. Illustration of the equipment setup used for photocatalytic reactions at 0.8 mmol scale.

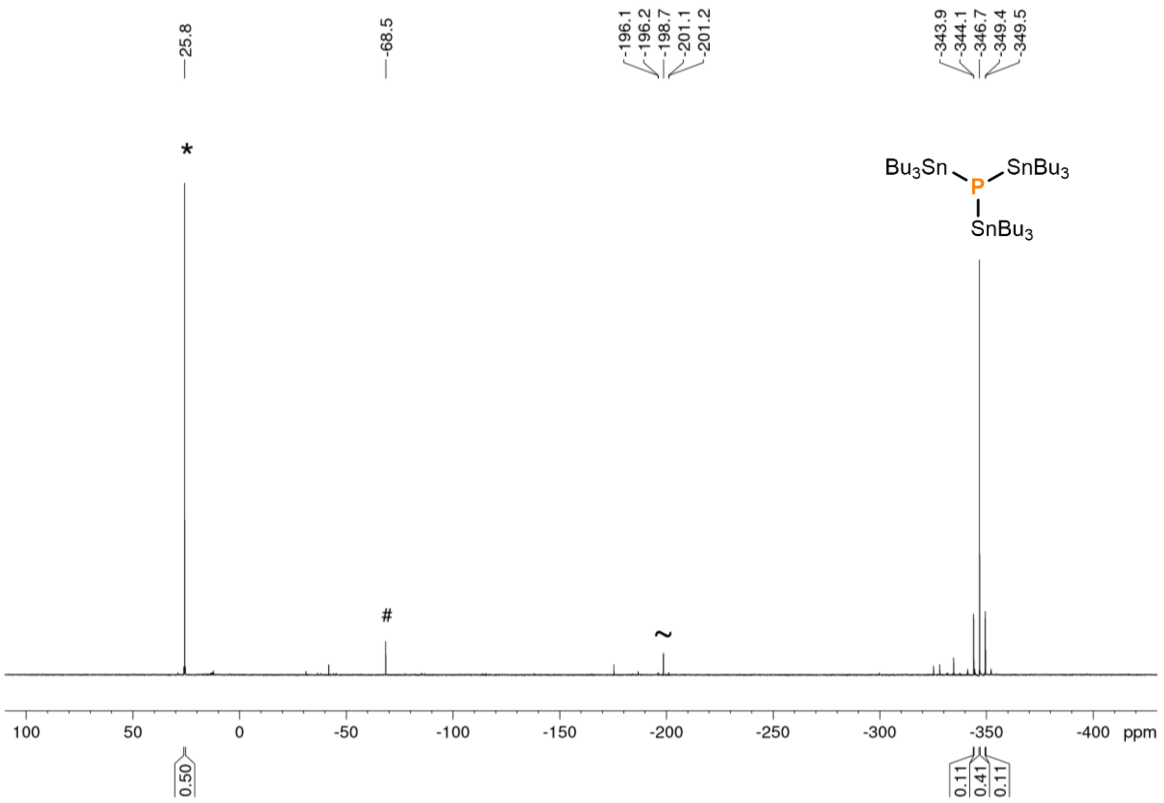

Figure S32. $^{31}P\{^1H\}$ NMR spectrum for the **scale-up** (0.8 mmol) photocatalytic stannylation of P_4 using $(Bu_3Sn)_2$ and photoinitiator anthraquinone (**AQ**). * marks the internal standard Ph_3PO. ~ marks an unknown Sn-containing side product. **#** marks unknown side products.

S9.2 'One-pot' synthesis and purification of $(Ph(O)C)_3P$

To a 100 mL stoppered tube equipped with a stirring bar were added $(Bu_3Sn)_2$ (1.212 mL, 2.4 mmol, 3 equiv. based on phosphorus atoms, 12 equiv. based on P_4) anthraquinone (**AQ**) (20.8 mg, 0.1 mmol, 0.5 equiv. based on P_4) and P_4 (0.2 mmol, as a stock solution in 1.426 mL benzene) in acetone as solvent (2.574 mL, in total 4 mL PhH/acetone mixture). The tube was sealed, placed in a water-cooled block (to ensure a near-ambient temperature was maintained, Figure S31), and irradiated with UV light (365 nm, 14 V, 700 mA, Osram OSLON SSL 80) for 48 h. Subsequent addition of benzoyl chloride PhC(O)Cl (371 µL, 3.2 mmol, 16 equiv. based on P_4, 4 equiv. based on phosphorus atom) and stirring overnight at room temperature resulted in a color change of the orange reaction mixture to a yellow solution (by internal standard addition to a separate reaction the $^{31}P\{^1H\}$ NMR showed 62% conversion into $(Ph(O)C)_3P$, see Figure S33). The volatiles were removed under vacuum and the remaining yellow solid was washed with *n*-hexane (4 x 5 mL). The remaining yellow residue was recrystallized from THF/*n*-hexane at −35°C. Decanting of the mother liquor and washing with *n*-hexane (2 x 10 mL) afforded the desired product $(Ph(O)C)_3P$ was as yellow powder (151.2 mg, 55%).

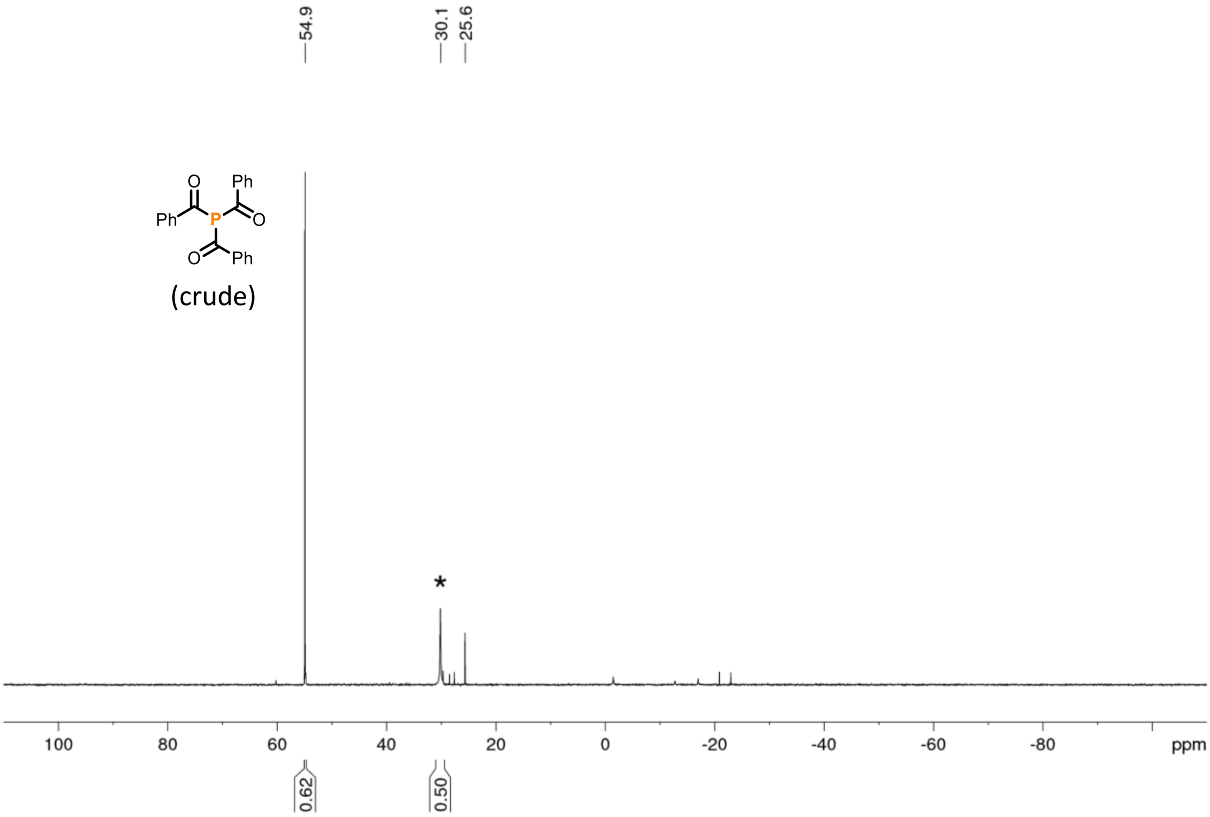

Figure S33. $^{31}P\{^1H\}$ NMR spectrum of **crude** $(Ph(O)C)_3P$ generated *via* photocatalytic stannylation of P_4 in benzene/acetone (**scale-up** (0.8 mmol)) followed by acylation with benzoyl chloride PhC(O)Cl (**1**). * marks the internal standard Ph_3PO (0.02 mmol).

Spectroscopic data of (Ph(O)C)$_3$P:

The NMR data are consistent with previous reports.[0],[5]

^{1}H NMR (400.13 MHz, 300 K, C_6D_6): δ = 7.98 ppm (2H, m), 7.02 ppm (1H, t, $^3J(^1H-^1H)$ = 7.3 Hz), 6.95 ppm (2H, t, $^3J(^1H-^1H)$ = 7.5 Hz).

$^{31}P\{^1H\}$ NMR (161.98 MHz, 300 K, C_6D_6): δ = 54.3 ppm (s).

$^{13}C\{^1H\}$ NMR (100.61 MHz, 300 K, C_6D_6): δ = 205.3 ppm (d, $J(^{31}P-^1H)$ = 35.4 Hz), 140.5 ppm (d, $J(^{31}P-^1H)$ = 35.3 Hz), 133.5 ppm (d, $J(^{31}P-^1H)$ = 1.2 Hz), 128.6 ppm (d, $J(^{31}P-^1H)$ = 8.0 Hz), 128.5 ppm (d, $J(^{31}P-^1H)$ =0.8 Hz).

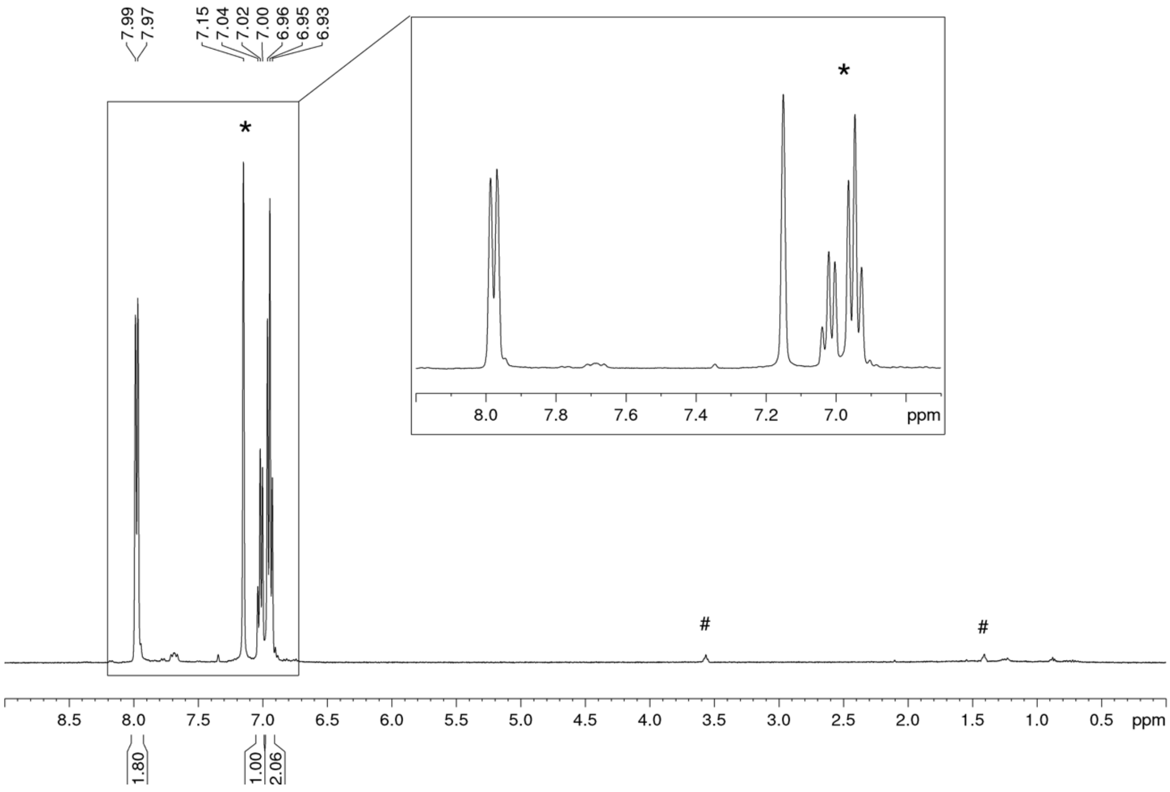

Figure S34. ^{1}H NMR (400.13 MHz, 300 K, C_6D_6) spectrum of (Ph(O)C)$_3$P. * marks C_6D_6. # marks minor THF.

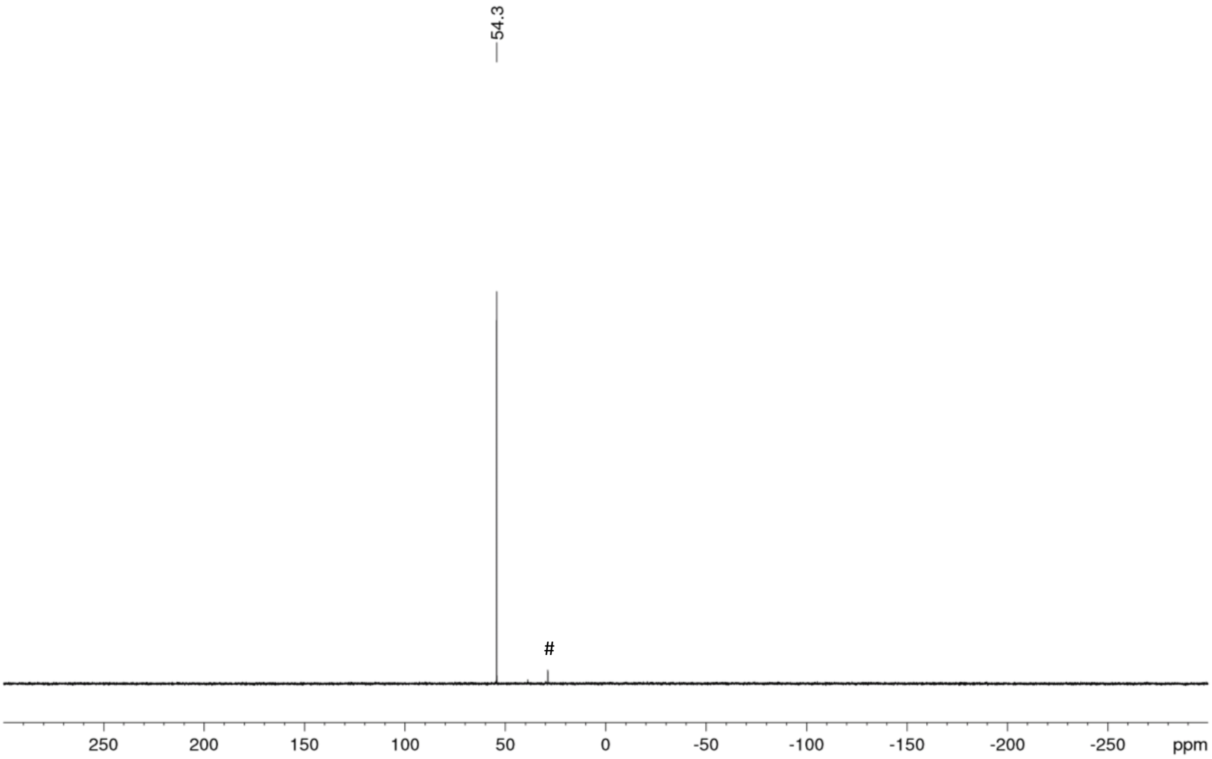

Figure S35. $^{31}P\{^1H\}$ NMR (161.98 MHz, 300 K, C_6D_6) spectrum of $(Ph(O)C)_3P$. # marks minor unknown impurity.

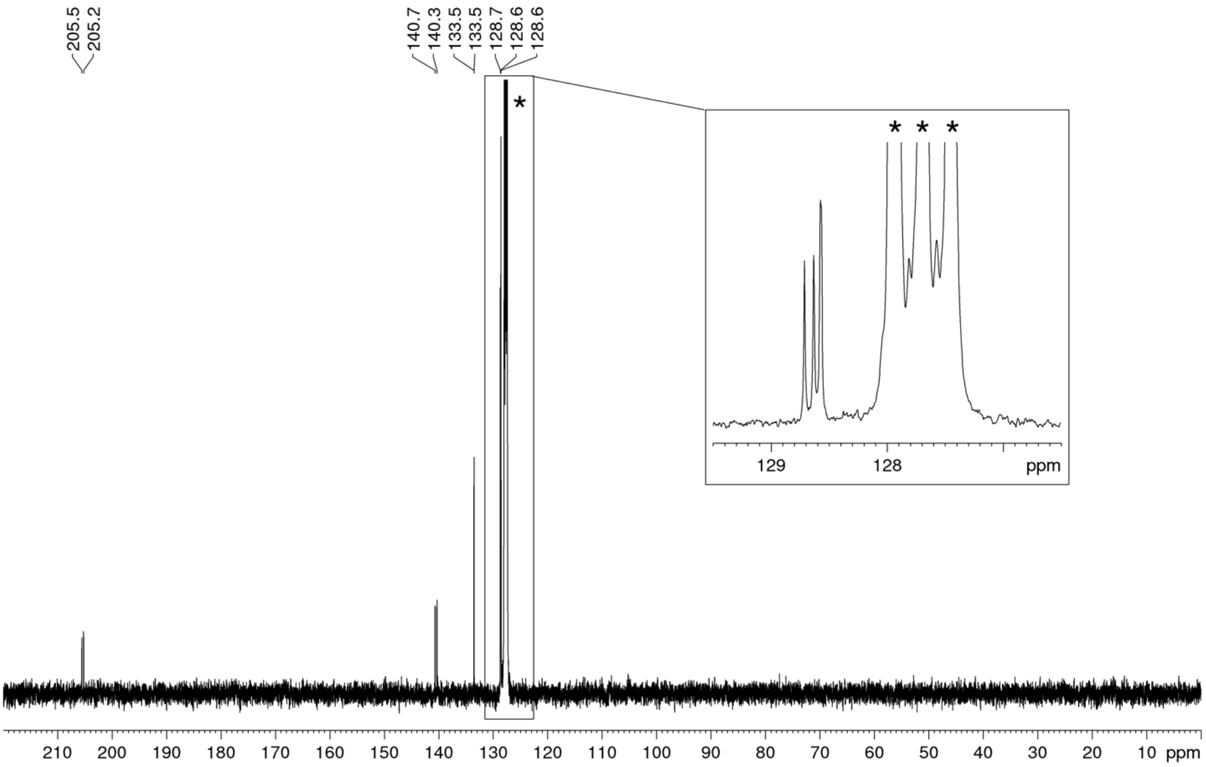

Figure S36. $^{13}C\{^1H\}$ NMR (100.61 MHz, 300 K, C_6D_6) spectrum of $(Ph(O)C)_3P$. * marks C_6D_6.

S9.3 'One-pot' synthesis and purification of phosphonium salt $[Bn_4P]Br$

To a 100 mL stoppered tube equipped with a stirring bar were added $(Bu_3Sn)_2$ (1.212 mL, 2.4 mmol, 3 equiv. based on phosphorus atoms, 12 equiv. based on P_4) anthraquinone (20.8 mg, 0.1 mmol, 0.5 equiv. based on P_4) and P_4 (0.2 mmol, as a stock solution in 1.426 mL benzene) in acetone as solvent (2.574 mL, in total 4 mL PhH/acetone mixture). The tube was sealed, placed in a water-cooled block (to ensure a near-ambient temperature was maintained, Figure S31), and irradiated with UV light (365 nm, 14 V, 700 mA, Osram OSLON SSL 80) for 48 h. Subsequent addition of benzyl bromide (475 µL, 4 mmol, 20 equiv. based on P_4, 5 equiv. based on phosphorus atoms) and heating overnight at 60 °C resulted in a color change of the orange reaction mixture to a yellow solution (by internal standard addition to a separate reaction the $^{31}P\{^1H\}$ NMR showed 67% conversion into $[Bn_4P]Br$, see Figure S37). The volatiles were removed under vacuum. *n*-Hexane was added to the remaining orange oil and the product was precipitated and washed with *n*-hexane (2 x 10 mL). Recrystallization twice from acetone/*n*-hexane afforded the desired product $[Bn_4P]Br$ (212.2 mg, 56%) as an off-white powder.

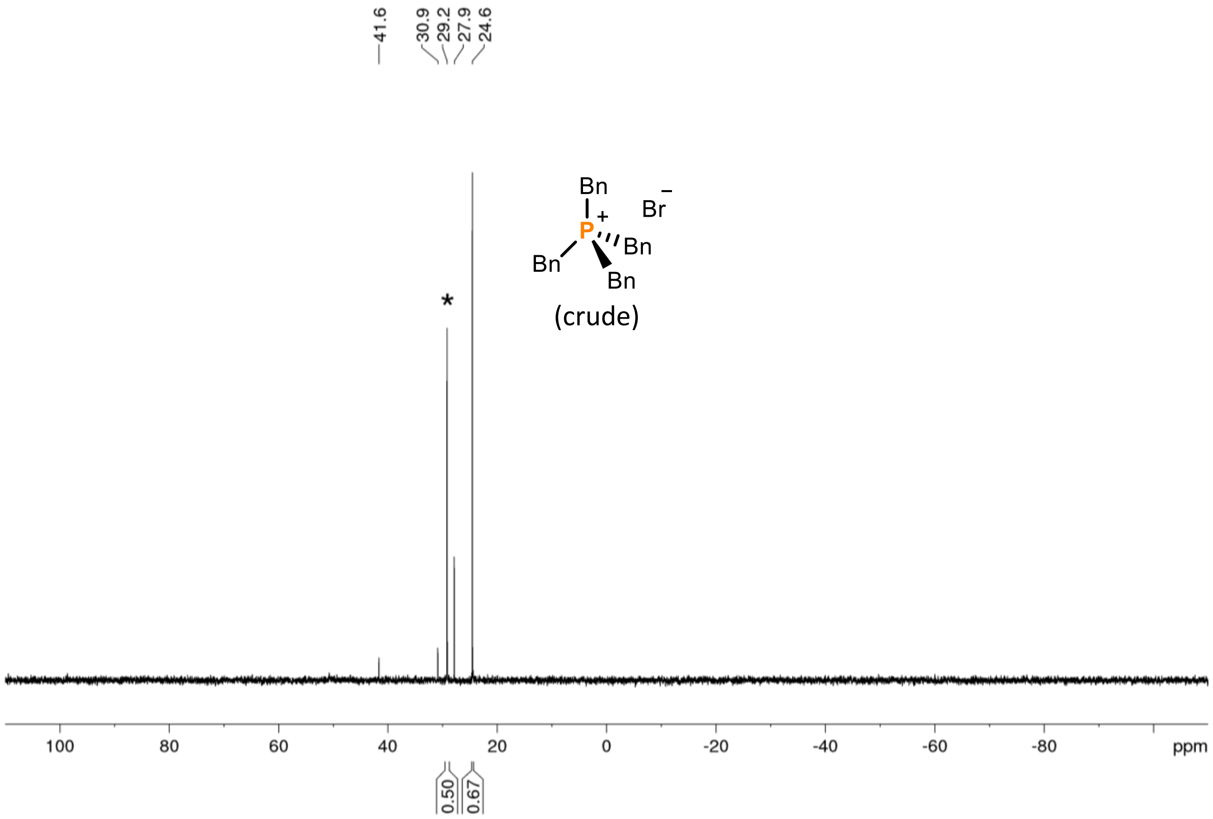

Figure S37. Quantitative $^{31}P\{^1H\}$ NMR (zgig) spectrum of **crude** $[Bn_4P]Br$ generated *via* photocatalytic stannylation of P_4 in benzene/acetone followed by alkylation with benzyl bromide (BnBr). * marks the internal standard Ph_3PO (0.02 mmol).

Spectroscopic data of $[Bn_4P]Br$:

The NMR data are consistent with previous reports of both the chloride salt $[Bn_4P]Cl$[6] and $[Bn_4P]Br$.[1]

^{1}H NMR (400.13 MHz, 300 K, $CDCl_3$): δ = 7.32 ppm (3H, m), 7.18 ppm (2H, m), 4.03 ppm (2H, d, $^2J(^{31}P-^1H)$ = 14.3 Hz).

$^{31}P\{^1H\}$ NMR (161.98 MHz, 300 K, $CDCl_3$): δ = 24.7 ppm (s).

^{31}P NMR (161.98 MHz, 300 K, $CDCl_3$): δ = 24.7 ppm (nonet, $^2J(^{31}P-^1H)$ = 14.3 Hz).

$^{13}C\{^1H\}$ NMR (100.61 MHz, 300 K, $CDCl_3$): δ = 130.6 ppm (d, $J(^{31}P-^1H)$ = 5.3 Hz), 129.6 ppm (d, $J(^{31}P-^1H)$ = 2.9 Hz), 128.7 ppm (d, $J(^{31}P-^1H)$ = 3.5 Hz), 127.5 ppm (d, $J(^{31}P-^1H)$ = 8.1Hz), 26.8 ppm (d, $J(^{31}P-^1H)$ = 43.2 Hz).

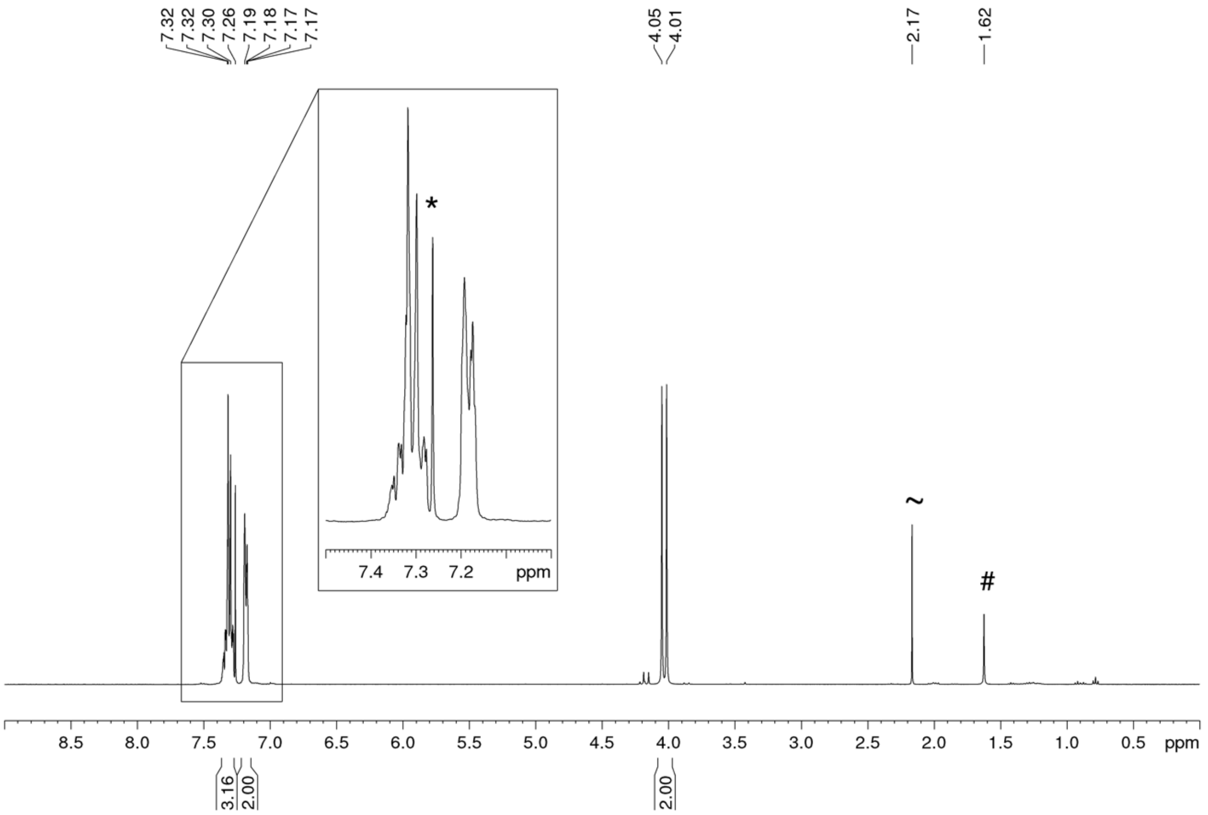

Figure S38. ^{1}H NMR (400.13 MHz, 300 K, $CDCl_3$) spectrum of $[Bn_4P]Br$. * marks $CDCl_3$. ~ marks residual acetone. # marks water present in the $CDCl_3$ NMR solvent.

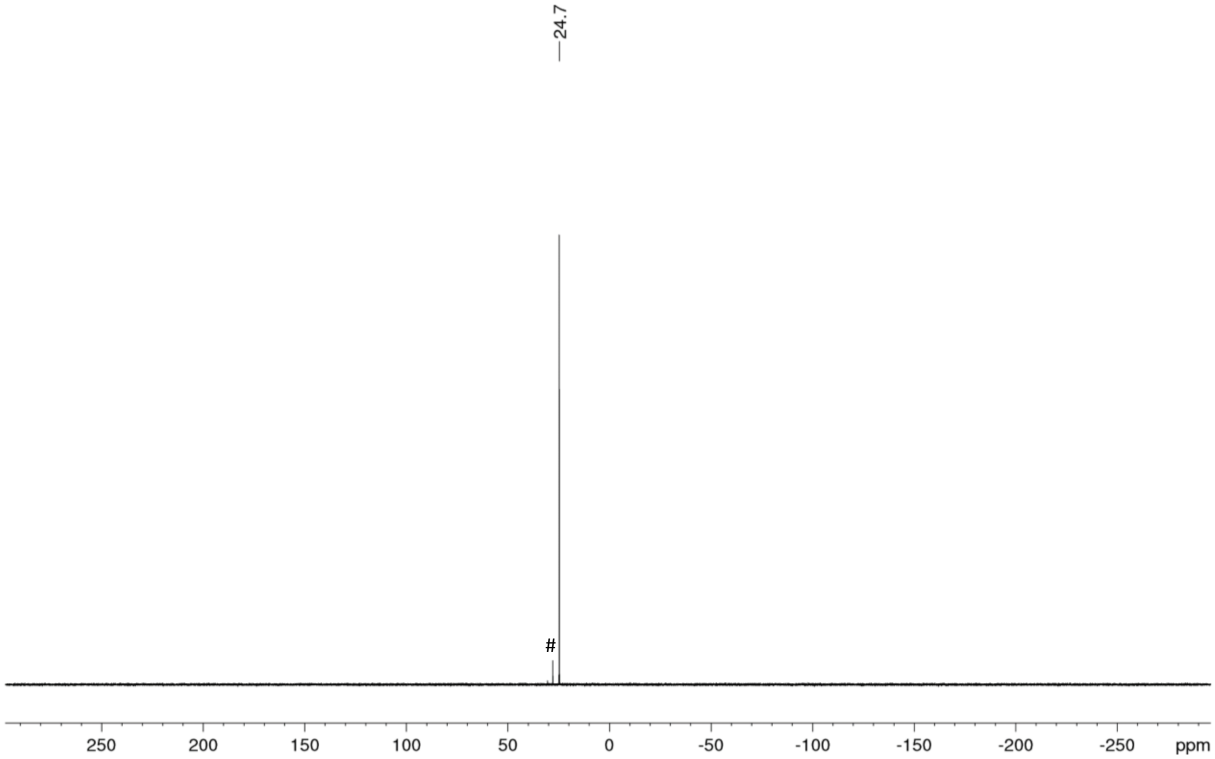

Figure S39. $^{31}P\{^{1}H\}$ NMR (161.98 MHz, 300 K, $CDCl_3$) spectrum of $[Bn_4P]Br$. # marks minor unknown impurity.

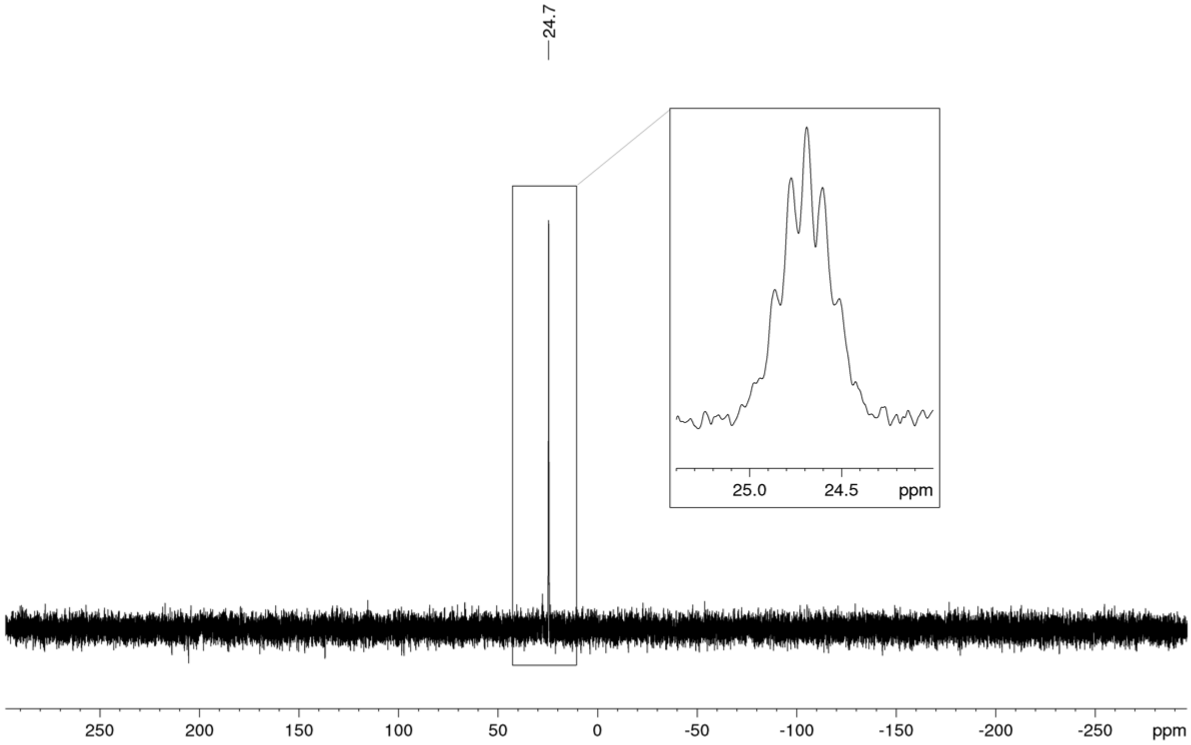

Figure S40. ^{31}P NMR (161.98 MHz, 300 K, $CDCl_3$) spectrum of $[Bn_4P]Br$.

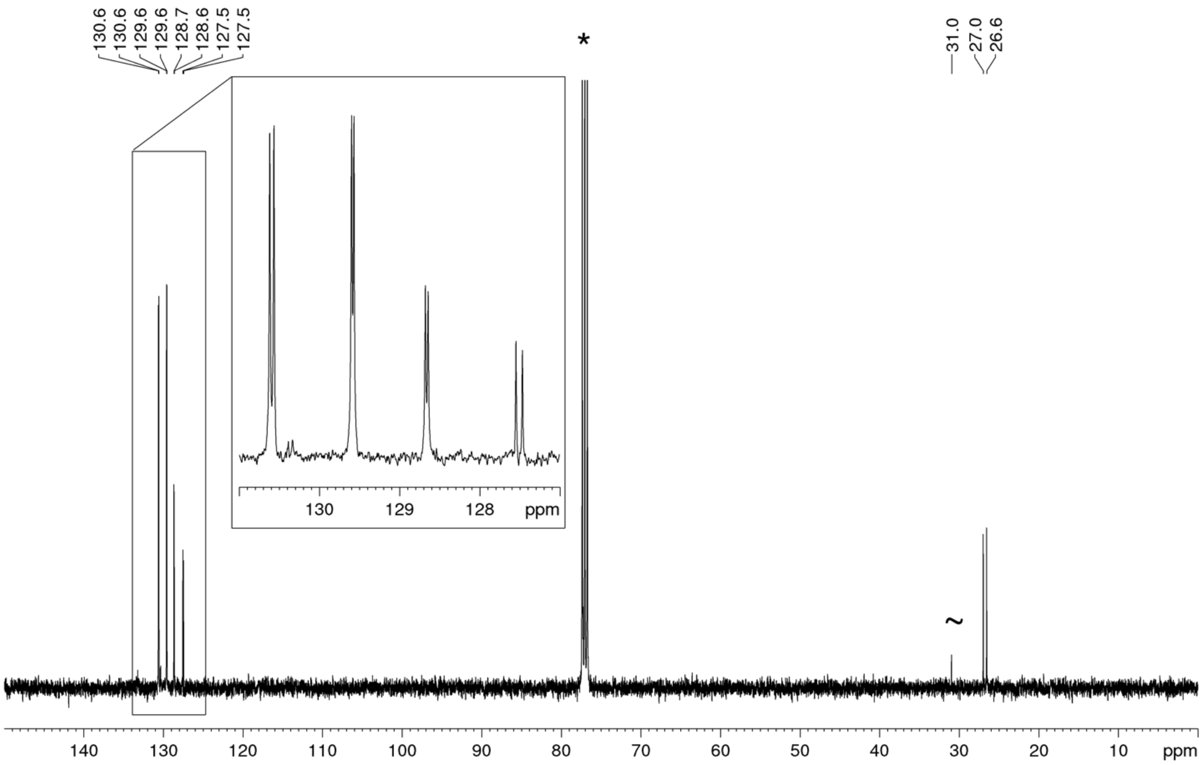

Figure S41. $^{13}C\{^{1}H\}$ NMR (100.61 MHz, 300 K, $CDCl_3$) spectrum of $[Bn_4P]Br$. * marks C_6D_6. ~ marks residual acetone.

S9.4 'One-pot' synthesis and purification of THPC and recycling of Bu_3SnCl and $(Bu_3Sn)_2$

To a 100 mL stoppered tube equipped with a stirring bar were added $(Bu_3Sn)_2$ (1.212 mL, 2.4 mmol, 3 equiv. based on phosphorus atoms, 12 equiv. based on P_4), anthraquinone (20.8 mg, 0.1 mmol, 0.5 equiv. based on P_4) and P_4 (0.2 mmol, as a stock solution in 1.426 mL benzene) in acetone as solvent (2.574 mL, in total 4 mL PhH/acetone mixture). The tube was sealed, placed in a water-cooled block (to ensure a near-ambient temperature was maintained, Figure S31), and irradiated with UV light (365 nm, 14 V, 700 mA, Osram OSLON SSL 80) for 48 h. Following removal of volatiles under vacuum, EtOH (5 ml) and paraformaldehyde (300 mg, 10 mmol, 50 equiv. based on P_4, 12.5 equiv. based on phosphorus atoms) were added, and the resulting suspension was stirred at room temperature for 16 h. The mixture was frozen in a liquid-nitrogen bath, and HCl (4.0 M in 1,4-dioxane, 2 ml, 8 mmol (based on P_4)) was added. After thawing, the yellowish reaction mixture was stirred at room temperature for 2 h and volatiles were removed under vacuum. The remaining orange oily solid residue was washed with Et_2O (3 x 10 ml) to extract the Sn-containing compounds, Bu_3SnCl and $(Bu_3Sn)_2$. The residue was recrystallized from EtOH/*n*-hexane (4 mL/4 mL) at −35°C. Decanting of the mother liquor afforded the desired product THPC as a pale yellow solid (50.3 mg, 33%) after drying under vacuum. The combined Et_2O washes from the above reaction were dried under vacuum to afford a mixture of Bu_3SnCl and $(Bu_3Sn)_2$ as a yellow oil (1.346 g). ^1H and $^{119}Sn\{^1H\}$ NMR indicate a ratio of 1 : 1.3 for the $(Bu_3Sn)_2$ / Bu_3SnCl mixture (Figure S45 and S46). Overall, 92% of the used $(Bu_3Sn)_2$ (2.4 mmol) could be recovered as a mixture (1.346 g) of Bu_3SnCl (567.7 mg, 1.74 mmol, 36%) and $(Bu_3Sn)_2$ (778.3 mg, 1.34 mmol, 56%).

Spectroscopic data of THPC:

The NMR data are consistent with previous reports.[0][7]

^{1}H NMR (400.13 MHz, 300 K, D_2O): δ = 4.67 ppm (d, $^2J(^{31}P\text{-}^1H)$ = 1.8 Hz).

$^{31}P\{^1H\}$ NMR (161.98 MHz, 300 K, D_2O): δ = 27.1 ppm (s).

$^{13}C\{^1H\}$ NMR (100.61 MHz, 300 K, D_2O): δ = 49.1 ppm (d, $^1J(^{31}P\text{-}^{13}C)$ = 51.3 Hz).

Spectroscopic data of the recycled mixture of Bu_3SnCl and $(Bu_3Sn)_2$:

The NMR data of Bu_3SnCl[8] and $(Bu_3Sn)_2$[9] are consistent with previous reports. The ^1H NMR and $^{119}Sn\{^1H\}$ NMR showed a **1 to 1.3 ratio of $(Bu_3Sn)_2$ to Bu_3SnCl.**

^{1}H NMR (400.13 MHz, 300 K, C_6D_6): δ = 1.70-1.62 (m, 2H, $(Bu_3Sn)_2$), 1.60-1.53 (m, 1.3H, Bu_3SnCl), 1.46-1.36 (m, 2.1H, $(Bu_3Sn)_2$), 1.30-1.21 (m, 1.5H, Bu_3SnCl), 1.16-1.12 (m, 2H, $(Bu_3Sn)_2$), 1.10-1.07 (m, 1.5H, Bu_3SnCl), 0.96 (t, 3H, $(Bu_3Sn)_2$, $^3J(^1H\text{-}^1H)$ = 7.3 Hz), 0.85 (t, 2H, Bu_3SnCl, $^3J(^1H\text{-}^1H)$ = 7.3 Hz).

$^{119}Sn\{^1H\}$ NMR (149.21 MHz, 300 K, C_6D_6): δ = −83.2 ppm (s, 2Sn of $(Bu_3Sn)_2$, $^1J(^{119}Sn\text{-}^{13}C)$ = 240.7 Hz, $^1J(^{119}Sn\text{-}^{119}Sn)$ = 2556.3 Hz, 146.9 ppm (s, 1.3Sn of Bu_3SnCl).

$^{13}C\{^1H\}$ NMR (100.61 MHz, 300 K, C_6D_6): δ = 30.8 (s, $(Bu_3Sn)_2$), 27.8 (s, Bu_3SnCl), 27.6 (s, $(Bu_3Sn)_2$), 26.8 (s, Bu_3SnCl), 16.9 (s, Bu_3SnCl), 13.6 (s, $(Bu_3Sn)_2$), 13.4 (s, Bu_3SnCl), 10.1 (s, $(Bu_3Sn)_2$).

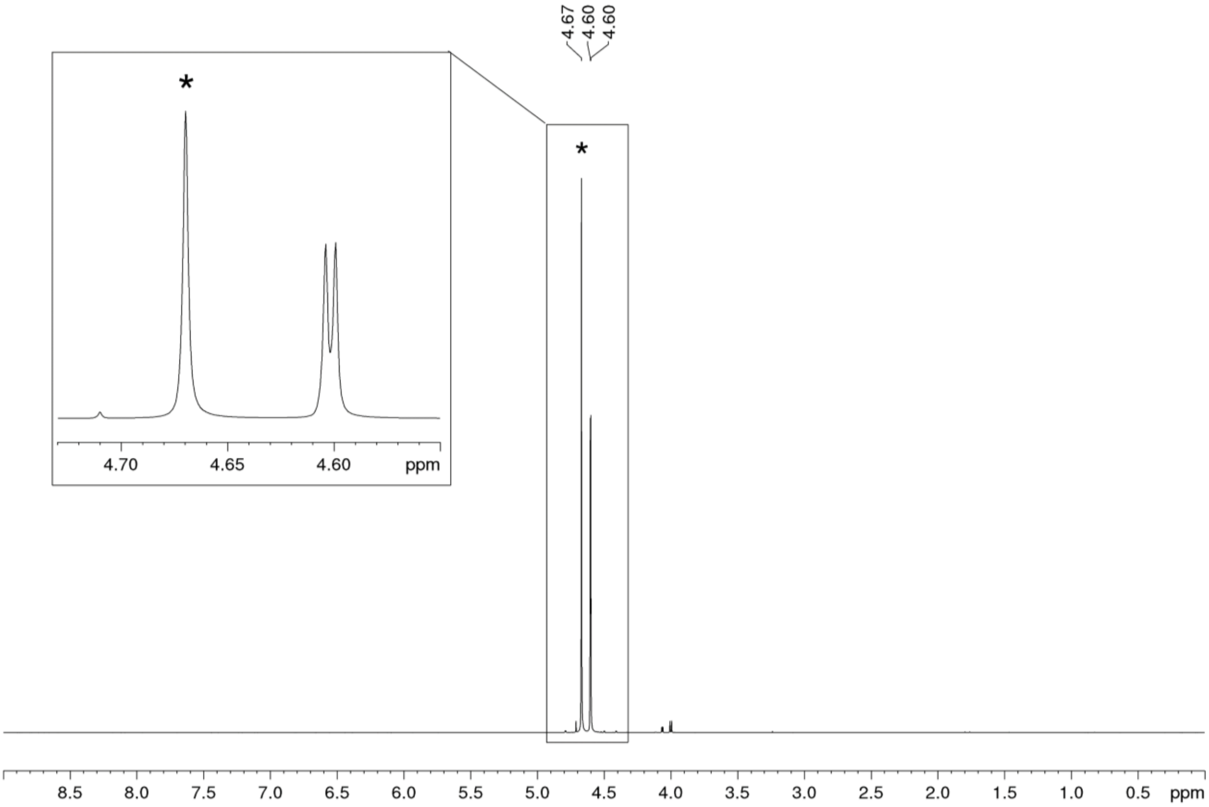

Figure S42. ^1H NMR (400.13 MHz, 300 K, D_2O) spectrum of THPC. * marks D_2O.

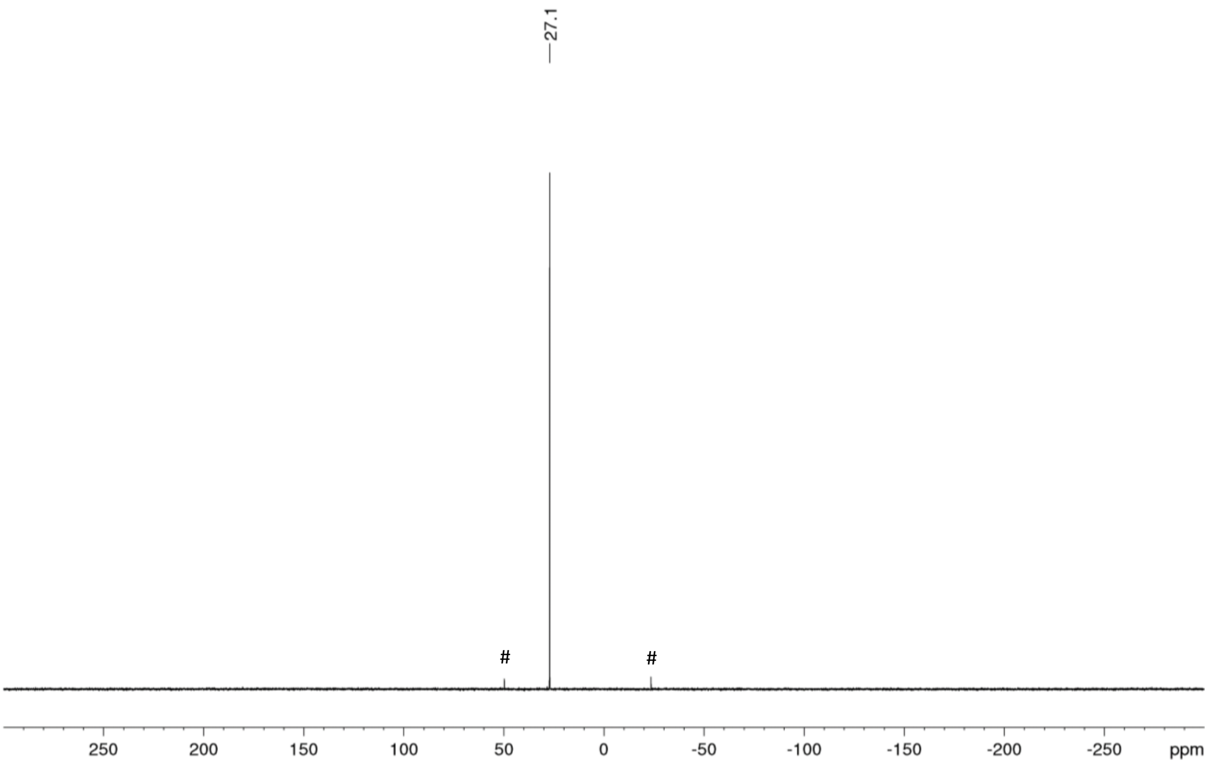

Figure S43. $^{31}P\{^1H\}$ NMR (161.98 MHz, 300 K, D_2O) spectrum of THPC. # marks minor unknown impurities.

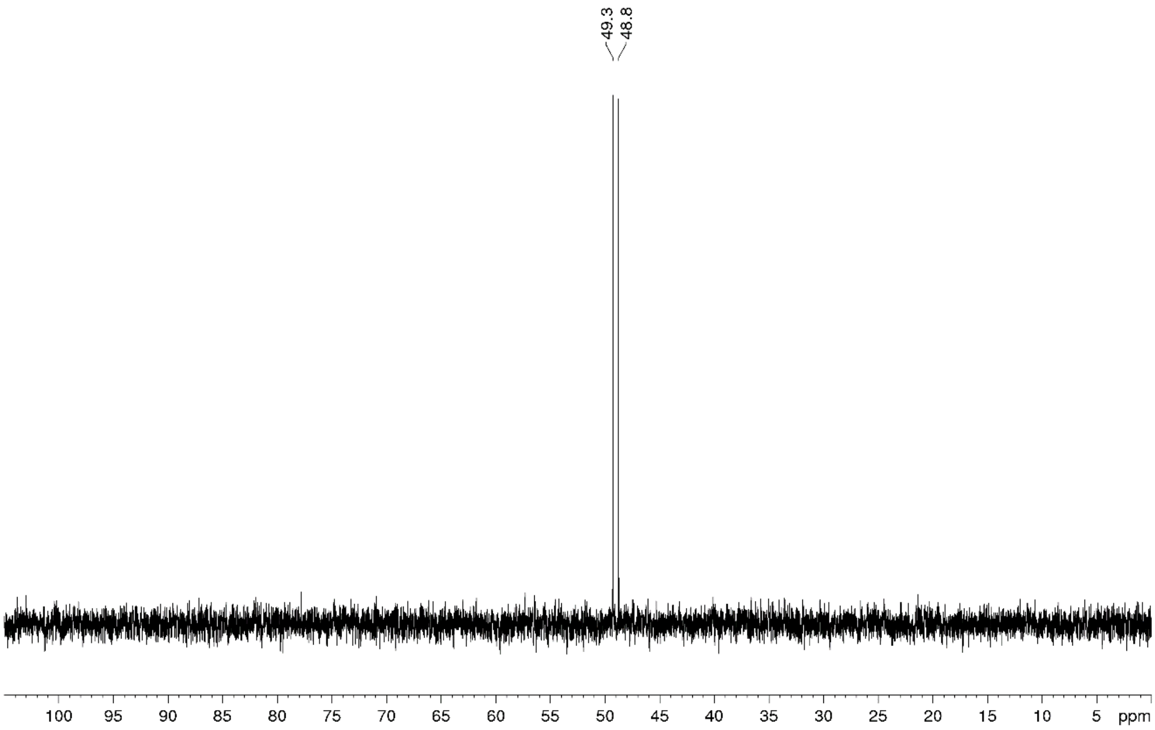

Figure S44. $^{13}C\{^{1}H\}$ NMR (100.61 MHz, 300 K, D_2O) spectrum of THPC.

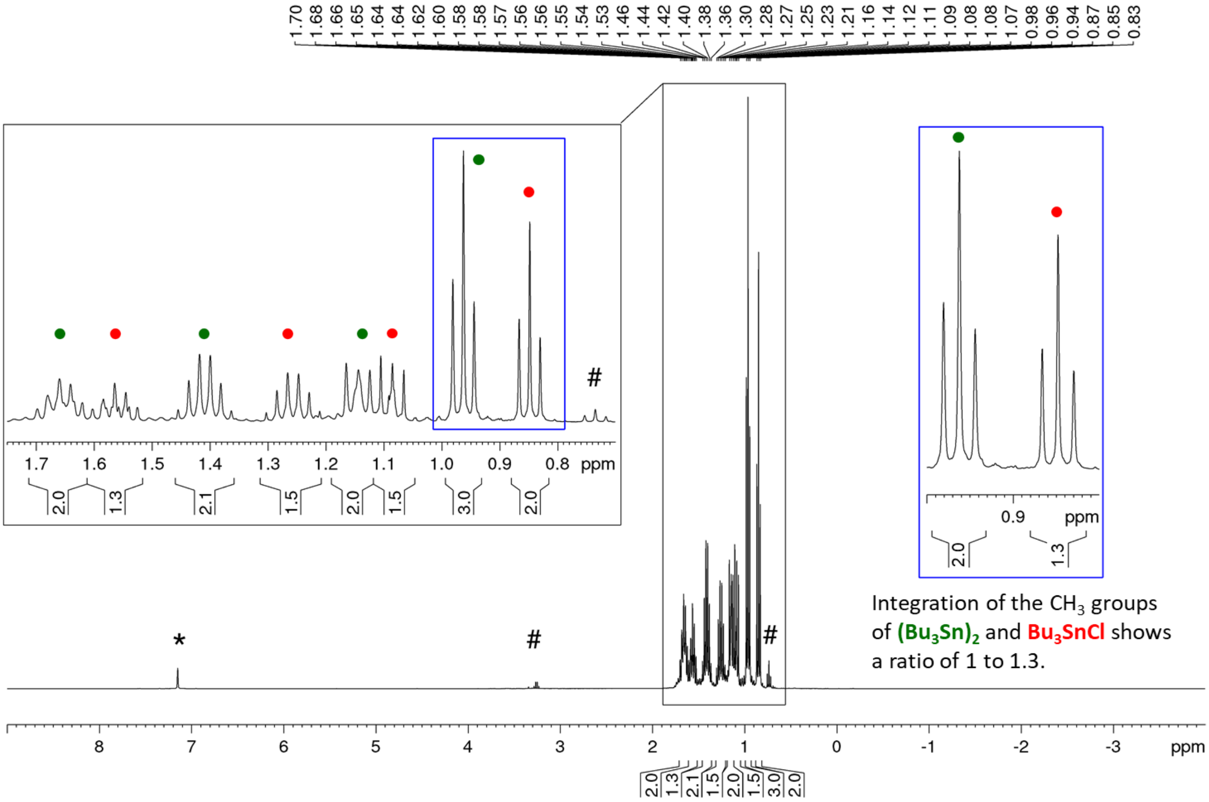

Figure S45. ^{1}H NMR (400.13 MHz, 300 K, C_6D_6) spectrum of recycled $\mathbf{Bu_3SnCl}$ and $\mathbf{(Bu_3Sn)_2}$ mixture. * marks C_6D_6. # marks minor Et_2O. The blue box shows the integration of the CH_3 groups of $\mathbf{(Bu_3Sn)_2}$ and $\mathbf{Bu_3SnCl}$, which indicates a 1 to 1.3 ratio of the Sn-containing compounds.

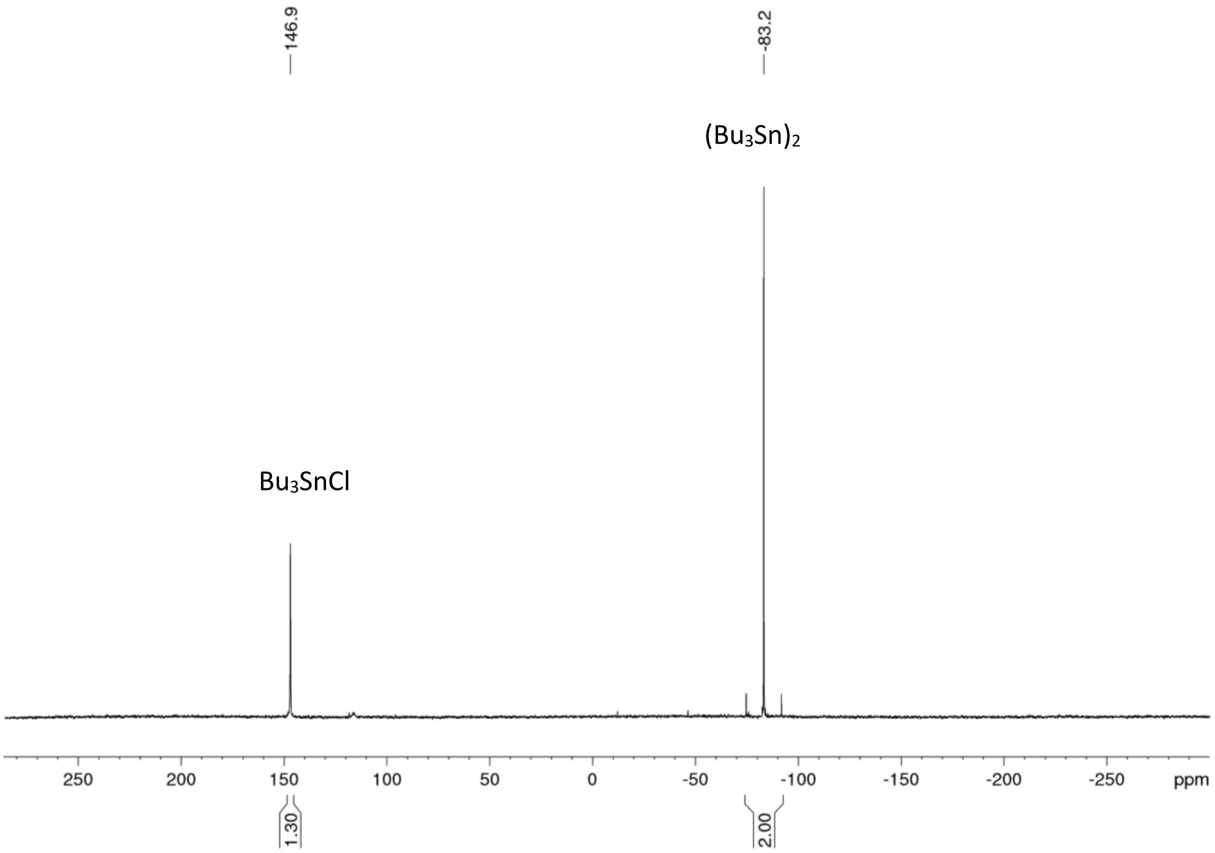

Figure S46. $^{119}Sn\{^1H\}$ NMR (149.21 MHz, 300 K, C_6D_6) spectrum of recycled Bu_3SnCl and $(Bu_3Sn)_2$ mixture (ratio of 1.3 to 1).

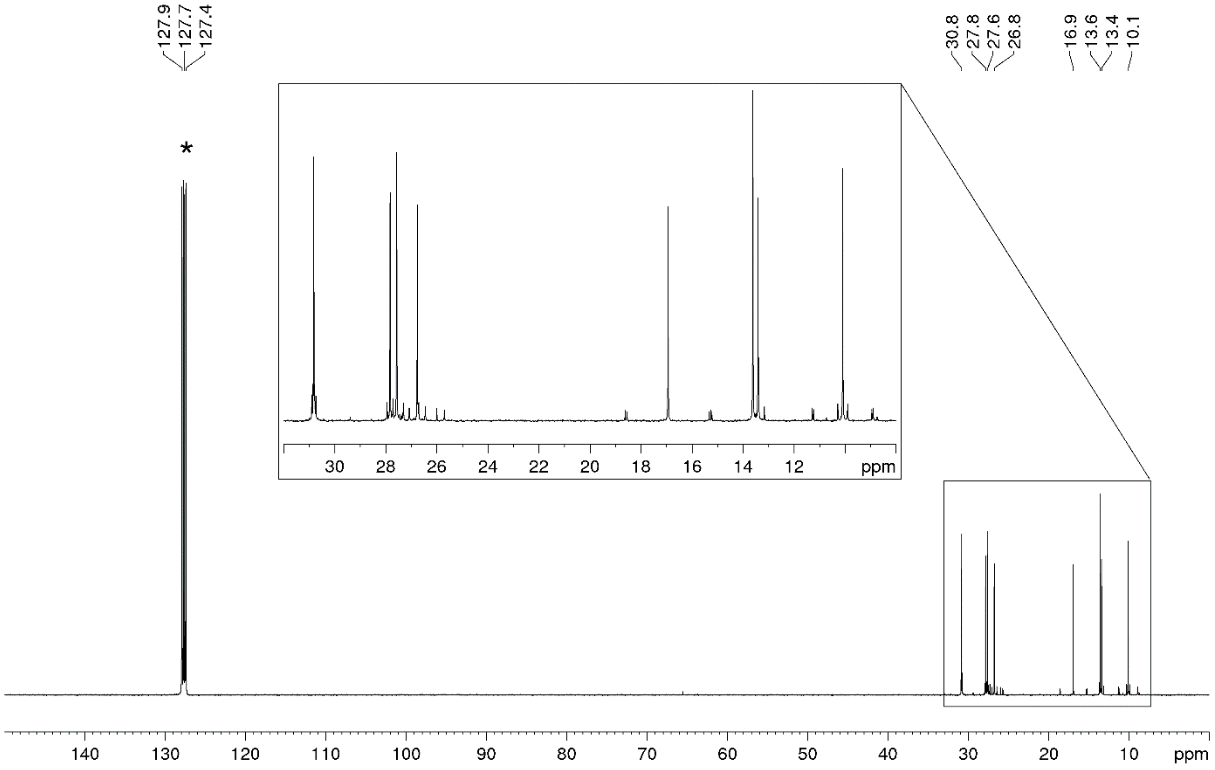

Figure S47. $^{13}C\{^1H\}$ NMR (100.61 MHz, 300 K, C_6D_6) spectrum of recycled Bu_3SnCl and $(Bu_3Sn)_2$ mixture. * marks C_6D_6.

S10. References

1. D. J. Scott, J. Cammarata, M. Schimpf and R. Wolf, *Nat. Chem.*, 2021, **13**, 458–464.
2. a) U. Lennert, P. B. Arockiam, V. Streitferdt, D. J. Scott, C. Rödl, R. M. Gschwind and R. Wolf, *Nat. Catal.*, 2019, **2**, 1101–1106; b) M. Till, V. Streitferdt, D. J. Scott, M. Mende, R. M. Gschwind and R. Wolf, *Chem. Commun.,* 2022, **58**, 1100–1103.
3. a) W. P. Neumann, H. Hillgärtner, K. M. Baines, R. Dicke, K. Vorspohl, U. Kobs and U. Nussbeutel, *Tetrahedron*, 1989, **45**, 951–960; b) D. J. Hart, R. Krishnamurthy, L. M. Pook and F. L. Seely, *Tetrahedron Lett.*, 1993, **34**, 7819–7822; c) M. J. Tomaszeski and J. Warkentin, *J. Chem. Soc., Chem. Commun.,* 1993, **18**, 1407–1408.a
4. J. A. Dantas, J. T. M. Correia, M. W. Paixão and A. G. Corrêa, *ChemPhotoChem*, 2019, **3**, 506–520.
5. G. D. Macdonnell, A. Radhakrishna, K. D. Berlin, J. Barycki, R. Tyka and P. Mastalerz, *Tetrahedron Lett.*, 1978, **19**, 857–860.
6. M. B. Geeson, P. Ríos, W. J. Transue and C. C. Cummins, *J. Am. Chem. Soc.*, 2019, **141**, 6375–6384.
7. W. J. Vullo, *J. Org. Chem.,* 1968, **33**, 3665–3667.
8. A. G. Davies, A. Sella and R. Sivasubramaniam, *J. Organomet. Chem.*, 2006, **691**, 3556–3561.
9. a) A. Darwish and J. M. Chong, *Syn. Comm.*, 2004, **34**, 1885–1890; b) T. N. Mitchell, *Encyclopedia of Reagents for Organic Synthesis – Hexabutyldistannane* (Wiley), 2012; c) T. N. Mitchell and G. Walter, *J. Chem. Soc., Perkin Trans.*, 1977, **2**, 1842–1847.
